# Comprehensive and robust stability-indicating reversed phase high performance liquid chromatography (RP-HPLC) method for Rivaroxaban: synergistic integration of infrared spectroscopy and clinical pharmacology insights

**DOI:** 10.3389/fchem.2025.1551189

**Published:** 2025-04-25

**Authors:** Aktham Mestareehi

**Affiliations:** ^1^ Department of Applied Pharmaceutical Sciences and Clinical Pharmacy, Faculty of Pharmacy, Isra University, Amman, Jordan; ^2^ Department of Pharmaceutical Sciences, School of Pharmacy and Health Sciences, Wayne State University, Detroit, MI, United States; ^3^ Department of Pharmaceutical Sciences, School of Pharmacy, Northeastern Illinois University, Chicago, IL, United States

**Keywords:** Rivaroxaban, high-performance liquid chromatography (HPLC), specificity, linearity, accuracy, precision, limit of detection (LOD), limit of quantitation (LOQ)

## Abstract

**Introduction:**

Rivaroxaban is an anticoagulant medication that targets a key stage in the blood clotting process, preventing the formation and growth of clots. It is commonly used to prevent thrombosis or inhibit the enlargement of existing clots. Rivaroxaban functions as a Factor Xa inhibitor and is indicated for reducing the risk of stroke and systemic embolism in patients with non-valvular atrial fibrillation, treating deep vein thrombosis (DVT) and pulmonary embolism (PE), as well as reducing the risk of recurrent DVT and PE, and prophylaxis of DVT, which may lead to PE in patients undergoing knee or hip replacement surgery.

**Methods:**

A robust, precise, and selective reversed-phase high-performance liquid chromatography (HPLC) method was developed and validated for analyzing Rivaroxaban in raw materials. Isocratic elution at a flow rate of 1 mL/min was performed using a Thermo ODS Hypersil C18 column (4.6 × 250 mm, 5 µm) at ambient temperature. The mobile phase consisted of monobasic potassium phosphate at pH 2.9 and acetonitrile in a 70:30 (v/v) ratio, with UV detection at 249 nm.

**Results:**

Linearity was established in the concentration range of 50–1,000 ppm (R^2^ = 0.999), and the retention time for Rivaroxaban was approximately 12 min. The percentage relative standard deviation (RSD) for precision and accuracy was consistently below 2.0%, ensuring method reliability. Solution stability studies confirmed the stability of Rivaroxaban over the analysis period, as no peak loss, degradation, or additional peaks were observed between the first and last injections. Furthermore, forced degradation studies were conducted under various stress conditions, including acid and base hydrolysis, as well as hydrogen peroxide oxidation. The method successfully resolved Rivaroxaban from its degradation products, demonstrating its stability-indicating capability.

**Conclusion:**

Rivaroxaban is a novel oral anticoagulant that selectively and directly inhibits factor *Xa*. A method has been developed and validated for its analysis, adhering to guidelines from the International Conference on Harmonisation (ICH) and the U.S. Pharmacopeia (USP). The validation process assessed parameters such as specificity, robustness, linearity, accuracy, precision, limit of detection (LOD), and limit of quantitation (LOQ). The LOD for impurities and degradants was determined to be 0.3 ppm, while the LOQ was 1 ppm. This stability-indicating method is highly suitable for routine quality control and analytical applications in both raw materials and finished drug products, owing to its simplicity, efficiency, and robustness.

## Introduction

Rivaroxaban is an oral, oxazolidinone based anticoagulant that acts as a selective, direct inhibitor of Factor Xa. It is commonly prescribed to prevent venous thromboembolism in adult patients following total knee or hip replacement surgery (Janssen Pharmaceuticals, 2011). Rivaroxaban was originally developed by Bayer and is marketed in the United States by Janssen Pharmaceuticals, a division of Johnson and Johnson. It was the first orally administered direct factor Xa inhibitor to become available (Fernandez et al., 2021). Rivaroxaban is rapidly absorbed, reaching peak plasma concentrations within 2–4 h after tablet administration. Its oral bioavailability is high (80%–100%) for the 10 mg dose, regardless of food intake, and is similarly high for the 15 mg and 20 mg doses when taken with food (Janssen Pharmaceuticals, 2011). A synthetic form of Rivaroxaban has been developed, utilizing (R)-epichlorohydrin as a key chiral intermediate (R)-Epichlorohydrin was reacted with sodium cyanate (NaOCN) to produce (R)-chloromethyl-2-oxazolidinone, with bromobenzene serving as the primary starting material (Yuan et al., 2014).

**FIGURE 1:**
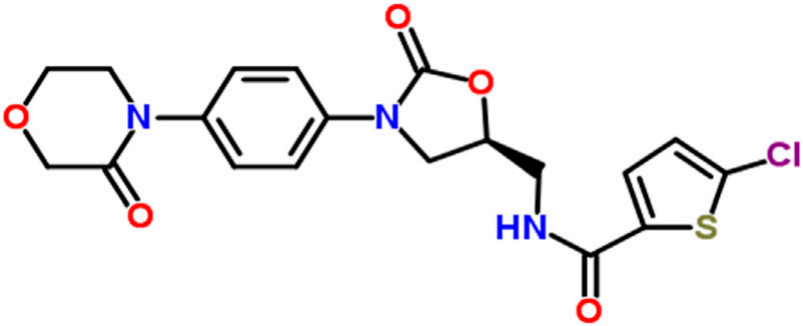
The chemical structure of Rivaroxaban (National Center for Biotechnology Information, 2024).

Rivaroxaban, the active ingredient in XARELTO tablets, has the molecular formula C_19_H_18_ClN_3_O_5_S and a molecular weight of 435.89 g/mol. Its structural formula is shown in Figure 1 and purity ≥99. Rivaroxaban’s chemical name is (5-Chloro-N-({(5S)-2-oxo-3-[4-(3-oxomorpholin-4-yl) phenyl]-1,3-oxazolidin-5-yl} methyl) thiophene-2- carboxamide) (Yuan et al., 2014). It is an odorless, non-hygroscopic, white to yellowish powder that exists as a pure (S)-enantiomer. It has a melting point of 228°C–229°C and a boiling point of approximately 732.6°C ± 60.0°C. The compound’s density is 1.460 g/cm^3^. It exhibits an optical rotation [α]D of −34 to −44 (c = 0.3 in DMSO) and has a pKa of 13.36 (Mueck et al., 2014). Classified under the Biopharmaceutical Classification System as a low-solubility, high-permeability compound (Class 2), rivaroxaban has limited pH-independent solubility in aqueous media (5–7 mg/L; pH 1–9) but is slightly soluble in polyethylene glycol 400 (2,431 mg/L) (Mueck et al., 2014). Additionally, each XARELTO tablet contains 10 mg, 15 mg, or 20 mg of Rivaroxaban. Inactive ingredients include Hypromellose, magnesium stearate, lactose monohydrate, croscarmellose sodium, microcrystalline cellulose, and sodium lauryl sulfate. Additionally, each tablet is coated with a film coating mixture tailored for the 10 mg, 15 mg, and 20 mg strengths (Janssen Pharmaceuticals, 2011; FDA, 2014).

## Clinical pharmacology

Xarelto (Rivaroxaban) is an anticoagulant that works by directly inhibiting factor Xa, a key enzyme in the blood coagulation cascade. By blocking factor Xa, Xarelto prevents the conversion of prothrombin to thrombin, thereby inhibiting clot formation. Since it does not require a cofactor like anti-thrombin III, it is considered a direct and specific factor Xa inhibitor. This mechanism makes Xarelto effective in preventing and treating conditions like deep vein thrombosis (DVT), pulmonary embolism (PE), and stroke in patients with non-valvular atrial fibrillation (Weitz and Bates, 2005).

The absolute bioavailability of Xarelto 10 mg is 80%–100%, unaffected by food, so it can be taken with or without food. For the 20 mg dose, bioavailability is 66% but taking it with food increases bioavailability significantly (39% increase in AUC and 76% increase in Cmax). Therefore, Xarelto 15 mg and 20 mg must be taken with the evening meal. The peak plasma concentrations (Cmax) of Rivaroxaban occur 2–4 h after intake (Mueck et al., 2011).

Rivaroxaban has a plasma protein binding of approximately 92%–95%, with albumin being the main protein binding component. The steady-state volume of distribution in healthy individuals is around 50 L. (Janssen Pharmaceuticals, 2011) About 51% of an administered dose is recovered as metabolites, with 30% excreted in the urine and 21% in feces. The major metabolic pathways involve oxidative degradation via enzymes like CYP3A4/5, CYP2J2, and hydrolysis. The predominant form of Rivaroxaban in plasma is the unchanged drug, with no major active circulating metabolites (Janssen Pharmaceuticals, 2011; Mueck et al., 2011).

Approximately 66% of the drug is excreted in urine (36% unchanged), and 28% is excreted in feces (7% unchanged). Rivaroxaban is primarily excreted via active tubular secretion (approximate 5:1 ratio compared to glomerular filtration). The terminal elimination half-life of Rivaroxaban is 5–9 h in healthy subjects aged 20–45 years. The drug has a low systemic clearance (about 10 L/h) in healthy volunteers following intravenous administration (Janssen Pharmaceuticals, 2011; Mueck et al., 2011). This profile of Xarelto underscores its high bioavailability for lower doses, while its absorption, metabolism, and excretion are influenced by food intake and renal function. In clinical studies, it has been observed that elderly individuals tend to have higher plasma concentrations of Rivaroxaban compared to younger people. This is primarily due to a reduction in total body and renal clearance in the elderly population. Specifically, the mean AUC (area under the curve) values for Rivaroxaban are approximately 50% higher in older adults. Additionally, the terminal elimination half-life of Rivaroxaban is extended in the elderly, ranging from 11 to 13 h, compared to 5–9 h in younger individuals. These changes in pharmacokinetics are important to consider when prescribing Rivaroxaban to elderly patients, as they may be at an increased risk of accumulation of the drug, requiring potential adjustments to dosing (Janssen Pharmaceuticals, 2011; FDA, 2014; Mueck et al., 2011).

Xarelto (Rivaroxaban) should be discontinued at least 24 h before any surgical procedure to reduce the risk of bleeding. This precaution is important because Rivaroxaban is an anticoagulant and stopping it before surgery helps lower the risk of excessive bleeding during the procedure. After the surgery or procedure, Xarelto should be resumed as soon as possible, depending on the patient’s condition and the type of surgery performed. If the patient is unable to take Xarelto by mouth after the procedure, a parenteral anticoagulant (such as heparin) may be administered until oral anticoagulation can be resumed. This ensures continued thromboprophylaxis while avoiding complications related to bleeding (Janssen Pharmaceuticals, 2011; Mueck et al., 2011).

Overdose of Xarelto (Rivaroxaban) can lead to hemorrhage due to its anticoagulant effects. However, systemic exposure does not significantly increase with single doses above 50 mg because of limited absorption at higher doses. In the event of an overdose with bleeding, it is crucial to immediately stop Xarelto and initiate appropriate therapy to manage the bleeding. If the overdose is recent, activated charcoal may be administered to reduce further absorption of the drug from the gastrointestinal tract. Reversal agents such as Andexanet alfa or procoagulant treatments may be considered, depending on the severity of the bleeding and available resources. Close monitoring and supportive care are essential to managing any complications associated with Xarelto overdose (Janssen Pharmaceuticals, 2011; National Center for Biotechnology Information, 2024). The most common side effects of Xarelto (Rivaroxaban) are related to its anticoagulant properties, which increase the risk of bleeding. These include bleeding events, itching (pruritus), pain in the arms or legs, and muscle pain (myalgia). (Janssen Pharmaceuticals, 2011; FDA, 2014).

The liver plays a crucial role in blood coagulation by synthesizing most of the clotting factors and inhibitors of the coagulation pathway (Mestareehi and Abu-Farsakh, 2024). Individuals with hepatic impairment have a reduced ability to synthesize these coagulation factors, which can increase their risk of bleeding due to impaired hemostasis. The severity of hepatic impairment is commonly classified using the Child–Pugh system, with grade A indicating mild impairment, grade B moderate impairment, and grade C severe impairment (Kubitza et al., 2013). Studies have shown that the reduction in clotting factor levels closely correlates with the severity of hepatic impairment as determined by the Child–Pugh score. Additionally, hepatic impairment can influence the pharmacokinetics of drugs metabolized by the liver, potentially leading to increased drug exposure and necessitating dose adjustments for affected patients (Verbeeck, 2008).

Several liquid chromatographic methods have been reported for the quantitation of Rivaroxaban in pharmaceutical dosage forms. One method developed by K. Chandra Sekhar, P. Satya Vani, and Narendra Devanaboyina, focuses on the use of Reverse-Phase High-Performance Liquid Chromatography (RP-HPLC). In this method, separation is achieved on a C18 column (250 × 4.6 mm, 5 µm) at ambient temperature. The mobile phase consists of a mixture of acetonitrile, methanol, and 0.1% phosphoric acid (90:8:2), with the pH adjusted to 4.06. The flow rate is 1.5 mL/min, and absorbance is measured at 234 nm. The linear detection range is from 50 to 200 μg/mL. The method was validated according to ICH guidelines. Rivaroxaban eluted at a retention time of 3.315 min, but early elution may mask the presence of degradants and impurities. Notably, no forced degradation stability studies were conducted, so the impact of degradation products on the chromatographic results was not assessed (Sekhar et al., 2012).

Another method, developed by Mustafa Celebier, Tuba Recber, and Sacide Altinoz, describes a new RP-HPLC method for estimating Rivaroxaban in pharmaceutical dosage forms. This method uses a Luna C18 column (250 mm length, 4.6 mm internal diameter, 5 µm particle size) with a mobile phase consisting of acetonitrile and water (55:45), a flow rate of 1.2 mL/min, and a column temperature of 40°C. Absorbance is performed at 249 nm, and the linear detection range is from 0.005 to 40.0 μg/mL. The method was also validated in accordance with ICH guidelines. Rivaroxaban eluted at a retention time of 3.37 min. However, the very low linear concentration range (from 0.005 to 40.0 μg/mL) used for forced degradation studies is problematic and not recommended for method development. This approach may lead to errors and does not adequately assess the stability of the drug or the presence of degradation products, highlighting the need for a more reliable stability-indicating method. As a result, the absence of a comprehensive stability-indicating method was identified as a significant limitation of this approach (Çelebier TRb et al., 2013). While many studies have focused on the separation and stability of Rivaroxaban raw material, there has been a lack of research on the development of an RP-HPLC method for simultaneous separation and stability-indicating analysis of Rivaroxaban in its pharmaceutical dosage form. To fill this gap, the purpose of the current research is to develop a simple, fast, precise, and accurate RP-HPLC stability-indicating method specifically for Xarelto Tablets^®^. The method will be validated according to ICH (International Council for Harmonisation) and FDA guidelines to meet regulatory standards for quality control and stability testing. This development aims to provide a reliable tool for analyzing the stability of Rivaroxaban in tablet form, ensuring its potency, safety, and efficacy over time.

## Materials and methodology

### Chemicals and reagents

Rivaroxaban USP, CAS: 366789-02-8, Lot No: F10350; hydrochloric acid 12 N, Fisher Scientific,CAS A144-212, Lot No,135238; sodium hydroxide, EM Science, Lot No, 33257-344; Sodium Acetate, Fisher Scientific,CAS S210-500; Acetonitrile (ACN) HPLC Grade, Fisher Scientific,CAS 7778-77; Potassium Phosphate Monobasic, Merck & CO.; Potassium Phosphate Dibasic, ACROSORGANIC CAS7758-11-4, Lot No,A0340250; Potassium Dihydrogen Phosphate, Fluka Chemika, lot no, 290682-490; phosphoric acid (85%) J.T Baker, Lot No, 33397; Glacial acetic acid, Mallinckrodt Inc., CAS 2504-5X6, Lot No., 3532 KBPR; hydrogen peroxide 30%, Fisher Scientific, CAS 7722-84-1, Lot No 094115; hydrogen peroxide 3%, Fisher Scientific, CAS 7722-84-1, Lot No 094115; Deionized Water (in house system); pH Buffer Standards: 4.0, Fischer Scientific, Lot No.,122994; pH Buffer Standards: 7.0, Fischer Scientific, Lot No.,127454; pH Buffer Standards: 9.0, Fischer Scientific, Lot No., 123465.

### Chromatography equipment

The analysis was conducted using Agilent HPLC system, which consisted of the following components:

• 1,100 Series HPLC System with DAD Detector, Agilent Technologies.
  ➢ G1322A Degasser, Serial # JP05033159,
  ➢ G1311A Quaternary Pump Serial # DE111155886,
  ➢ G1329A ALS (Thermostat Auto sampler), Serial # DE11115876,
  ➢ G1316A Column Thermostat, Serial # DE11123026,
  ➢ G1365B DAD (Diode Array Detector), Serial # DE11101219
• 1,100 Series HPLC System with VWD (UV/VIS Detector), Agilent Technologies.
  ➢ G1322A Degasser, Serial # JP05033683,
  ➢ G1311A Quaternary Pump Serial # DE11115504,
  ➢ G1329A ALS (Thermostat Auto sampler), Serial # DE11116580,
  ➢ G1316A COLCOM, Serial #DE14927164,
  ➢ G1314A VWD (Variable Wavelength Detector), Serial # JP92113753
• ChemStation Data Acquisition System for LC, Agilent Technologies.
• Thermo ODS Hypersil C18 (4.6 × 250 mm, 5 µm), Part No; 30105-254630, Serial No; 0153571S.

### Additional equipment

• UV/VIS Spectrophotometer, Hitachi U-2910. Serial No, 2656-003
• Thermo Nicolet IR 200 Spectrometer
• pH Meter, Accumet Research, Fisher Scientific, Serial No AR93311993
• Sonicator/Ultrasonic, T21
• Analytical Balance, Mettler Toledo, Model xp205, s#1129211544
• Lab-line Instrument Inc. Oven (Model: 3512, 800 W, 50/60 Hz, 120 V)
• UV Light, Spectroline, Model ENF-260C, Serial#1358236
• Hot plate/Stirrer, Corning, Model PC-420, Serial No.420505018647
• Disposable Syringe Filter, Nylon Acordis CR 25 mm 0.2 µm, HPLC Certified.
• Filter 0.45 µm PVDF (polyvinylidene difluoride) Membrane.
• pH 0 −13.0 stripes, J.T Baker Inc.,4403-01

### Chromatographic conditions

The chromatographic conditions for the analysis of Rivaroxaban are detailed in Table 1 below. These optimized conditions facilitate efficient separation with a short analysis time, ensuring precise quantification and reliable detection.

**TABLE 1:** Optimized chromatographic conditions for rivaroxaban analysis.

Parameters	Conditions
Column	Thermo ODS Hypersil C18 (4.6 × 250 mm, 5 µm)
Mobile Phase	ACN: BUFFER (25 Mm potassium phosphate buffer monobasic pH 2.9) (30:70)
RT (retention time)	12.1 ± 0.235 min
Flow Rate	1 mL/min
Sample Injector	15 µL loop
Detection Wavelength	249 nm
Column Temperature	Ambient

### Solution preparation procedures

Comprehensive details on sample preparation, mobile phase composition, and analytical procedures employed in this study are provided in the Supplementary Materials under Supplementary Procedures for reproducibility and further reference.

#### Stock solution of Rivaroxaban (10,000 ppm)

Weigh 500 mg of Rivaroxaban and transfer it into a 50 mL volumetric flask. Add 25 mL of ACN: DI water (70:30 v/v) and sonicate for 20 min or until Rivaroxaban is completely dissolved. Complete the volume to the mark with ACN: DI water (70:30 v/v) and shake it thoroughly.

#### Stock solution of Rivaroxaban (1,000 ppm)

Transfer 5.0 mL of stock solution Rivaroxaban (10,000 ppm) into a 50 mL volumetric flask. Complete the volume to the mark with ACN: DI water (70:30 v/v) and shake it thoroughly.

#### Mobile phase B (100% acetonitrile)

Transfer 1,000 mL of ACN into the mobile phase reservoir and sonicate for 20 min to remove air bubbles.

#### Mobile phase A (buffer pH 2.9)

To prepare 1 L of 25 mM potassium phosphate monobasic solution at pH 2.90.

Weigh 3.40 g of Potassium Phosphate Monobasic and transfer it into a 1,000 mL beaker. Add 1,000 mL of DI water and stir until buffer salt is completely dissolved. Place a calibrated pH probe into the solution and adjust the pH by slowly adding phosphoric acid dropwise. Stop once the desired pH of 2.9 is reached. Filter the buffer by using (0.45 µm membrane filter) and sonicate for 20 min to remove any air bubbles.

## Method development and optimization

In HPLC method development, understanding the sample’s hydrophilicity or hydrophobicity is essential, as it guides the choice of stationary and mobile phases and other parameters for optimal separation (Siraj et al., 2024; Mestareehi, 2024). For hydrophobic compounds like Rivaroxaban, reverse-phase HPLC (RP-HPLC) is typically preferred, which uses a nonpolar stationary phase and a polar mobile phase, favoring the retention of hydrophobic analytes (ICH ICoH, 2005). The primary objective of this research was to develop a straightforward, efficient, selective, and precise isocratic RP-HPLC method tailored to determine Rivaroxaban in its raw form. This method also aimed to separate Rivaroxaban from its impurities and any degradation products, eliminating the need for additional separation steps. Several parameters were explored to establish the ideal conditions, focusing on achieving a high theoretical plate number (indicating column efficiency), well-defined peak shapes, minimal peak tailing for raw materials, and effective separation of Rivaroxaban from impurities and degradation products. The following studies were conducted to optimize these aspects, ensuring reliable and reproducible results.

### Solubility studies of Rivaroxaban

The initial step in developing an HPLC method for analyzing Rivaroxaban involved determining its solubility in various solvents to identify an appropriate diluent. Rivaroxaban was tested for solubility in acetonitrile (ACN), methanol, deionized (DI) water, and combinations of these solvents. Approximately 1 mg of Rivaroxaban was dissolved in 3 mL each of ACN, DI water, and methanol individually, but it was found to be insoluble in these solvents alone. However, it was fully soluble in a mixture of 75:25 v/v ACN: DI water. Based on this result, a 70:30 v/v ACN: DI water mixture was chosen as the diluent for preparing Rivaroxaban solutions in further studies, as it provided sufficient solubility while being compatible with the HPLC method. The findings from this solubility testing are summarized in Supplementary Table S1.

### Infrared (IR) study for Rivaroxaban

Infrared (IR) spectroscopy is among the most employed spectroscopic techniques for qualitative analysis, particularly useful for identifying chemical functional groups in various compounds. In the case of Rivaroxaban, IR spectroscopy serves as an effective tool to verify the presence and structure of specific functional groups within its raw material. The IR spectrum provides a “molecular fingerprint” by detecting vibrations in molecular bonds, allowing chemists to confirm the authenticity and purity of the compound. This method is particularly advantageous for confirming the structural integrity of Rivaroxaban and identifying any potential contaminants or impurities through their unique IR absorption patterns (ICH ICoH, 2005).

Weigh about 100 mg of Potassium Bromide (KBr) and 2 mg of Rivaroxaban raw materials. Mix the finely ground solid sample with powdered potassium bromide and press the mixture under high pressure. Potassium Bromide (KBr) under the pressure melts and seals the compound into a matrix. The result is a thin disk. KBr plates must be inserted into a holder to be analyzed by IR-Spectrometer.

**FIGURE 2:**
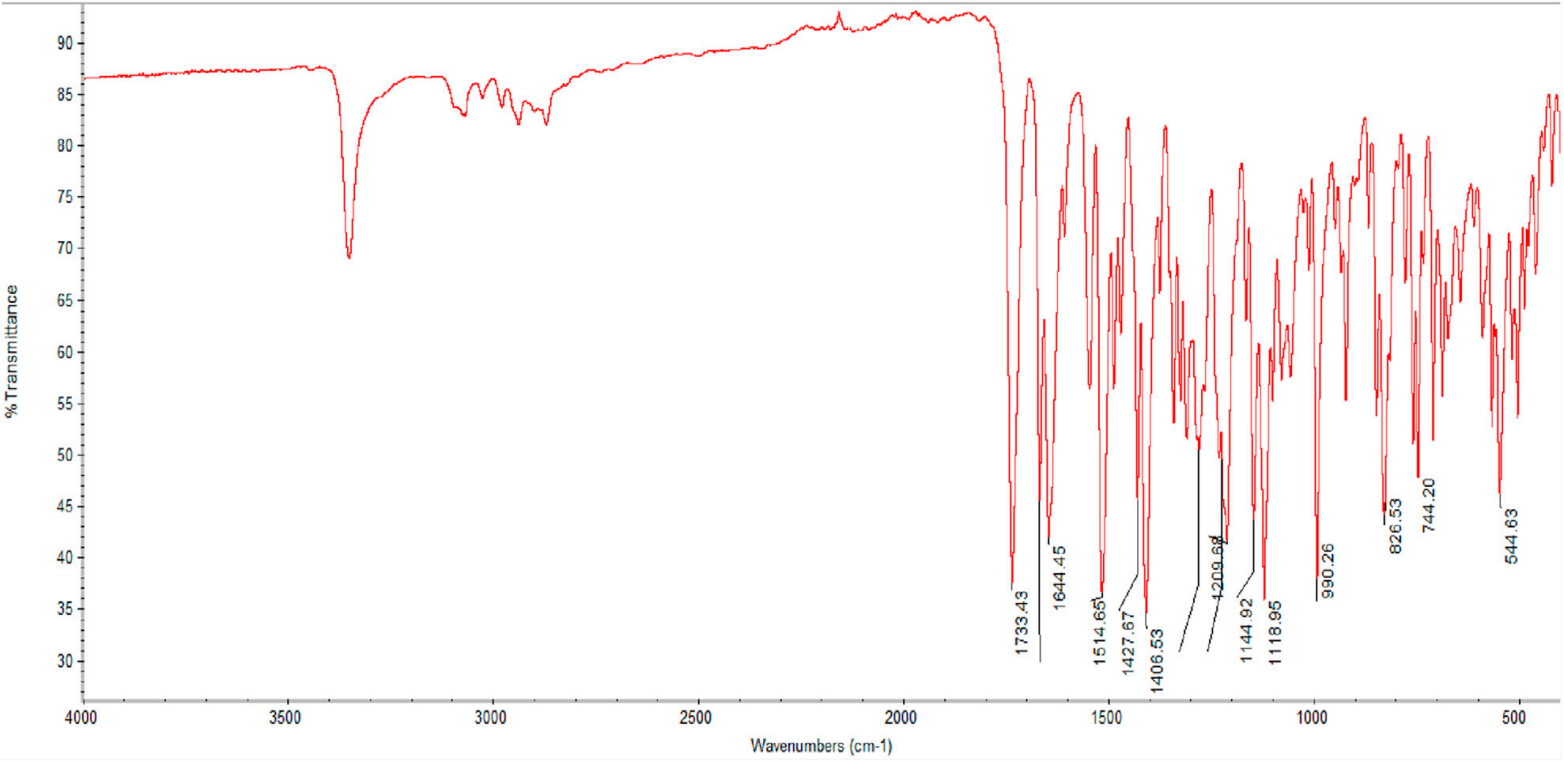
Infrared (IR) spectrum of Rivaroxaban using Thermo Nicolet IR 200 Spectrometer.

The infrared spectrum of Rivaroxaban provides an idea of functional group, from left hand side, the spectrum exhibits at medium absorbing peak at about 3,350 cm^−1^ for N—H stretching frequencies, amides show a very strong band for the C=O group that appears at 1,644.45 cm^−1^. Ester shows a very strong band for the C=O group that appears at 1733.43 cm^−1^. The C=C stretching bands for aromatic rings appear between 1,600 cm^−1^ and 1,400 cm^−1^. Primary chloride absorbs at 544.63 cm^−1^. The infrared (IR) spectrum of Rivaroxaban is illustrated in Figure 2. The chemical functional groups were identified and listed below in Table 2.

**TABLE 2:** IR correlation chart for rivaroxaban.

Frequency (cm^−1^)	Structural unit	Type of vibration	Intensity
3,350	Amide	N—H Stretch	Medium
1,733.43	Ester	C=O Stretch	Strong
1,644.45	Amide	C=O Stretch	Strong
1,514.65	Aromatic	C=C Stretch	Strong
1,500	Amine	Bend	Medium
1,427.64	Methylene	Scissoring	Strong
1,144.92	Ester	C—O Stretch	Strong
544.63	Chloride	C—Cl Stretch	Strong

### Detector wavelength selection

#### Stock solution of Rivaroxaban (20 ppm)

Transfer 1.0 mL of stock solution of Rivaroxaban (1,000 ppm) into a 50 mL volumetric flask. Complete the volume to the mark with ACN: DI water (70:30 v:v) and shake it thoroughly.

To determine the optimal wavelength for Rivaroxaban analysis, 1 mL of a 20 ppm Rivaroxaban stock solution was transferred to a 1 cm quartz cuvette. The solution was scanned over a range of 200–400 nm using a Hitachi UV-VIS double-beam spectrometer as seen in Supplementary Figure S1. The scan revealed maximum absorbance peaks at 200 nm and 249 nm. However, 249 nm was selected as the analytical wavelength because it offered less noise compared to the shorter wavelength of 200 nm, enhancing the accuracy and stability of the measurements. This wavelength choice ensures clearer, more reliable data for quantitative analysis in this study.

### Column selection

The second step in HPLC method development involved selecting the optimal column for Rivaroxaban analysis. Six different C18 columns were initially cleaned and conditioned by flushing them with solvents in the following sequence 50:50 v/v ACN: H_2_O, 75:25 v/v ACN: H_2_O, and 100% ACN. Each solvent mixture was passed through the columns for 30 min at a flow rate of 1 mL/min. This cleaning process helped achieve the appropriate peak shape, tailing, and theoretical plate number for Rivaroxaban. According to the International Council for Harmonisation (ICH) guidelines, acceptable peak shape requires a tailing factor between 0.9 and 2.0. These procedures are in line with standard practices for analytical method validation, as outlined in ICH guideline Q2(R2) on validation of analytical procedures. Additionally, the column should have a theoretical plate number (N) greater than 2,000 to ensure adequate resolution and efficiency for analytical separation (ICH ICoH, 2005). Each HPLC column was utilized twice for different tests. Prior to each use, we implemented rigorous cleaning and equilibration procedures to maintain column integrity and prevent cross-contamination. Details about each tested column are provided in Supplementary Table S2. In this study, 1,000 ppm of Rivaroxaban was injected into the HPLC system to evaluate the performance of six different C18 columns, as illustrated in Supplementary Figure S2. The columns were maintained at ambient temperature using a column thermostat, thereby enhancing the reliability and reproducibility of the analytical results.

All six columns were evaluated under the same chromatographic conditions, including flow rate, injection volume, temperature, and mobile phase, to maintain consistency in the method development process. The results indicated that Column #2 did not meet the ICH acceptance criteria due to a tailing factor below 0.90, which suggested inadequate peak symmetry. Column #3 achieved a tailing factor of 1.30 and a theoretical plate count of 2,516, which met ICH requirements but was lower in performance compared to other columns. Columns 1, 4, 5, and 6 all demonstrated satisfactory numbers of theoretical plates and acceptable tailing factors. Ultimately, Column #6 was selected for further studies, as it provided the most favorable performance. With a tailing factor of 0.94 and 7,890 theoretical plates, Column #6 demonstrated superior efficiency and peak shape relative to the other columns. Detailed information on each column tested is presented in Table 3.

**TABLE 3:** Column selection results.

Column #	Column C18	Retention times (min)	Tailing factor	Number of theoretical plates
1	Phenomenex (4.6 × 150 mm, 5 µm)	9.423	1.50	6,128
2	WATER (4.6 × 150 mm, 5 µm)	9.460	0.78	6,968
3	Agilent Zorbax (4.6 × 250 mm, 5 µm)	7.814	1.30	2,516
4	WATER (4.6 × 250 mm, 5 µm)	11.893	0.976	5,709
5	Phenomenex (4.6 × 250 mm, 5 µm)	15.727	1.219	4,168
6	Thermo Hypersil ODS (4.6 × 250 mm, 5 µm)	11.448	0.940	7,890

### Selection pH of the mobile phase

Controlling pH in a reverse-phase HPLC system is critical to maintaining stable retention times and selectivity, particularly with silica-based columns where free silanol groups on the stationary phase can adsorb basic compounds, leading to unwanted secondary interactions, peak tailing, and poor peak shape. This is especially relevant for Rivaroxaban, a weak base that could interact with the silanol groups through hydrogen bonding, resulting in tailing that interferes with quantification accuracy. Adjusting the pH to 2.9 keeps the silanol groups protonated, reducing their potential interactions with Rivaroxaban, and improving both retention stability and peak shape.

**FIGURE 3:**
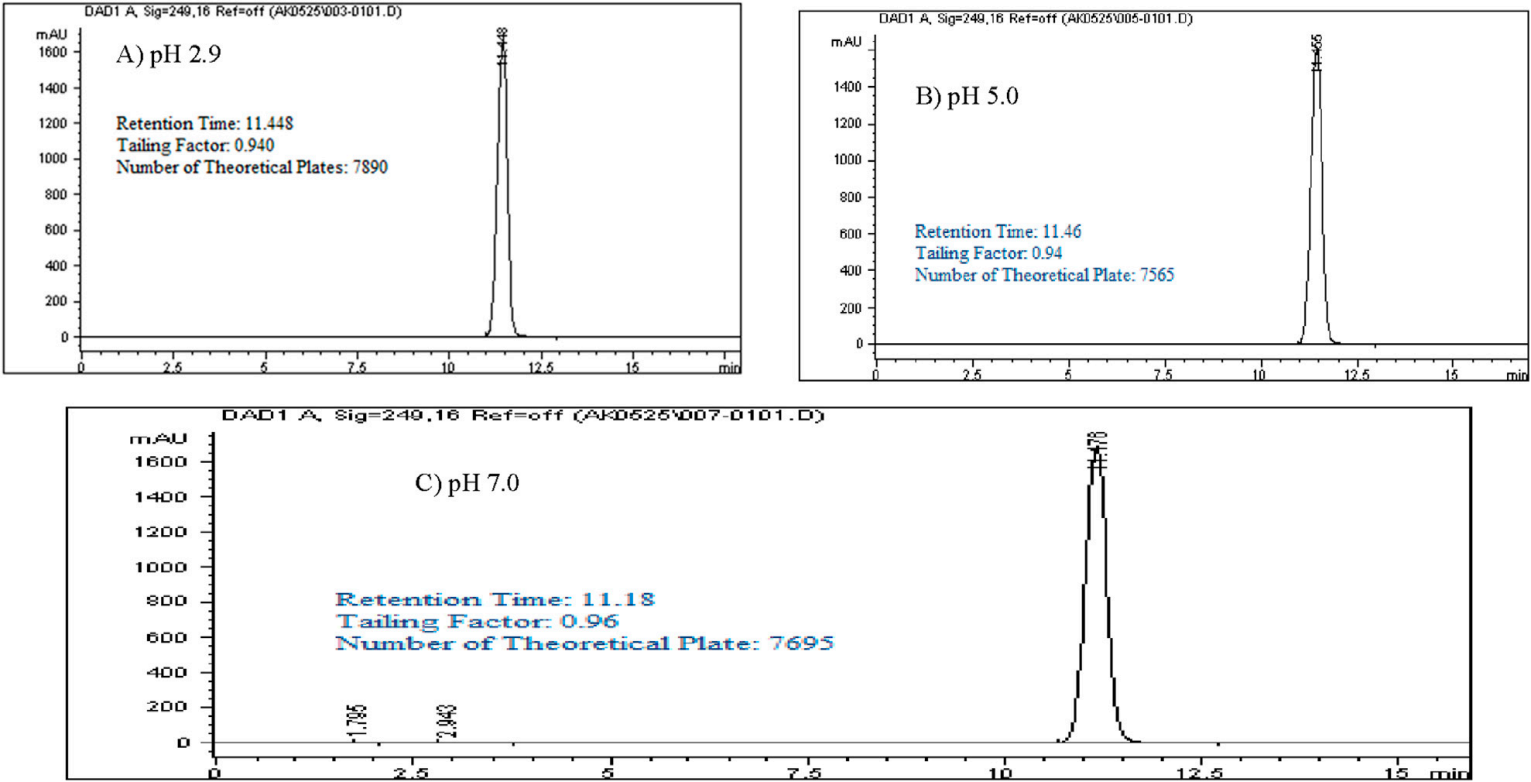
Chromatograms with a mobile phase concentration of 30:70 ACN/buffer pH. **(A)** pH 2.9: 25 mM potassium phosphate buffer monobasic pH 2.9. **(B)** pH 5.0: 25 mM sodium acetate buffer pH 5.0. **(C)** pH 7.0: 25 mM dibasic potassium phosphate buffer pH 7.0. Chromatographic conditions: Isocratic elution, mobile phase 30:70 ACN/buffer, flow rate 1.0 mL/min, detection wavelength at 249 nm, ambient temperature, 15 µL injection volume thermo hypersil ODS C_18_ (4.6 × 250 mm, 5 µm) column.

In this study, a 1,000 ppm Rivaroxaban solution was injected under various pH conditions (2.9, 5.0, and 7.0) using a mobile phase consisting of ACN (phase B) at a 70:30 v/v ratio with the buffer (phase A), as shown in Figure 3. The chromatograms confirmed that pH 2.9 was optimal, minimizing secondary interactions and enhancing chromatographic performance. Supplementary Table S3 summarizes these findings, showing only slight differences in retention time, tailing factor, and theoretical plates across the tested pH levels. However, pH 2.9 provided the highest number of theoretical plates, indicating superior column efficiency and separation, as illustrated in Figure 3. Thus, potassium phosphate monobasic buffer at pH 2.9 was chosen for subsequent method development and validation, achieving an effective balance between retention, peak quality, and chromatographic performance.

### Isocratic elution studies

**FIGURE 4:**
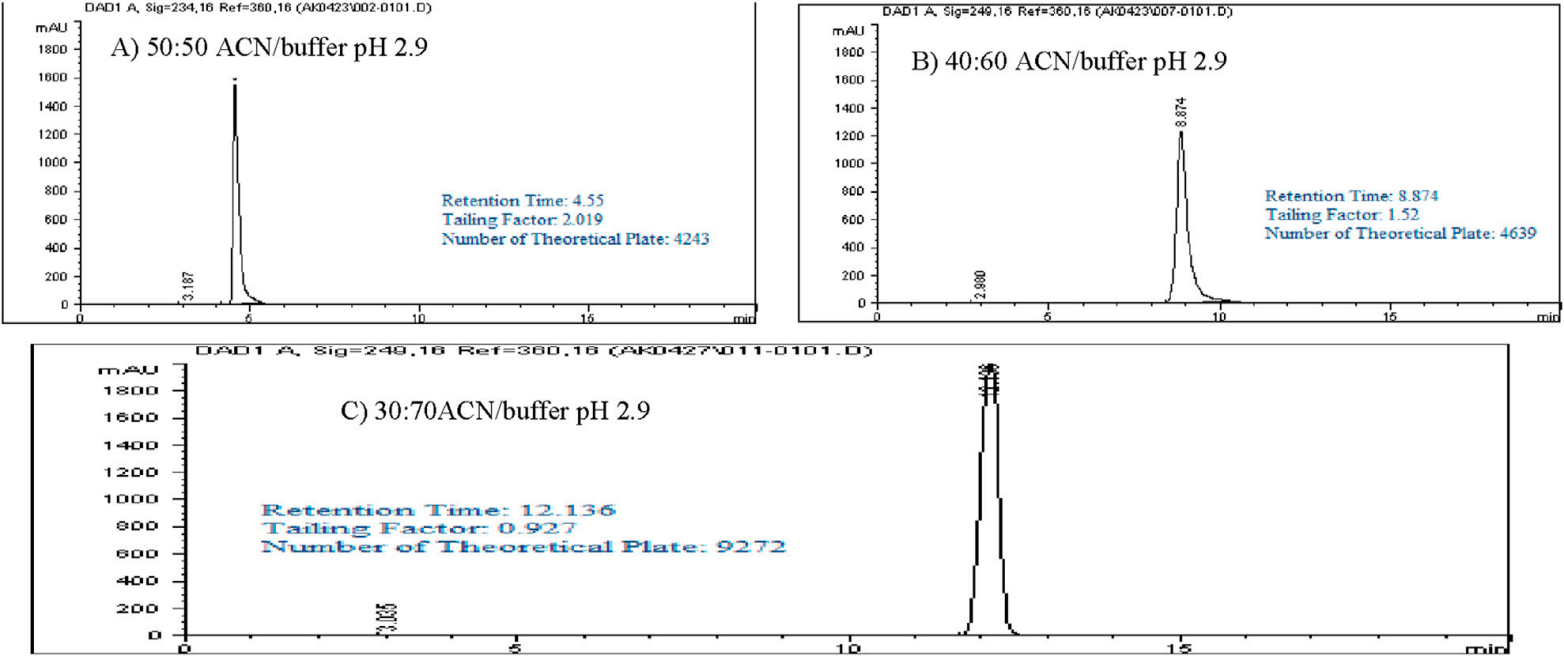
Chromatograms of Rivaroxaban with a different mobile phase concentration of ACN/buffer. **(A)** 50:50 ACN/buffer pH 2.9. **(C)** 30:70 ACN/buffer pH 2.9. **(B)** 40:60 ACN/buffer pH 2.9. Chromatographic conditions: Isocratic elution, mobile phase ACN/25 mM potassium phosphate buffer monobasic pH 2.9, flow rate 1.0 mL/min, detection wavelength at 249 nm, ambient temperature, 15 µL injection volume, thermo hypersil ODS C_18_ (4.6 × 250 mm, 5 µm) column.

Optimizing method validation often involves adjusting the isocratic elution composition. Different ratios of buffer (pH 2.9) and acetonitrile (ACN) were explored to achieve a retention time of approximately 10–13 min, allowing enough time for impurities and degradants to be separated before the main peak. In this study, a 1,000 ppm standard solution of Rivaroxaban was analyzed using various ratios of buffer pH 2.9 (mobile phase A) and ACN (mobile phase B) (v/v). The resulting chromatograms, shown in Figure 4, summarize the retention times, peak areas, and tailing factors, detailed in Supplementary Table S4. When using a 50:50 v/v ratio of buffer pH 2.9 and ACN, Rivaroxaban eluted at 4.55 min, which was too short for effective separation, especially for degradation studies. Further testing with different ACN to buffer ratios ultimately demonstrated that a 70:30 v/v ratio yielded an optimal retention time near 12 min for Rivaroxaban, allowing for more efficient separation of potential impurities and degradants. Therefore, the 70:30 v/v buffer pH 2.9: ACN ratio was selected as the optimal mobile phase composition for further analysis, ensuring improved chromatographic performance. Detailed chromatograms and conditions are presented in Figure 4, with supporting results in Supplementary Table S4.

### Selection of injection volume

Injections of 25 μL and 20 µL of Rivaroxaban led to peak splitting due to saturation of the column’s packing material, resulting in volume overload. This overload effect can compromise peak integrity by causing distortion, impacting the accuracy of quantitative analysis. To prevent these issues, the injection volume was reduced to 15 μL, which allowed for clear, well-defined peaks without overloading the column. This optimized volume was chosen for subsequent method development to ensure consistent, reliable chromatographic results. The chromatograms reflecting these adjustments, along with the chromatographic conditions, are shown in Supplementary Figure S3.

### Nominal concentration selection

To determine the nominal concentration for Rivaroxaban analysis, a calibration curve was established within the linear response range of the detector. Calibration curves are essential in analytical method validation, as they ensure that the detector’s response to various concentrations of the analyte is consistent and proportional. By selecting concentrations within this linear range, the analysis becomes more accurate and reliable, minimizing potential errors due to detector saturation or nonlinearity at higher concentrations.

#### Stock solution of Rivaroxaban (10,000 ppm)

Weigh 500 mg of Rivaroxaban and transfer it into a 50 mL volumetric flask. Add 25 mL of ACN: DI water (70:30 v/v) and sonicate for 20 min or until Rivaroxaban completely dissolved. Complete the volume to the mark with ACN: DI water (70:30 v/v) and shake it thoroughly.

**FIGURE 5:**
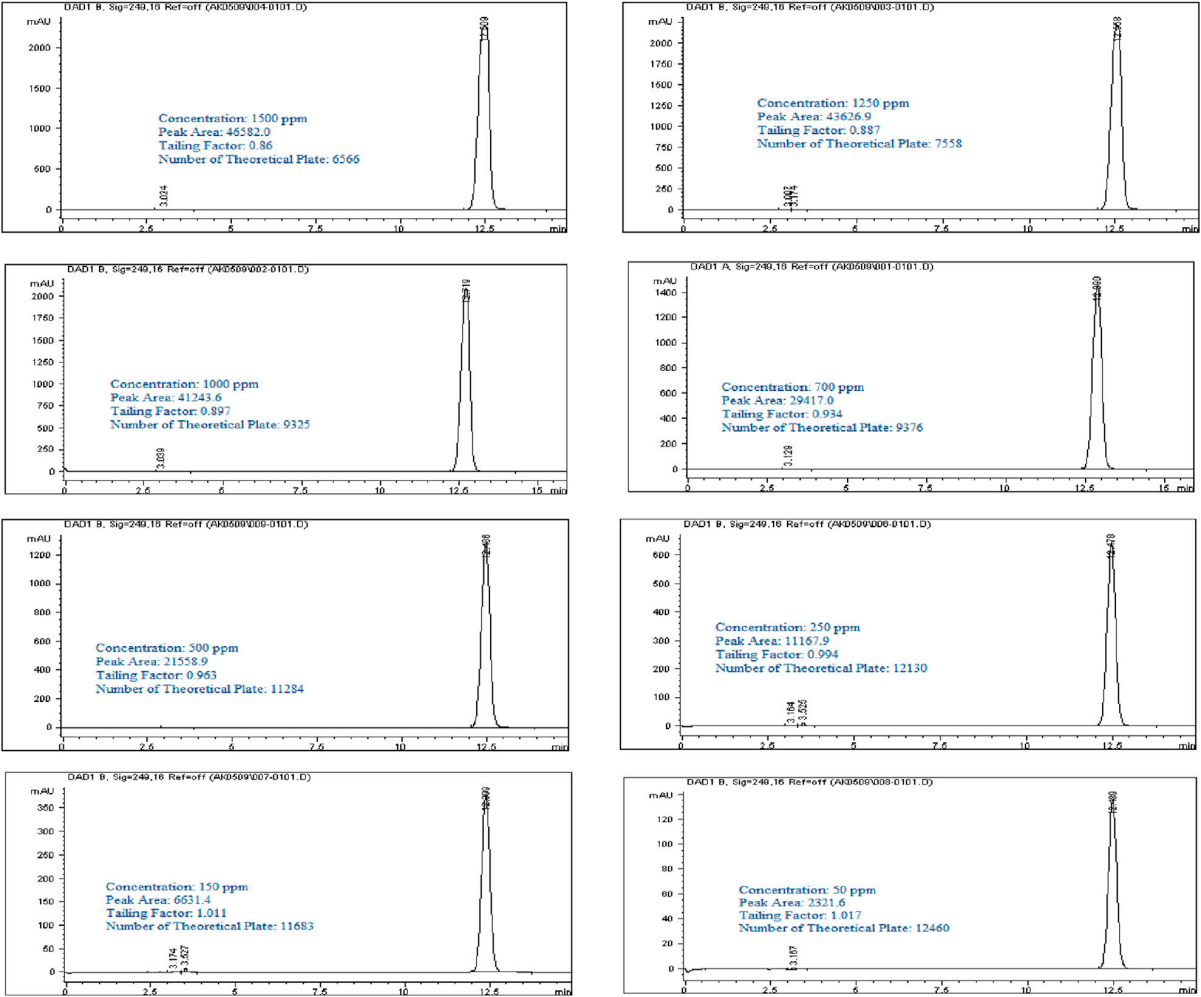
Chromatograms of Rivaroxaban for nominal concentration Selection. Chromatographic conditions: Isocratic elution, mobile phase 30:70 ACN/25 mM potassium phosphate buffer monobasic pH 2.9, flow rate 1.0 mL/min, detection wavelength at 249 nm, ambient temperature, 15 µL injection volume, thermo hypersil ODS C_18_ (4.6 × 250 mm, 5 µm) column.

A stock solution of Rivaroxaban at a concentration of 10,000 ppm was used to prepare a series of diluted solutions with concentrations of 1,500 ppm, 1,250 ppm, 1,000 ppm, 700 ppm, 500 ppm, 250 ppm, 150 ppm, and 50 ppm (shown in Supplementary Materials). These serial solutions were analyzed by injecting them into the HPLC system under the optimized isocratic elution conditions. The chromatograms, chromatographic conditions, and peak areas corresponding to each concentration can be viewed in Figure 5, with the peak area results summarized in Table 4. This approach allows for accurate calibration and validation across a range of concentrations, ensuring reliable quantitation in further analysis.

**TABLE 4:** Summary of nominal concentration selection.

Sample #	Concentration (ppm)	Peak area (mAu)
1	1,500	46,582
2	1,250	43,626.9
3	1,000	41,244
4	700	29,417
5	500	21,558.9
6	250	11,168
7	150	6,631.4
8	50	2,321.6

**FIGURE 6:**
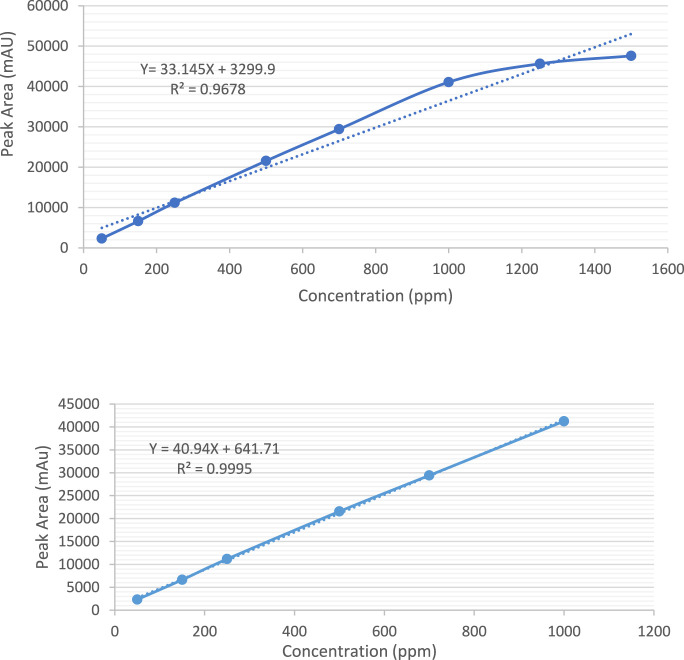
**(A)** Calibration curve for the quantification of Rivaroxaban across a concentration range of 50 ppm–1,500 ppm. **(B)** Calibration curve for the quantification of Rivaroxaban across a concentration range of 50 ppm–1,000 ppm.

It was observed that the concentrations of 1,500 ppm and 1,250 ppm fell outside the linear response range of the detector, as shown in Figure 6A. Therefore, the linearity test confirmed a linear response within the concentration range of 50–1,000 ppm. A calibration curve for Rivaroxaban was created by plotting peak area against concentration, yielding a high linear correlation coefficient of 0.9995, as depicted in Figure 6B. To determine the nominal concentration, a concentration at 70% of the highest value within the linear range was selected. Consequently, 700 ppm was chosen as the nominal concentration and will serve as the reference concentration for further method development studies. This ensures accurate and consistent results within the linear response range of the detector, optimizing the method’s reliability and precision.

### Mixed degradation study (acid, base, and oxidation)

**FIGURE 7:**
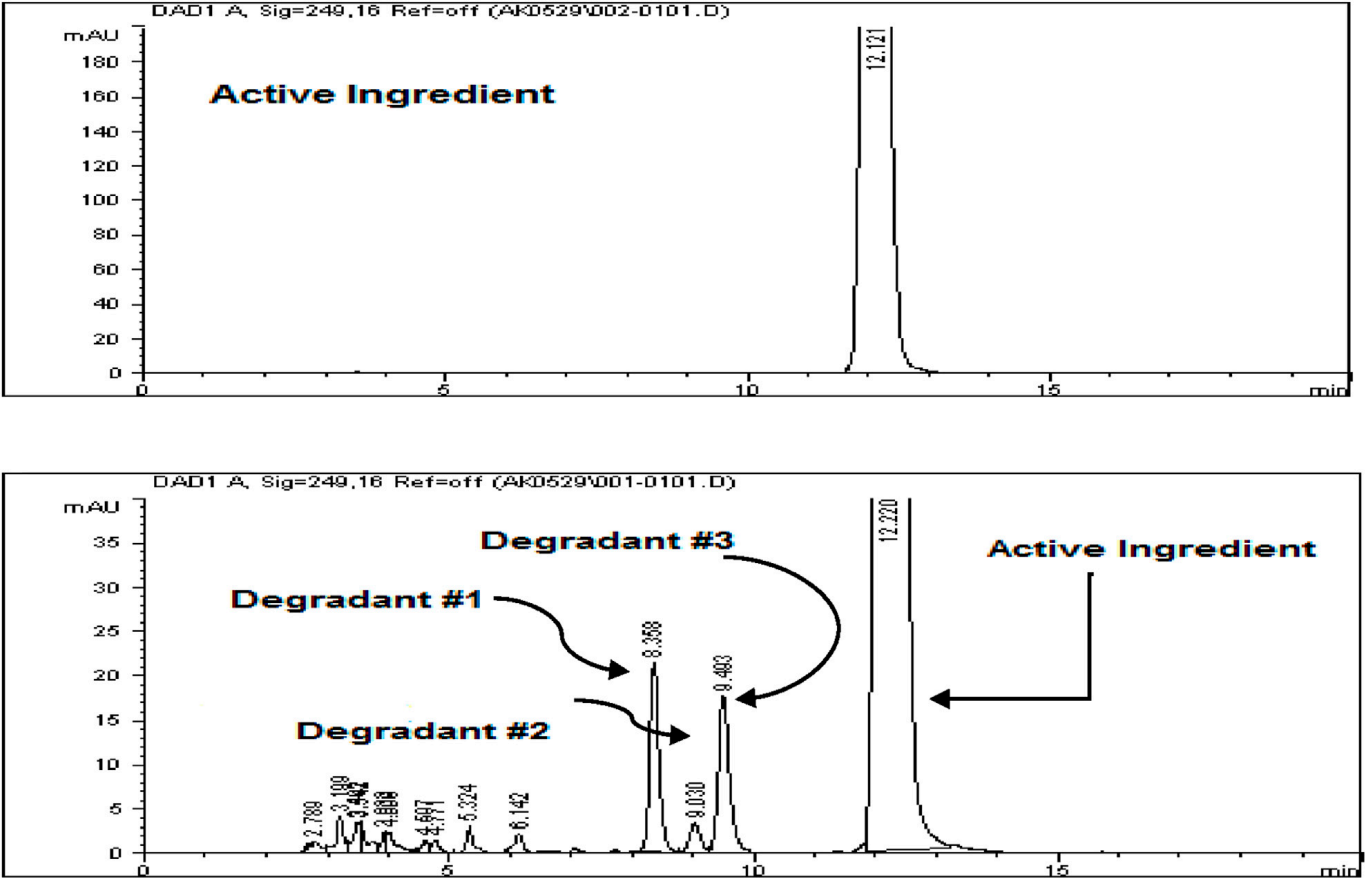
**(A)** Chromatograms of Rivaroxaban 700 ppm control solution used for degradation study. **(B)** Zoomed in chromatogram of the optimized mixed degradation study. Chromatographic conditions: Isocratic elution, mobile phase 30:70 ACN/25 mM potassium phosphate buffer monobasic pH 2.9, flow rate 1.0 mL/min, detection wavelength at 249 nm, ambient temperature, 15 µL injection volume, thermo hypersil ODS C_18_ (4.6 × 250 mm, 5 µm) column.

The objective of the mixed degradation study was to assess the capability of the developed HPLC method to effectively separate Rivaroxaban from all potential degradants and impurities, ensuring accurate quantification and purity assessment (ICH ICoH, 2005; United States pharmacopeia X, 1990). For this study, a control sample of 700 ppm was prepared from a 3,500 ppm stock solution of Rivaroxaban and injected into the HPLC system to establish a baseline for comparison. This control sample chromatogram, shown in Figure 7A, allows for calculating the percent degradation of Rivaroxaban after mixed degradation conditions are applied, confirming that the method can distinguish between the active ingredient and any degradative by products.

Transfer 1 mL of each the stress degraded solutions that produced less than 10% degradation (0.01 N HCl for 24 h, 0.01 N NaOH for 1 h, and 0.05% H_2_O_2_ for 24 h), into a test tube. Mix the solution thoroughly and filter with (0.45 µm membrane filter) before injected into the HPLC system. The chromatogram of Rivaroxaban from mixture degradation study is shown in Figure 7B.

The study showed that Rivaroxaban’s primary degradants eluted at retention times of 8.3, 9.0, and 9.4 min. These peaks were completely separated from the main Rivaroxaban peak, which eluted at approximately 12 min, confirming the method’s capacity to distinctly resolve the active ingredient from its degradation products. This separation ensures that the developed HPLC method is suitable for accurately identifying and quantifying Rivaroxaban without interference from degradants. The detailed degradation results for each study condition are summarized in Table 5.

**TABLE 5:** Mixed degradation study results under optimized chromatographic conditions.

Stress condition	Exposed time	Temperature (°C)^a^	Color	Peak area	% degradation
None	None	None	Clear	29,894.4	----
0.01 N HCl	24 h	75	Clear	26,868.3	8.80
0.01 N NaOH	One hour	75	Clear	27,074.50	8.10
0.05% H_2_O_2_	24 h	75	Clear	28,837.0	6.50
Mixture solution	-	-	Clear	27,144.12	9.20

^a^ Heat it on a heating block at 75°C for the specified duration.

## Method validation

To ensure compliance with Good Laboratory Practice (GLP) and Good Manufacturing Practice (GMP), the developed analytical method must meet the validation requirements outlined by several authoritative guidelines, such as the ICH (International Conference on Harmonization) guidelines Q2A and Q2B (ICH ICoH, 2005), the FDA (U.S. Food and Drug Administration) (FDA, 2004), and the USP (U.S. Pharmacopeia) (United States pharmacopeia X, 1990). The following key validation parameters were evaluated to confirm the method’s reliability and accuracy:

• System Suitability Test (SST): This verifies that the HPLC system meets the desired analytical standards before sample analysis, ensuring consistent performance.
• Specificity: Ensures that the method can clearly distinguish Rivaroxaban from impurities and degradants, demonstrating it is free from interference.
• Method Robustness: Assesses the method’s reliability under varied conditions, such as slight changes in pH or solvent composition.
• Solution Stability: Confirms that the Rivaroxaban solution remains stable over the testing period without degradation.
• Linearity and Range: Establishes that the method produces accurate results over a specified concentration range.
• Method Accuracy and Precision: Includes:
  ○ Method Precision: Reproducibility of results within the same method over time.
  ○ Injection Precision: Consistency of injection volumes and peak areas.
  ○ Intermediate Precision: Method reliability across different days and analysts.
• Limit of Detection (LOD) and Limit of Quantitation (LOQ): Defines the minimum concentration at which impurities or degradants can be reliably detected and quantified. This comprehensive validation, following ICH and FDA guidelines, ensures the method’s suitability for high-quality, reproducible analysis of Rivaroxaban.

## System suitability

System suitability test (SST) is a vital component of HPLC analysis as recommended by the ICH Q2R1 guidelines. It ensures that the HPLC system and related components such as equipment, software, electronics, and samples are operating correctly and consistently. System suitability tests help establish that the method’s performance is reliable before analyzing samples, ensuring high data integrity and accuracy. The key SST parameters and acceptance criteria for HPLC, as specified by the guidelines, are (ICH ICoH, 2005):

• Relative standard deviation (RSD) of response of replicate injections NMT 1%
• Relative standard deviation (RSD) of retention time of replicate injections NMT 1%
• Number of theoretical plates MT 2000
• Tailing factor between 0.9 and 2
• Capacity factor greater than 2
• Resolution values greater than 2.0
• % Drift ≤ 2

These criteria collectively confirm that the HPLC system is suitable for analysis and can reliably deliver accurate and reproducible results. By meeting these benchmarks, laboratories can ensure the precision, accuracy, and consistency of their analyses as per regulatory standards.

### Standard solutions preparation for system suitability

#### Stock standard solution of Rivaroxaban (10,000 ppm)

Weigh 500 mg of Rivaroxaban and transfer it into a 50 mL volumetric flask. Add 25 mL of ACN: DI water (70:30 v/v) and sonicate for 20 min or until Rivaroxaban is completely dissolved. Complete the volume to the mark with ACN: DI water (70:30 v/v) and shake it thoroughly.

#### Working standard solution of Rivaroxaban (700 ppm) #1

Transfer 3.5 mL of stock solution Rivaroxaban (10,000 ppm) into a 50 mL volumetric flask. Complete the volume to the mark with ACN: DI water (70:30 v/v) and shake it thoroughly.

#### Working standard solution of Rivaroxaban (700 ppm) #2

Transfer 3.5 mL of stock solution Rivaroxaban (10,000 ppm) into a 50 mL volumetric flask. Complete the volume to the mark with ACN: DI water (70:30 v/v) and shake it thoroughly.

**FIGURE 8:**
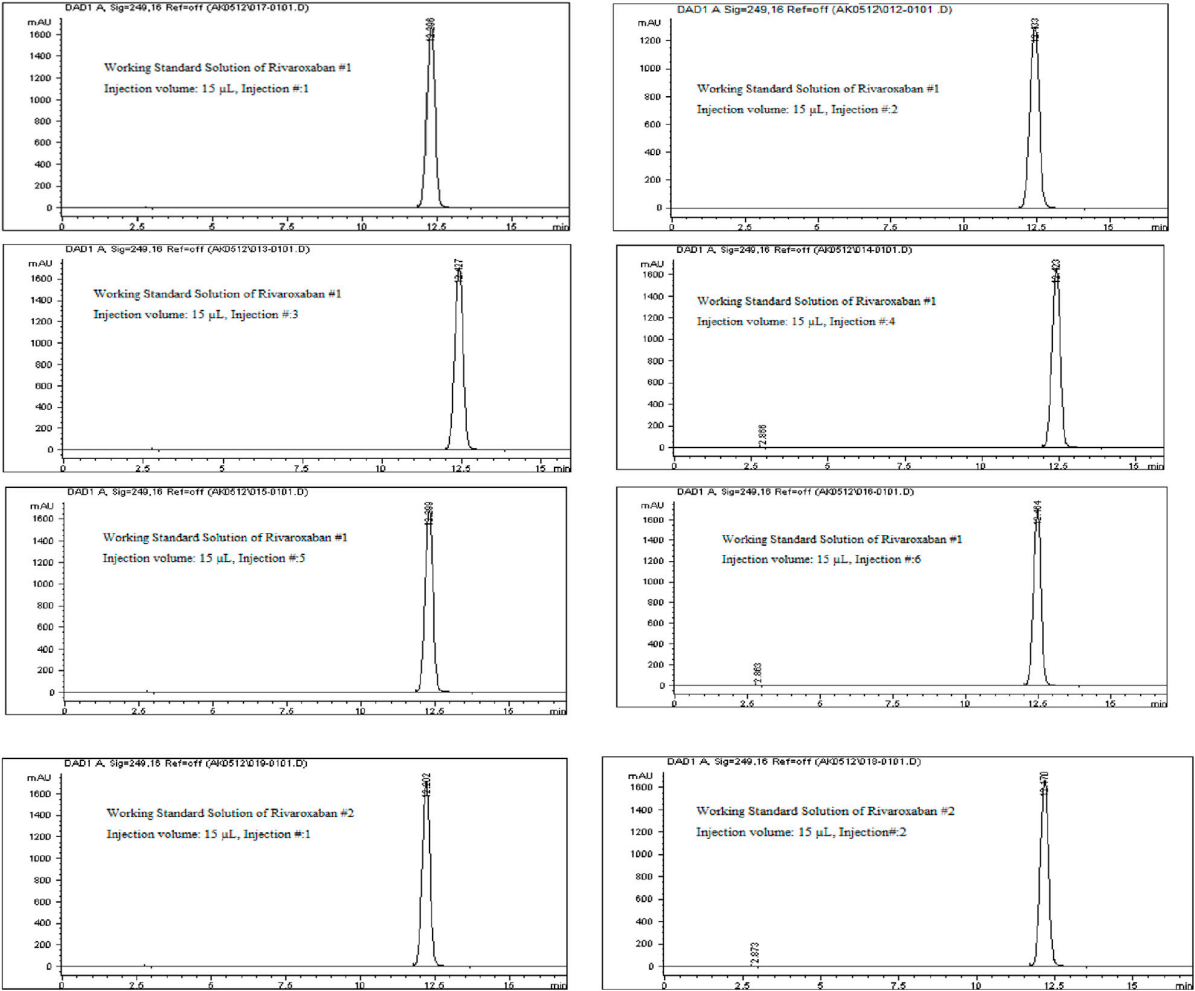
Chromatograms of the working standard solution for Rivaroxaban from System Suitability Study #1 and System Suitability Study #2. Chromatographic conditions: Isocratic elution, mobile phase 30:70 ACN/25 mM potassium phosphate buffer monobasic pH 2.9, flow rate 1.0 mL/min, detection wavelength at 249 nm, ambient temperature, 15 µL injection volume, thermo hypersil ODS C_18_ (4.6 × 250 mm, 5 µm) column.

System suitability tests were performed by injecting the working standard solutions of Rivaroxaban (labeled as #1 and #2) to confirm the reliability and consistency of the HPLC system. The working standard solution #1 was injected six times consecutively, as illustrated in Figure 8, while solution #2 was injected twice, as shown in Figure 8. The results from these injections were used to calculate key system suitability parameters, including the %RSD of peak area and retention time for Rivaroxaban #1 (six injections) and Rivaroxaban #2 (two injections). This metric ensures consistent peak responses and retention times. Tailing Factor and Number of Theoretical Plates were calculated for each peak to assess peak shape and column efficiency.

The percentage drift (% Drift) was calculated by using equation below:

$$\%\text{ Drift}=\frac{\text{As}-\text{Ac}}{\text{As}}\;x\;100$$

where **A**_**S**_ is the average peak area of six replicate injection of working standard solution #1 and **A**_**C**_ is the average peak area of two replicate injections of working standard solution #2.

The percent relative standard deviations were calculated by using equation below.

$$\%\text{ RSD}=\frac{S}{Xave}x100$$

where %RSD is the relative standard deviation, *S* is the standard deviation, *Xave* is the average value of measurements.

$$S=\sqrt{\frac{1}{n-1}\sum_{i=1}^{N}\left(x_{i}-\bar{x}\right)}$$

where S is the standard deviation, *x*_*i*_ is the value of measurement *i*, *x* is the average value of measurements and N is the number of measurements.

### Specificity

Specificity measures the ability of a method to accurately identify and quantify the target analyte, Rivaroxaban, in the presence of degradants, impurities, and other related compounds within the sample matrix. The specificity of an analytical method ensures that it effectively isolates the analyte peak from other components without interference. Acceptance Criteria for Specificity are The peak purity factor should exceed 990, indicating a pure peak without co-elution, Peaks must be well-separated, with a resolution greater than 2 to confirm baseline separation, and No interference from impurities, degradants, or related substances should affect the peak of interest, Rivaroxaban (ICH ICoH, 2005). This study aims to confirm that the method is specifically measuring the Rivaroxaban peak with accurate concentration, clearly separated from any degradants or impurities.

Mixed degradation samples were prepared by combining 1 mL each from acid, base, and oxidative degradation solutions with degradation levels under 10%: 0.01 N HCl (8.8% degradation over 24 h), 0.01 N NaOH (8.1% degradation for 1 hour), and 0.05% H_2_O_2_ (6.5% degradation over 24 h). The solutions were combined, thoroughly mixed, and filtered through a 0.45 µm membrane filter. The sample was then injected into an Agilent 1100 HPLC system equipped with a Diode Array Detector (DAD).

### Method Robustness

The robustness of an analytical method tests its ability to remain consistent and unaffected by minor, deliberate changes in parameters. This is a key part of method validation under ICH guidelines, as it helps ensure that the method performs reliably under varied conditions in practical applications (ICH ICoH, 2005). In this study, robustness was assessed by adjusting five critical parameters:

⁃ Buffer pH: (2.9 ± 0.2)
⁃ Flow Rate: (1.0 ± 0.2 mL/min)
⁃ Wavelength: (249 ± 2 nm)
⁃ % B Composition: (30 ± 5% B)
⁃ Volume injected: (15 ± 2 μ L)

The acceptance criteria for robustness are listed below (ICH ICoH, 2005):

• Tailing factor between 0.9 and 2.0
• Number of Theoretical plates ≥ 2,000
• All peaks should be separated from the peak of interest by RS > 2.0

#### Mobile phase A (buffer pH 2.9)

To prepare 1 L of 25 mM potassium phosphate monobasic solution at pH 2.90.

Weigh 3.40 g of potassium phosphate monobasic and transfer it into a 1,000 mL beaker. Add 1,000 mL of DI water and stir until buffer salt is completely dissolved. Place a calibrated pH probe into the solution and adjust the pH by slowly adding phosphoric acid dropwise. Stop once the desired pH of 2.9 is reached. Filter the buffer by using (0.45 µm membrane filter) and sonicate for 20 min to remove any air bubbles.

For further analysis, additional mobile phases with buffer pH adjustments were prepared at pH 3.1 and pH 2.7, following the same method previously established. Each prepared mobile phase was introduced individually into the HPLC system to assess their impact on the retention and peak shape of the analyte. This allowed for comparison of chromatographic performance at slightly different pH values to ensure robustness and fine-tune separation parameters. This step helps confirm that the method provides consistent results across slight variations in buffer pH, which is essential for method validation.

Transfer 1 mL of each of the stress degraded solutions that produced less than 10% degradation (0.01 N HCl for 24 h, 0.01 N NaOH for 1 h and 0.05% H_2_O_2_ for 24 h) into a test tube. Mix the solution thoroughly and filter with (0.45 µm membrane filter) before injecting into the Agilent 1100 HPLC system with Diode Array Detector (DAD).

### Solution stability

The purpose of solution stability testing is to assess how the analyte of interest, in this case Rivaroxaban, responds over time to various environmental factors such as temperature, light, and humidity (Administration FaD, 2003). By examining these influences, solution stability ensures accurate and reliable quantification of the analyte in routine analysis, which is crucial for method validation.

Solution stability was evaluated by measuring the percent change in the peak area over time, comparing the initial (immediate) injection with subsequent injections at 24, 48, and 72 h. This method helps to detect any degradation or instability in the analyte under different storage or testing conditions. The % change in peak area was calculated using the following formula:

$$\%\text{ Change in area}=\frac{At-Ai}{Ai}x100$$

A_t_ is a Peak Area at different time *t* and A_i_ is a Peak Area at initial time *i*.

No new peaks or lost peaks should appear in the chromatographic profile between the first and last injections, indicating no significant degradation or instability in the solution over the course of the analysis (ICH ICoH, 2005).

### Linearity and range for active ingredient (Rivaroxaban)

**FIGURE 9:**
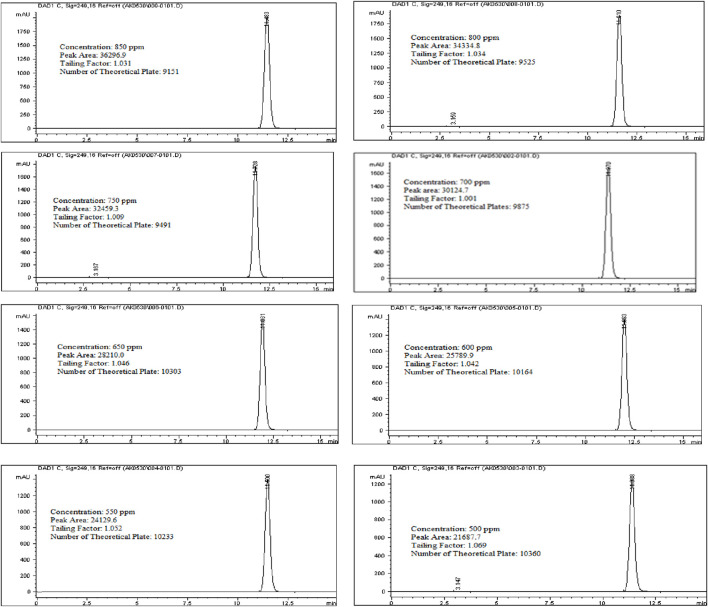
Chromatograms for linearity and range study for Rivaroxaban active ingredient. Chromatographic conditions: Isocratic elution, mobile phase 30:70 ACN/25 mM potassium phosphate buffer monobasic pH 2.9, flow rate 1.0 mL/min, detection wavelength at 249 nm, ambient temperature, 15 µL injection volume, thermo hypersil ODS C_18_ (4.6 × 250 mm, 5 µm) column.

To assess the linearity of Rivaroxaban, a stock solution of 5,000 ppm was used to prepare a serial dilution series with concentrations of 850 ppm, 800 ppm, 750 ppm, 700 ppm, 650 ppm, 600 ppm, 550 ppm, and 500 ppm. Each solution was injected into the HPLC system under the established optimum conditions, and their chromatograms, along with relevant chromatographic parameters, are shown in Figure 9. The corresponding peak area results are detailed in Table 6.

**TABLE 6:** Limit of detection study results.

Sample #	Concentration (ppm)	Peak area	Retention time	Signal to noise ratio
1	10.0	415.81	11.54	117.2
2	5.0	185.54	11.90	41.0
3	2.0	107.47	11.47	31.7
4	1.5	69.40	11.49	18.1
5	1.3	60.13	11.62	16.2
6	1.0	51.91	11.79	10.80
7	0.90	40.63	12.0	7.50
8	0.50	25.17	11.75	6.20
9	0.40	17.58	11.76	5.70
10	0.30	12.85	11.85	4.0
11	0.20	9.91	11.83	2.6

For the linearity, the correlation coefficient for Rivaroxaban must be at least 0.999 to meet acceptance criteria, ensuring that the relationship between concentration and detector response is linear within the tested range. This high threshold confirms the method’s reliability for quantitative analysis across the concentration range.

### Stock solution of Rivaroxaban (5,000 ppm)

Weigh 250 mg of Rivaroxaban and transfer it into a 50 mL volumetric flask. Add 25 mL of ACN: DI water (70:30 v/v) and sonicate for 20 min until Rivaroxaban is completely dissolved. Complete the volume to the mark with ACN: DI water (70:30 v/v) and shake it thoroughly.

A stock solution of Rivaroxaban with a concentration of 5,000 ppm was used to prepare a series of diluted solutions at concentrations of 850 ppm, 800 ppm, 750 ppm, 700 ppm, 650 ppm, 600 ppm, 550 ppm, and 500 ppm (shown in Supplementary Materials). These solutions were analyzed using HPLC under optimized isocratic elution conditions. The resulting chromatograms, chromatographic parameters, and corresponding peak areas for each concentration are presented in Figure 9, with a summary of peak area results provided in Table 7.

**TABLE 7:** Linearity results for rivaroxaban active ingredient.

Sample #	Concentration (ppm)	Peak area (mAu)
1	850	36,296.9
2	800	34,334.8
3	750	32,459.3
4	700	30,124.7
5	650	28,210
6	600	25,789.9
7	550	24,130.9
8	500	21,687.7

### Accuracy

The accuracy of the developed HPLC method was evaluated to assess how closely the measured values align with the true or theoretical values. To determine accuracy, Rivaroxaban solutions were prepared in triplicate at 80%, 100%, and 120% of the nominal concentration (700 ppm) for the active ingredient. The acceptance criteria for method accuracy were defined as follows: For the active ingredient, the percent recovery should be between 95% and 105% of the theoretical value. These criteria ensure that the method can reliably and accurately quantify the active ingredient, as well as any impurities and degradants, across a range of concentrations.

**FIGURE 10:**
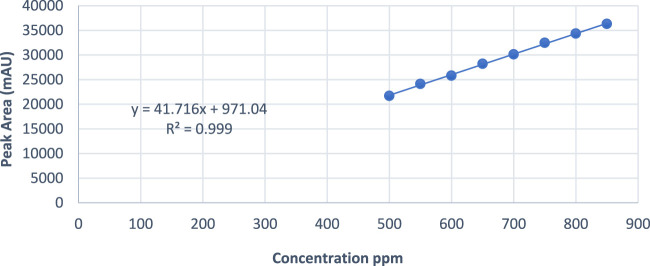
Peak area vs. concentration plot of Rivaroxaban for linearity study.

To assess the accuracy of the developed method for the active ingredient, a stock solution of Rivaroxaban (5,000 ppm) was diluted to prepare samples with concentrations of 850 ppm, 700 ppm, and 500 ppm. These samples were then analyzed by injecting them into the HPLC system, and the corresponding peak areas were recorded. The percentage recovery of the active ingredient was calculated using the linear regression equation derived from the calibration curve, Y = 41.716X + 971.04, as shown in Figure 10. Here: y represents the peak area of the injected sample, and x corresponds to the concentration of Rivaroxaban in ppm. Using this equation, the calculated concentration could be compared to the theoretical concentration to determine the percent recovery for each sample. These results are summarized in Table 8, which shows that the method meets the accuracy criteria by achieving recoveries within the acceptable range of 95%–105%.

$$Percentage\;Recovery=\frac{Csample}{Cstandard}x100$$

**TABLE 8:** Limit of quantitation study results.

Sample #	Concentration (ppm)	Peak area	Retention time	Signal to noise ratio
1	1.0	50.44	11.58	11.8
2	1.0	51.55	11.68	11.0
3	1.0	52.44	11.70	11.7
4	1.0	49.97	11.70	10.7
5	1.0	52.18	11.68	12.1
6	1.0	52.03	11.63	11.2
7	1.0	51.56	11.58	12.2
8	1.0	51.16	11.50	10.6
9	1.0	52.13	11.53	10.8
10	1.0	51.73	11.55	10.5
Average		51.52		11.26
Standard deviation		0.793		0.64
%RSD		1.54		5.68

*Csample*: Measured concentration of the sample obtained from linear regression equation.

*Cstandard*: Actual concentration of the prepared sample.

Triplicate samples at each concentration level (850 ppm, 700 ppm, and 500 ppm) were injected into the HPLC system to ensure precision and accuracy in the results. The corresponding chromatograms for these injections, along with detailed chromatographic conditions, are presented in Supplementary Figures S10–S12. These figures illustrate the method’s reproducibility and the stability of the results across multiple injections, confirming that the developed method meets the required accuracy and reliability standards for quantifying Rivaroxaban at various concentration levels.

### Method precision

The precision of an analytical method reflects the consistency and reliability of the measurements taken under the same conditions, defined by how closely multiple measurements of the same concentration match each other. According to the International Council for Harmonisation (ICH) guidelines, precision is categorized into three main levels (ICH ICoH, 2005):

1. Repeatability: This refers to the closeness of results when the same analyst performs multiple injections or measurements of the same sample, using the same instrument and method over a short period of time.
2. Intermediate Precision: Also known as within-laboratory precision, it assesses variations within the same lab but includes different conditions, such as different days, analysts, or instruments.
3. Reproducibility: This measures the precision between laboratories, indicating how well the method performs across different labs.

Each level of precision provides important insights into the method’s robustness and consistency in diverse settings, ensuring that results are reliable and reproducible.

### Repeatability (method precision)

Repeatability evaluates the method’s reliability by determining the variability that may arise from sample preparation errors by the same analyst under identical operating conditions. This level of precision helps assess the method’s sensitivity to minor operational changes, ensuring consistent and reliable results. The acceptance criteria for method precision are stringent, as per regulatory standards. Specifically, the % Relative Standard Deviation (%RSD) of the peak area for the compound of interest should not exceed 1% (NMT 1%).

To assess repeatability for Rivaroxaban’s active ingredients, six separate samples of a 700 ppm concentration were prepared and injected into the HPLC system using the method’s optimized chromatographic conditions, as illustrated in Supplementary Figure S13. The % Relative Standard Deviation (% RSD) of peak areas for these injections was then calculated to confirm the consistency and reliability of the method. Meeting the repeatability criteria, as outlined in the acceptance guidelines, demonstrates that the method consistently yields precise measurements for the target compound under the same experimental conditions.

#### Stock solution of Rivaroxaban (700 ppm)

Weigh 35 mg of Rivaroxaban and transfer it into a 50 mL volumetric flask. Add 25 mL of ACN: DI water (70:30 v/v) and sonicate for 20 min or until Rivaroxaban is completely dissolved. Complete the volume to the mark with ACN: DI water (70:30 v/v) and shake it thoroughly.

### Injection precision

Injection precision assesses the consistency of the method regarding instrument-related errors, including potential variability from the column, injector, detector, and integration device during sample injection. According to the acceptance criteria, the % RSD for the peak area of Rivaroxaban should not exceed 1%. To demonstrate injection precision for the active ingredient, a single Rivaroxaban sample at 700 ppm was prepared and injected six times into the HPLC system under optimized conditions. As shown in Supplementary Figure S14, the % RSD of the peak areas was calculated and found to meet the required acceptance criteria, confirming the method’s reliability in terms of injection precision.

#### Stock solution of Rivaroxaban (700 ppm)

Weigh 35 mg of Rivaroxaban and transfer it into a 50 mL volumetric flask. Add 25 mL of ACN: DI water (70:30 v/v) and sonicate for 20 min or until Rivaroxaban is completely dissolved. Complete the volume to the mark with ACN: DI water (70:30 v/v) and shake it thoroughly.

A single Rivaroxaban sample at a concentration of 700 ppm was prepared and injected six times into the HPLC system under optimized chromatographic conditions. The peak areas were measured, and the % RSD for injection precision was calculated. The results, shown in Supplementary Table S7, met the acceptance criteria for injection precision, confirming that the method is consistent and precise for the Rivaroxaban active ingredient.

### Intermediate precision (robustness)

Intermediate precision for the Rivaroxaban analytical method was assessed by evaluating the consistency of results across variations in conditions, such as different analysts, dates, instruments, and columns. According to the acceptance criteria for intermediate precision, the % RSD of peak area for Rivaroxaban raw material should not exceed 1.5%. To determine the reproducibility of the method under varying conditions, six samples of 700 ppm Rivaroxaban were prepared. These samples were injected into different HPLC systems, with different columns, under the same developed method but using different equipment and on different dates. The chromatograms and conditions for the analysis are presented in Supplementary Figure S15, and the results for the peak areas are detailed in Supplementary Table S8.

#### Stock solution of Rivaroxaban (5,000 ppm)

Weigh 250 mg of Rivaroxaban and transfer it into a 50 mL volumetric flask. Add 25 mL of ACN: DI water (70:30 v/v) and sonicate for 20 min or until Rivaroxaban is completely dissolved. Complete the volume to the mark with ACN: DI water (70:30 v/v) and shake it thoroughly.

#### Stock solution of Rivaroxaban (700 ppm)

Transfer 7.0 mL of stock solution Rivaroxaban (5000 ppm) into a 50 mL volumetric flask. Complete the volume to the mark with ACN: DI water (70:30 v/v) and shake it thoroughly.

Intermediate precision parameters for the developed method are as follows:

• HPLC: 1100 Series HPLC system with MWD (UV/VIS Detector), Agilent Technologies
• Separation Mode: Isocratic (Reversed-Phase Separation)
• Column: Water XTERRA RP-18 (4.6 × 250 mm, 5 µm)
• Mobile Phase: Solvent A: 25 mM potassium phosphate monobasic buffer 2.9
Solvent B: 100% ACN
• Solvent Strength: (70:30 v/v) Buffer: ACN
• Absorbance: 249 nm
• Flow Rate: 1.0 mL/min
• Injection Volume: 15 µL
• Column Temperature: Ambient
• Run Time: 18 min

### Limit of detection (LOD)

The limit of detection (LOD) was evaluated to determine the minimum concentration of analyte in a sample that can be reliably detected, though not necessarily quantified precisely. This was done using Rivaroxaban as a surrogate for impurities and degradants. LOD was determined based on the signal-to-noise ratio, with the acceptance criterion being a signal-to-noise ratio of ≥3.

#### Stock solution of Rivaroxaban (5,000 ppm)

Weigh 250 mg of Rivaroxaban and transfer it into a 50 mL volumetric flask. Add 25 mL of ACN: DI water (70:30 v/v) and sonicate for 20 min or until Rivaroxaban is completely dissolved. Complete the volume to the mark with ACN: DI water (70:30 v/v) and shake it thoroughly.

#### Stock solution of Rivaroxaban (10 ppm)

Transfer 0.1 mL of stock solution Rivaroxaban (5000 ppm) into a 50 mL volumetric flask. Complete the volume to the mark with ACN: DI water (70:30 v/v) and shake it thoroughly.

### Limit of quantitation (LOQ)

**FIGURE 11:**
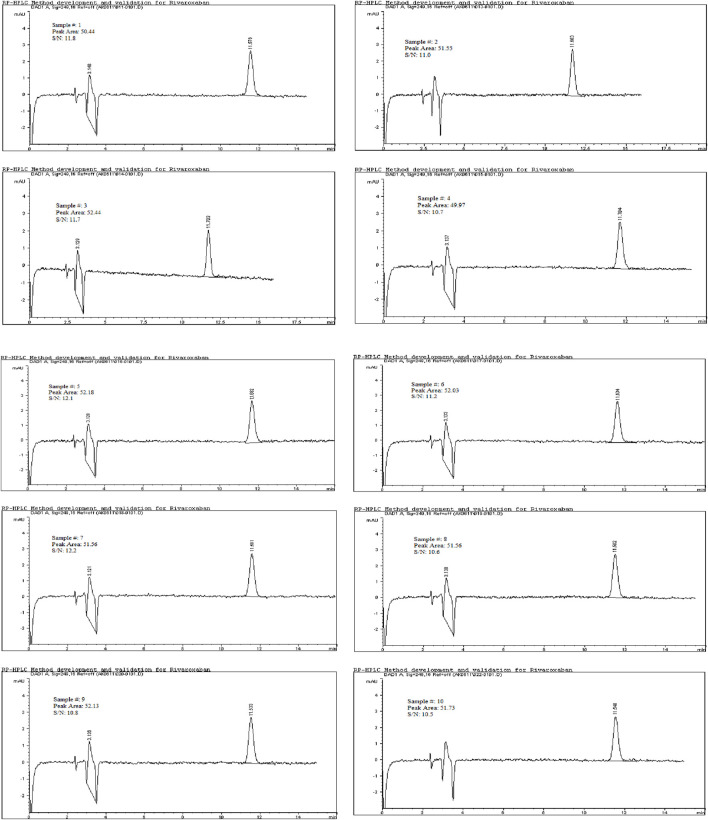
Chromatograms for limit of quantitation study for 1.0 ppm. Chromatographic conditions: Isocratic elution, mobile phase 30:70 ACN/25 mM potassium phosphate buffer monobasic pH 2.9, flow rate 1.0 mL/min, detection wavelength at 249 nm, ambient temperature, 15 µL injection volume, thermo hypersil ODS C_18_ (4.6 × 250 mm, 5 µm) column.

To determine the limit of quantitation (LOQ) for Rivaroxaban, a series of dilutions were prepared, and the signal-to-noise ratios were calculated based on a 5,000 ppm stock solution. The LOQ was initially estimated at 1.0 ppm, as indicated by the results in Table 8, where the signal-to-noise ratio for this concentration met the acceptance criterion of ≥10 and % RSD ≤10 . To confirm the LOQ, a 1.0 ppm Rivaroxaban solution was prepared and injected ten times into the HPLC system. The resulting chromatograms, shown in Figure 11, provided further data on the signal-to-noise ratio and allowed for the determination of precision. The results from these injections are summarized in Table 8, where the signal-to-noise ratio was found to meet the acceptance criteria. Additionally, the % RSD for this concentration was ≤10%, confirming that 1.0 ppm is the LOQ for the developed method.

## Results and discussion

Rivaroxaban is an oral anticoagulant that directly inhibits factor Xa, thereby preventing clot formation. It is commonly prescribed to reduce the risk of stroke in patients with non-valvular atrial fibrillation and to prevent deep vein thrombosis (DVT) following hip or knee replacement surgeries (Alam et al., 2023; Mestareehi et al., 2021). Several RP-HPLC methods have been developed for quantifying rivaroxaban in pharmaceutical forms. One method utilized a Phenomenex Luna C18 column (250 × 4.6 mm, 5 µm) at 40°C, with an acetonitrile-water (55:45 v/v) mobile phase, a 1.2 mL/min flow rate, and detection at 249 nm. Rivaroxaban eluted at 3.37 min, and the method was validated per ICH guidelines (Sekhar et al., 2012). Another study employed a HiQSil C18 column (250 × 4.6 mm, 5 µm) at room temperature, using a methanol-water (65:35 v/v) mobile phase, a 1.4 mL/min flow rate, and detection at 249 nm. The retention time was 3.12 min, and the method was validated for accuracy and precision (Çelebier TRb et al., 2013). Notably, many existing methods lack comprehensive stability-indicating analyses, underscoring the need for methods that assess rivaroxaban’s stability in tablet formulations to ensure potency, safety, and efficacy.

The results of the system suitability test, as outlined in Table 9, indicate that all peaks achieved complete resolution with a tailing factor close to 1, demonstrating optimal peak shape. The number of theoretical plates exceeded 9,000, which reflects high column efficiency and satisfactory separation. The %RSD values for peak area and retention time of six replicate injections from working standard solution #1 were 0.33% and 0.59%, respectively. For working standard solution #2, the %RSD values for peak area and retention time from two replicate injections were 0.43% and 0.19%, respectively. These low %RSD values demonstrate the system’s repeatability and precision. Additionally, the percent drift was below 1%, indicating stability and minimal variability between working standard solutions. These results meet the acceptance criteria for system suitability according to ICH guidelines, confirming that the system performs reliably. This test was conducted prior to analyzing other parameters to ensure system readiness for accurate data generation in subsequent studies.

**TABLE 9:** System suitability results for working standard solution for rivaroxaban# 1 and rivaroxaban #2.

Standard #1 injection	Retention time	Peak area (mAU)	Plate count	Tailing factor
1	12.296	31,205.2	9,959	0.945
2	12.433	31,043.9	10,489	0.943
3	12.427	31,211.1	10,657	0.943
4	12.423	31,094.6	10,167	0.954
5	12.299	31,022.4	10,197	0.951
6	12.464	30,954.3	10,599	0.950
Average	12.39	31,042.35		
STDEV	0.073	102.45		
Retention Time % RSD	0.59			
Peak Area % RSD		0.33		
% Drift	−0.22			
Standard # 2 injection				
1	12.202	31,014.6	10,521	0.958
2	12.170	31,256.4	9,537	0.957
Average	12.19	31,109.90		
STDEV	0.02	134.77		
Retention Time % RSD	0.19			
Peak Area % RSD		0.43		

**FIGURE 12:**
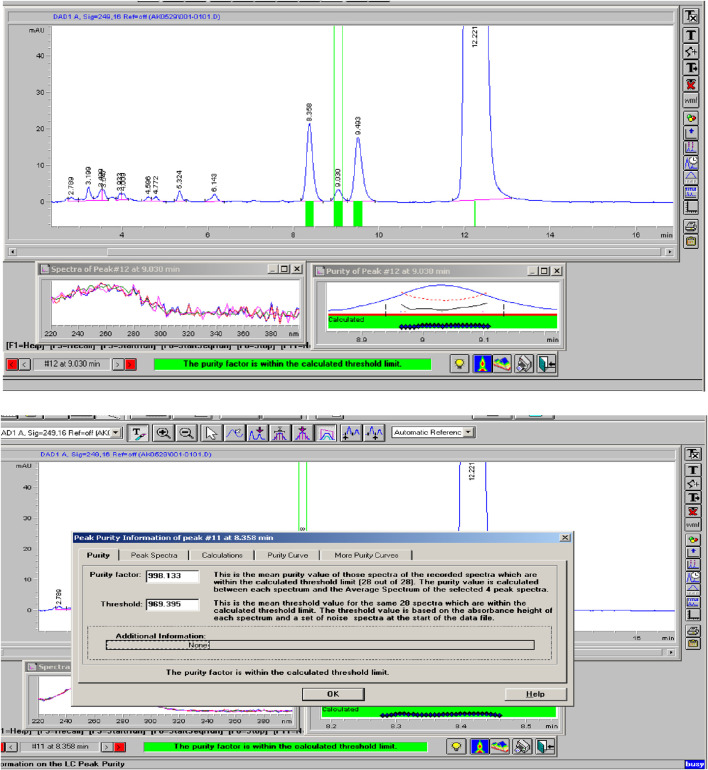
Peak purity of Rivaroxaban degraded to 9.2%. Chromatographic conditions: Isocratic elution, mobile phase 30:70 ACN/25 mM potassium phosphate buffer monobasic pH 2.9, flow rate 1.0 mL/min, detection wavelength at 249 nm, ambient temperature, 15 µL injection volume, thermo hypersil ODS C_18_ (4.6 × 250 mm, 5 µm) column.

The Rivaroxaban peak was observed at a retention time of 12.20 min, with three additional major peaks from degradation products at 8.358, 9.030, and 9.493 min. All peaks were well-resolved from the Rivaroxaban peak, meeting the specificity requirement for resolution. Moreover, the peak purity factor exceeded the threshold, confirming that the main Rivaroxaban peak was free from interference. The specificity study demonstrated that the method accurately isolates and quantifies Rivaroxaban in the presence of degradants and impurities. Chromatographic evidence, including a purity chromatogram and a 3D image of Rivaroxaban, is provided in Figure 12 and Supplementary Figure S4, respectively, showcasing the method’s efficacy in meeting ICH and FDA guidelines (ICH ICoH, 2005).

The robustness study results are summarized in Table 10. All results adhered to ICH guidelines. The tailing factor ranged between 0.9 and 2, and the number of theoretical plates exceeded 2,000. The method demonstrated robustness against minor variations in solvent strength, flow rate, buffer pH, wavelength, and injection volume. Chromatograms from the robustness study and corresponding chromatographic conditions are shown in Supplementary Figures S5–S9.

**TABLE 10:** Method robustness results variations for mixed degradation sample.

Parameters	Conditions	Retention time (min.)	Tailing factor	Peak area (mAU)	Number of theoretical plates
pH of buffer	2.7	11.3	1.05	30,365	12,566
	2.9	12.2	1.04	31,341	14,771
	3.1	11.95	1.05	30,855	14,135
Flow rate mL/min	0.8	14.16	1.04	35,135	16,018
	1	12.2	1.05	31,341	14,771
	1.2	10.2	1.05	25,578	13,922
Wavelength	247	12.29	1.05	30,627	14,959
	249	12.2	1.05	31,341	14,771
	251	12.08	1.06	31,187	14,433
% B composition	25	17.67	0.98	29,665.0	16,990
	30	12.2	1.05	31,341	14,771
	35	8.23	1.13	27,703	10,774
Injection volume ( μ L)	13	12.2	1.06	27,899	15,601
	15	12.2	1.05	31,341	14,771
	17	12.38	1.03	34,776	13,973

**FIGURE 13:**
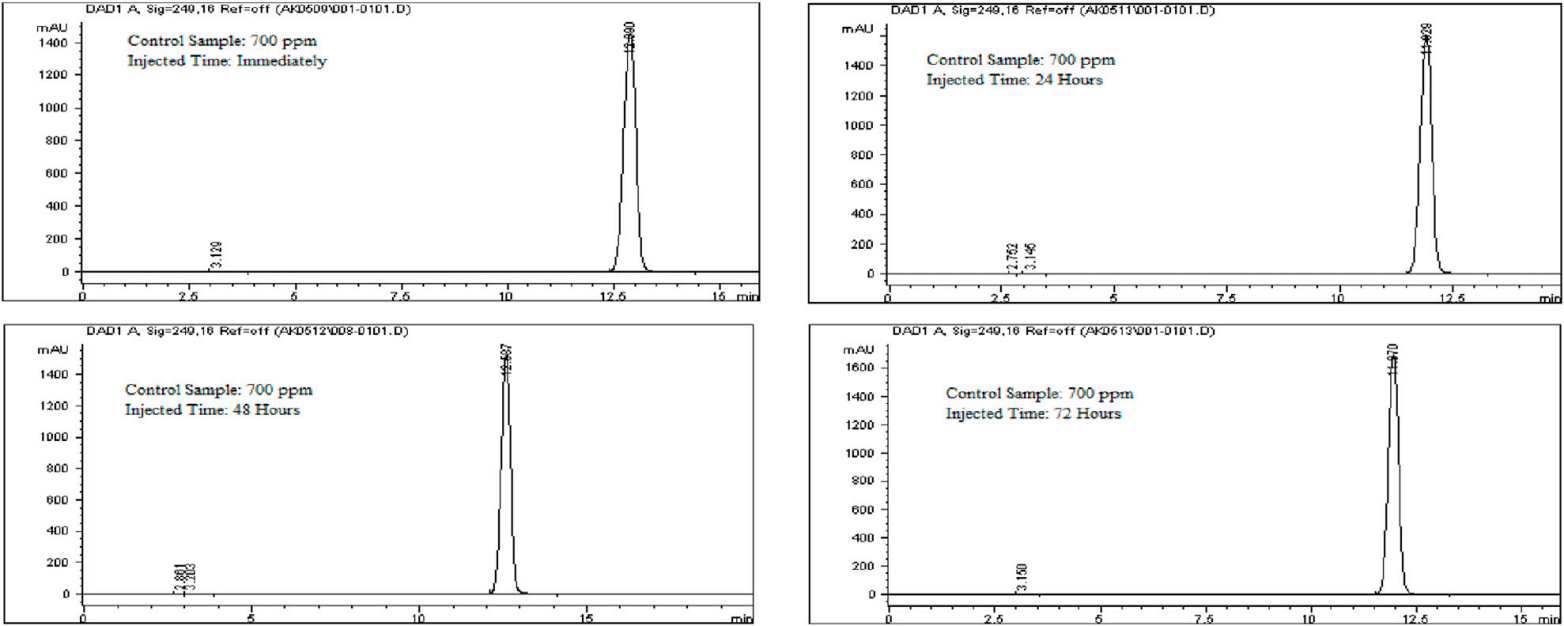
Chromatograms for solution stability study for Rivaroxaban 700 ppm. Chromatographic conditions: Isocratic elution, mobile phase 30:70 ACN/25 mM potassium phosphate buffer monobasic pH 2.9, flow rate 1.0 mL/min, detection wavelength at 249 nm, ambient temperature, 15 µL injection volume, thermo hypersil ODS C_18_ (4.6 × 250 mm, 5 µm) column.

To evaluate solution stability, a 700 ppm Rivaroxaban solution was freshly prepared from a 10,000 ppm stock solution and immediately injected into the HPLC system. The same 700 ppm solution was reinjected at 24, 48, and 72 h. Chromatograms and chromatographic conditions are displayed in Figure 13. No new peaks or peak loss was observed in the chromatographic profiles between the first and last injections, indicating no significant degradation or instability of the solution during the analysis as shown in Supplementary Table S5.

Eight concentrations of Rivaroxaban standard solutions (850 ppm, 800 ppm, 750 ppm, 700 ppm, 650 ppm, 600 ppm, 550 ppm, and 500 ppm) were prepared to assess linearity, with each concentration injected individually into the HPLC system. The recorded peak areas for each concentration are presented in Table 7. A calibration curve was constructed by plotting peak areas against concentrations. The resulting linear regression equation demonstrated a correlation coefficient of 0.9990, as shown in Figure 10. This value meets the acceptance criterion for linearity, confirming the HPLC method’s reliability and linear response across the tested concentration range. These findings validate the method’s suitability for quantitative analysis of the active ingredient.

Each sample of Rivaroxaban (850 ppm, 700 ppm, and 500 ppm) was prepared in triplicate, injected into the HPLC system, and analyzed for peak area to calculate percent recovery. The accuracy study results, summarized in Table 11, indicate that all tested concentrations of the active ingredient met the acceptance criteria. These results confirm the method’s accuracy for determining Rivaroxaban, with percent recovery within the specified range, validating its reliability for quantifying the active ingredient in raw material.

**TABLE 11:** Accuracy results for rivaroxaban active ingredient.

Concentration (ppm)	Peak area	Percent recovery	Average percent recovery	% RSD
500	21,723.1	99.6	99.7	0.32
	21,803.0	100.0		
	21,671.1	99.4		
700	29,970.3	99.4	99.4	0.21
	30,033.1	99.6		
	29,909.8	99.2		
850	36,278.4	99.7	100.5	0.74
	36,623.0	101.1		
	36,798.9	100.6		

To evaluate the repeatability of the analytical method for rivaroxaban, six individual samples at a concentration of 700 ppm were prepared and injected into the HPLC system under optimized chromatographic conditions, as depicted in Supplementary Figure S13. The percent relative standard deviation (%RSD) of the peak areas from these injections was calculated to assess the method’s precision. According to ICH guidelines, a %RSD not more than 2.0% is typically acceptable for repeatability in analytical methods. Compliance with this criterion demonstrates that the method consistently provides precise measurements for rivaroxaban under the same experimental conditions.

Six individual samples of Rivaroxaban (700 ppm) were prepared and analyzed using the HPLC system under optimized chromatographic conditions. The peak areas for each injection were recorded, and the % RSD was calculated to evaluate the method’s precision. As presented in Supplementary Table S6, the % RSD values met the acceptance criteria for injection precision, confirming the method’s reliability in consistently measuring peak areas across repeated injections. Additionally, a single Rivaroxaban sample (700 ppm) was injected six times into the HPLC system under identical conditions to further assess injection precision. The % RSD was determined, and as shown in Supplementary Table S7, the results complied with the acceptance criteria, reinforcing the method’s precision for quantifying the active ingredient. Moreover, to assess intermediate precision, six separate Rivaroxaban samples (700 ppm) were prepared and injected into a different HPLC system. The % RSD of the peak areas was calculated, and the results, detailed in Supplementary Table S8, met the acceptance criteria. These findings confirm that the method consistently produces reliable results under varying laboratory conditions, as specified in the validation protocol.

**FIGURE 14:**
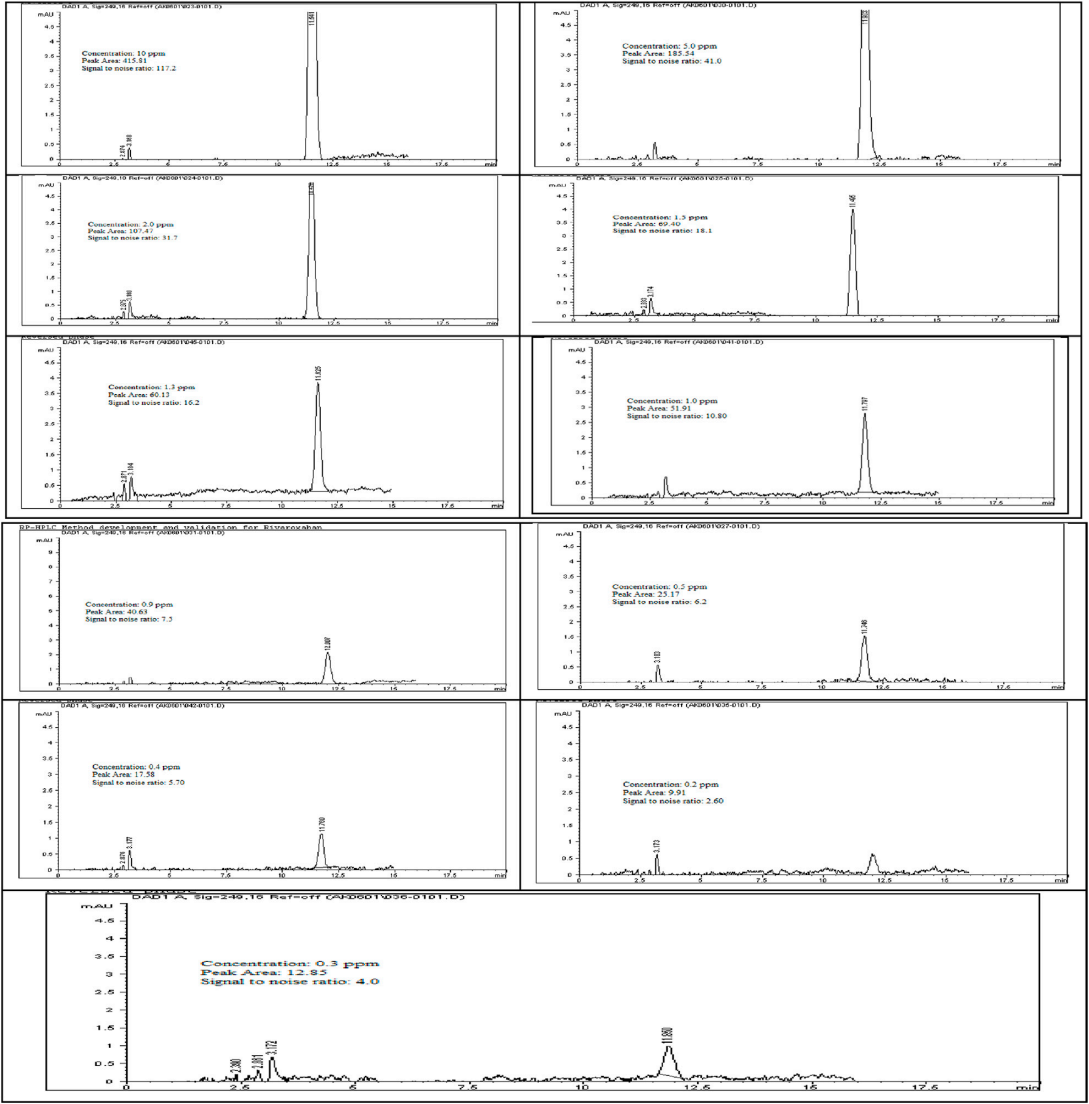
Chromatograms for limit of detection (LOD) of Rivaroxaban. Chromatographic conditions: Isocratic elution, mobile phase 30:70 ACN/25 mM potassium phosphate buffer monobasic pH 2.9, flow rate 1.0 mL/min, detection wavelength at 249 nm, ambient temperature, 15 µL injection volume, thermo hypersil ODS C_18_ (4.6 × 250 mm, 5 µm) column.

The limit of detection (LOD) for Rivaroxaban was determined by preparing serial dilutions from a 5,000 ppm stock solution, with concentrations ranging from 10.0 ppm, 5.0 ppm, 2.0 ppm, 1.5 ppm, 1.0 ppm, 0.90 ppm, 0.50 ppm, 0.40 ppm, 0.30 ppm, and 0.2 ppm. Each solution was injected into the HPLC system, and the resulting chromatograms (shown in Figure 14) were analyzed to calculate the signal-to-noise ratios. The results, presented in Table 6, indicate that the 0.3 ppm concentration yielded a signal-to-noise ratio of 4.0. Based on this finding, 0.3 ppm was selected as the LOD for the developed method, meeting the acceptance criterion of a signal-to-noise ratio of ≥3. Additionally, to assess the limit of quantitation (LOQ) for Rivaroxaban, a 1 ppm solution was prepared and injected ten times into the HPLC system. The resulting chromatograms were analyzed to evaluate the peak areas, and the signal-to-noise ratios were calculated. The % RSD (relative standard deviation) of the peak areas was determined to be 1.54%, which is well within the acceptance criteria for the LOQ, where the % RSD should be ≤ 10%. These results confirm that the 1.0 ppm concentration meets the criteria for LOQ, indicating that the developed method is precise enough for quantification at this low concentration.

To confirm our results for the Limit of Detection (LOD) and Limit of Quantitation (LOQ), the International Council for Harmonisation (ICH) recommends various approaches, depending on the instrument used for analysis, the nature of the analyte, and the suitability of the method. One acceptable approach is the standard deviation of the response and the slope of the calibration curve (ICH ICoH, 2005; Ravisankar and Sankar, 2015). The formulas for calculating LOD and LOQ are:

$$LOD=3.3X\left(\frac{\sigma }{S}\right)\text{ and }LOQ=10X\left(\frac{\sigma }{S}\right)$$

where, σ = standard deviation of the response and S = slope of the calibration curve.

From the linearity test, we calculated σ = 96.07 and S = 971.04. Applying the respective formulas yielded an LOD of 0.33 ppm and an LOQ of 0.99 ppm, which closely aligns with our signal-to-noise ratio results (LOD = 0.31 ppm, LOQ = 1 ppm). These calculations comply with the guidelines established by the ICH and USP for analytical method validation.

## Conclusion

A reversed-phase HPLC method has been successfully developed and validated for the quantitative of Rivaroxaban in raw materials and pharmaceutical dosage forms. The method employs isocratic elution with an optimized mobile phase consisting of 25 mM potassium phosphate monobasic buffer (pH 2.9) and acetonitrile (70:30, v/v), delivered at a flow rate of 1.0 mL/min. The separation was performed using a Thermo Hypersil ODS C18 column (4.6 × 250 mm, 5 µm) on an Agilent Technologies 1,100 Series HPLC system, equipped with a multi-wavelength diode array detector (MWD/DAD). The injection volume was set to 15 μL, with detection carried out at 249 nm under ambient temperature conditions.

The results of the system suitability test (SST) confirmed that all chromatographic peaks achieved complete resolution, with a tailing factor close to 1, indicating excellent peak symmetry and optimal column efficiency. The theoretical plate count exceeded the recommended threshold, demonstrating high column performance and separation efficiency. The method exhibited high specificity, with no detectable interference from excipients, degradation products, or other impurities. Solution stability studies showed no evidence of peak loss, degradation, or additional peaks between the first and last injections, confirming the stability of Rivaroxaban in the prepared solutions over the analysis period. Furthermore, the peak purity factor exceeded the acceptance threshold, ensuring that the Rivaroxaban peak remained free from spectral overlap or co-eluting impurities.

The method demonstrated excellent linearity over the validated concentration range, with a correlation coefficient (R^2^) of 0.9990, indicating a strong linear relationship between concentration and peak response. The calculated limit of detection (LOD) was 0.3 ppm, supported by a signal-to-noise ratio of 4.0, which meets the regulatory acceptance criterion of ≥3. The LOQ was also 1 ppm, ensuring precise and reproducible quantification at lower analyte concentrations.

A comprehensive validation was performed in accordance with FDA, USP, and ICH guidelines (ICH Q2 (R1)), assessing key parameters such as system suitability, specificity, linearity, accuracy, precision, robustness, solution stability, LOD, and LOQ. The method demonstrated high accuracy, with recovery values within the acceptable range (98.0%–102.0%), and precision, with %RSD values consistently below 2.0%, confirming the reproducibility of the results. Robustness testing confirmed the method’s reliability under deliberate variations in chromatographic conditions, such as mobile phase composition, flow rate, column temperature, and detection wavelength. This validated, stability-indicating HPLC method is highly suitable for routine quality control, stability studies, and pharmaceutical analysis of Rivaroxaban in both raw materials and finished dosage forms. Its simplicity, sensitivity, high throughput, and robustness make it an optimal choice for regulatory-compliant analytical applications in pharmaceutical laboratories. Additionally, its ability to detect potential degradants and impurities further supports its applicability in stability testing and formulation development.

## Data Availability

The original contributions presented in the study are contained in the article and/or the Supplementary Material. Further inquiries can be directed to the corresponding author.

## Abbreviations

ACN, Acetonitrile; Avg, Average; C18, Column having octadecyl chain of Carbon atom; FDA, Food and Drug Administration; HCl, hydrochloric acid; HPLC, High Performance Liquid Chromatography; ICH, International Conference on Harmonization; pKa, Ionization constant; LC, Liquid Chromatography; LOD, Limit of Detection; LOQ, Limit of Quantitation; μL, Micro liter; μg/mL, Microgram per Milliliter; min, Minutes; N, Normality; Mg, Milligram; ppm, Parts per million; ODS, Octadecylsilane; pH, negative logarithm of H+ concentration; RP-HPLC, Reversed Phase High Performance Liquid Chromatography; RSD, Relative Standard Deviation; RT, Retention time; r^2^, Correlation of coefficient; STD, Standard Deviation; UV, Ultraviolet.
