# Supplementary material for: Comprehensive and robust stability-indicating reversed phase high performance liquid chromatography (RP-HPLC) method for Rivaroxaban: synergistic integration of infrared spectroscopy and clinical pharmacology insights
Source: Front Chem. 2025 Apr 25;13:1551189. doi: 10.3389/fchem.2025.1551189 (PMC12062752; doi:10.3389/fchem.2025.1551189)
Supplement: Supplementary file 1 [file DataSheet1.docx]

Supplementary Material

# Supplementary Procedures

**Solution Preparation Procedures**

**Preparation of Mobile Phase solutions**

**Mobile phase A (Buffer pH 2.9): to prepare one liter of 25 mM potassium phosphate monobasic solution at pH 2.90.**

Weigh 3.40 grams of Potassium Phosphate Monobasic and transfer it into a 1000 mL beaker. Add 1000 mL of DI water and stir until buffer salt is completely dissolved. Place a calibrated pH probe into the solution and adjust the pH by slowly adding phosphoric acid dropwise. Stop once the desired pH of 2.9 is reached. Filter the buffer by using (0.45µm membrane filter) and sonicate for 20 minutes to remove any air bubbles.

**Mobile phase A (Buffer pH 5.0): to prepare one liter of 25 mM sodium acetate solution at pH 5.0**

Weigh 2.05 g of Sodium Acetate and transfer it into a 1000 mL beaker. Add 1000 mL of DI water and stir until buffer salt is completely dissolved. Place a calibrated pH probe into the solution and adjust the pH by slowly adding glacial acetic acid dropwise. Stop once the desired pH of 5.0 is reached. Filter the buffer by using (0.45μm membrane filter) and sonicate for 20 minutes to remove any air bubbles.

**Mobile phase A (Buffer pH 7.0): to prepare one liter of 25 mM potassium phosphate dibasic solution at pH 7.0**

Weigh 4.36 g of Potassium Phosphate Dibasic and transfer it into a 1000 mL beaker. Add 1000 mL of DI water and stir until buffer salt is completely dissolved. Place a calibrated pH probe into the solution and adjust the pH by slowly adding phosphoric acid dropwise. Stop once the desired pH of 7.0 is reached. Filter the buffer by using (0.45μm membrane filter) and sonicate for 20 minutes to remove any air bubbles.

**Mobile phase B (100% Acetonitrile)**

Transfer 1000 mL of ACN into the mobile phase reservoir and sonicate for 20 minutes to remove air bubbles.

**Stock solution of Rivaroxaban (10,000 ppm)**

Weigh 500 mg of Rivaroxaban and transfer it into a 50 mL volumetric flask. Add 25 mL of ACN: DI water (70:30 v/v) and sonicate for 20 minutes or until Rivaroxaban is completely dissolved. Complete the volume to the mark with ACN: DI water (70:30 v/v) and shake it thoroughly.

**Stock solution of Rivaroxaban (1,000 ppm)**

Transfer 5.0 mL of stock solution Rivaroxaban (10,000ppm) into a 50 ml volumetric flask. Complete the volume to the mark with ACN: DI water (70:30 v/v) and shake it thoroughly.

**Preparation of Standard Solutions for Linearity Study**

**Stock solution of Rivaroxaban (5,000 ppm)**

Weigh 250 mg of Rivaroxaban and transfer it into a 50 mL volumetric flask. Add 25 mL of ACN: DI water (70:30 v/v) and sonicate for 20 minutes until Rivaroxaban is completely dissolved. Complete the volume to the mark with ACN: DI water (70:30 v/v) and shake it thoroughly.

**Stock solution of Rivaroxaban (850 ppm)**

Transfer 8.5 mL of stock solution Rivaroxaban (5,000ppm) into a 50 mL volumetric flask. Complete the volume to the mark with ACN: DI water (70:30 v/v) and shake it thoroughly.

**Stock solution of Rivaroxaban (800 ppm)**

Transfer 8.0 mL of stock solution Rivaroxaban (5,000ppm) into a 50 mL volumetric flask. Complete the volume to the mark with ACN: DI water (70:30 v/v) and shake it thoroughly.

**Stock solution of Rivaroxaban (750 ppm)**

Transfer 7.5 mL of stock solution Rivaroxaban (5,000ppm) into a 50 mL volumetric flask. Complete the volume to the mark with ACN: DI water (70:30 v/v) and shake it thoroughly.

**Stock solution of Rivaroxaban (700 ppm)**

Transfer 7.0 mL of stock solution Rivaroxaban (5,000ppm) into a 50 mL volumetric flask. Complete the volume to the mark with ACN: DI water (70:30 v/v) and shake it thoroughly.

**Stock solution of Rivaroxaban (650 ppm)**

Transfer 6.5 mL of stock solution Rivaroxaban (5,000ppm) into a 50 mL volumetric flask. Complete the volume to the mark with ACN: DI water (70:30 v/v) and shake it thoroughly.

**Stock solution of Rivaroxaban (600 ppm)**

Transfer 6.0 mL of stock solution Rivaroxaban (5,000ppm) into a 50 mL volumetric flask. Complete the volume to the mark with ACN: DI water (70:30 v/v) and shake it thoroughly.

**Stock solution of Rivaroxaban (550 ppm)**

Transfer 5.5 mL of stock solution Rivaroxaban (5,000ppm) into a 50 mL volumetric flask. Complete the volume to the mark with ACN: DI water (70:30 v/v) and shake it thoroughly.

**Stock solution of Rivaroxaban (500 ppm)**

Transfer 5.0 mL of stock solution Rivaroxaban (5,000ppm) into a 50 mL volumetric flask. Complete the volume to the mark with ACN: DI water (70:30 v/v) and shake it thoroughly.

**Solutions Preparation for Accuracy Study**

**Stock solution of Rivaroxaban (5,000 ppm):**

Weigh 250 mg of Rivaroxaban and transfer it into a 50 mL volumetric flask. Add 25 mL of ACN: DI water (70:30 v/v) and sonicate for 20 minutes or until Rivaroxaban is completely dissolved. Complete the volume to the mark with ACN: DI water (70:30 v/v) and shake it thoroughly.

**Stock solution of Rivaroxaban (850 ppm)**

Transfer 8.5 mL of stock solution Rivaroxaban (5000ppm) into a 50 mL volumetric flask. Complete the volume to the mark with ACN: DI water (70:30 v/v) and shake it thoroughly.

**Stock solution of Rivaroxaban (700 ppm)**

Transfer 7.0 mL of stock solution Rivaroxaban (5000ppm) into a 50 mL volumetric flask. Complete the volume to the mark with ACN: DI water (70:30 v/v) and shake it thoroughly.

**Stock solution of Rivaroxaban (500 ppm)**

Transfer 5.0 mL of stock solution Rivaroxaban (5000ppm) into a 50 mL volumetric flask. Complete the volume to the mark with ACN: DI water (70:30 v/v) and shake it thoroughly

**Solutions Preparation for Limit of Detection (LOD) Study**

**Stock solution of Rivaroxaban (5000 ppm)**

Weigh 250 mg of Rivaroxaban and transfer it into a 50 mL volumetric flask. Add 25 mL of ACN: DI water (70:30 v/v) and sonicate for 20 minutes or until Rivaroxaban is completely dissolved. Complete the volume to the mark with ACN: DI water (70:30 v/v) and shake it thoroughly.

**Stock solution of Rivaroxaban (10 ppm)**

Transfer 0.1 mL of stock solution Rivaroxaban (5000ppm) into a 50 mL volumetric flask. Complete the volume to the mark with ACN: DI water (70:30 v/v) and shake it thoroughly.

**Stock solution of Rivaroxaban (5.0 ppm)**

Transfer 12.5 mL of stock solution Rivaroxaban (10ppm) into a 25 mL volumetric flask. Complete the volume to the mark with ACN: DI water (70:30 v/v) and shake it thoroughly.

**Stock solution of Rivaroxaban (2.0 ppm)**

Transfer 5 mL of stock solution Rivaroxaban (10ppm) into a 25 mL volumetric flask. Complete the volume to the mark with ACN: DI water (70:30 v/v) and shake it thoroughly.

**Stock solution of Rivaroxaban (1.5 ppm)**

Transfer 3.75 mL of stock solution Rivaroxaban (10ppm) into a 25 mL volumetric flask. Complete the volume to the mark with ACN: DI water (70:30 v/v) and shake it thoroughly.

**Stock solution of Rivaroxaban (1.0 ppm)**

Transfer 2.5 mL of stock solution Rivaroxaban (10ppm) into a 25 mL volumetric flask. Complete the volume to the mark with ACN: DI water (70:30 v/v) and shake it thoroughly.

**Stock solution of Rivaroxaban (0.9 ppm)**

Transfer 2.25 mL of stock solution Rivaroxaban (10ppm) into a 25 mL volumetric flask. Complete the volume to the mark with ACN: DI water (70:30 v/v) and shake it thoroughly.

**Stock solution of Rivaroxaban (0.5 ppm)**

Transfer 1.25 mL of stock solution Rivaroxaban (10ppm) into a 25 mL volumetric flask. Complete the volume to the mark with ACN: DI water (70:30 v/v) and shake it thoroughly.

**Stock solution of Rivaroxaban (0.4 ppm):**

Transfer 1 mL of stock solution Rivaroxaban (10ppm) into a 25 mL volumetric flask. Complete the volume to the mark with ACN: DI water (70:30 v/v) and shake it thoroughly.

**Stock solution of Rivaroxaban (0.3 ppm)**

Transfer 0.75 mL of stock solution Rivaroxaban (10ppm) into a 25 mL volumetric flask. Complete the volume to the mark with ACN: DI water (70:30 v/v) and shake it thoroughly.

**Stock solution of Rivaroxaban (0.2 ppm)**

Transfer 0.5 mL of stock solution Rivaroxaban (10ppm) into a 25 mL volumetric flask. Complete the volume to the mark with ACN: DI water (70:30 v/v) and shake it thoroughly.

**Solution Preparation for Mix Forced Degradation Study**

**Stock solution of 0.01N HCl**: Transfer 5 mL of 0.1N HCl solution into a 50 mL volumetric flask. Complete the volume to the mark with DI water and shake it thoroughly.

**Stock solution of 0.01N NaOH:** Transfer 10 ml of 1N NaOH solution into a 100 ml volumetric flask. Complete the volume to the mark with DI water and shake it thoroughly.

**Stock solution of 0.05% H2O2:** Transfer 10 ml of 0.5% H2O2 solution into a 100 ml volumetric flask. Complete the volume to the mark with DI water and shake it thoroughly.

**Acid stress sample preparation degraded with (0.01 N HCl)**

Transfer 2 mL of Rivaroxaban stock solution (3500 ppm) into a screw-cap test tube, add 2 mL of 0.01 N HCl into it, heat it on a heating block at 75 ºC for 24 hours. The solution was cooled at room temperature, add 2 mL of 0.01 N NaOH solution to neutralize the acid. The neutralized solution was accurately transferred into a 10 mL volumetric flask, complete the volume to the mark with (30:70 v/v) DI water: ACN, and shake it thoroughly to produce 700 ppm final concentration. It is very important to check the pH of the solution before injecting into the HPLC system using pH strips to make sure the solution is neutral (pH 7). The solution was filtered with 0.45 µm membrane filter before it was injected into the HPLC system.

**Base stress sample preparation degraded with (0.01N NaOH)**

Transfer 2 mL of Rivaroxaban stock solution (3500 ppm) into a screw-cap test tube, add 2 mL of 0.01N NaOH into it, heat it on a heating block at 75 ºC for 24 hours. The solution was cooled at room temperature, add 2 mL of 0.01N HCl solution to neutralize the basic solution. The neutralized solution was accurately transferred into a 10 mL volumetric flask, complete the volume to the mark with (30:70 v/v) DI water: ACN, and shake it thoroughly to produce 700 ppm final concentration. It is very important to check the pH of the solution before injected into the HPLC system using pH strips in order make sure the solution is neutral (pH 7). The solution was filtered with 0.45 µm membrane filter before it was injected into the HPLC system.

**Oxidation stress sample preparation degraded with (0.05% H_2_O_2_):**

Transfer 2 mL of Rivaroxaban stock solution (3500 ppm) into a screw-cap test tube, add 2 mL of 0.05% H_2_O_2_ into it, heat it on a heating block at 75 ºC for 24 hours. The solution was cooled at room temperature, the solution was accurately transferred into a 10 mL volumetric flask, complete the volume to the mark with (30:70 v/v DI water: ACN, and shake it thoroughly to produce 700 ppm final concentration. The solution was filtered with 0.45 µm membrane filter before it was injected into the HPLC system.

**Standard solutions preparation for solution stability**

***Stock standard solution of Rivaroxaban (10,000 ppm)***

Weigh 500 mg of Rivaroxaban and transfer it into a 50 mL volumetric flask. Add 25 mL of ACN: DI water (70:30 v/v) and sonicate for 20 minutes or until Rivaroxaban is completely dissolved. Complete the volume to the mark with ACN: DI water (70:30 v/v) and shake it thoroughly.

***Working standard solution of Rivaroxaban (700 ppm)***

Transfer 3.5 mL of stock solution Rivaroxaban (10,000ppm) into a 50 ml volumetric flask. Complete the volume to the mark with ACN: DI water (70:30 v/v) and shake it thoroughly.

# Supplementary Figures and Tables

## Supplementary Figures


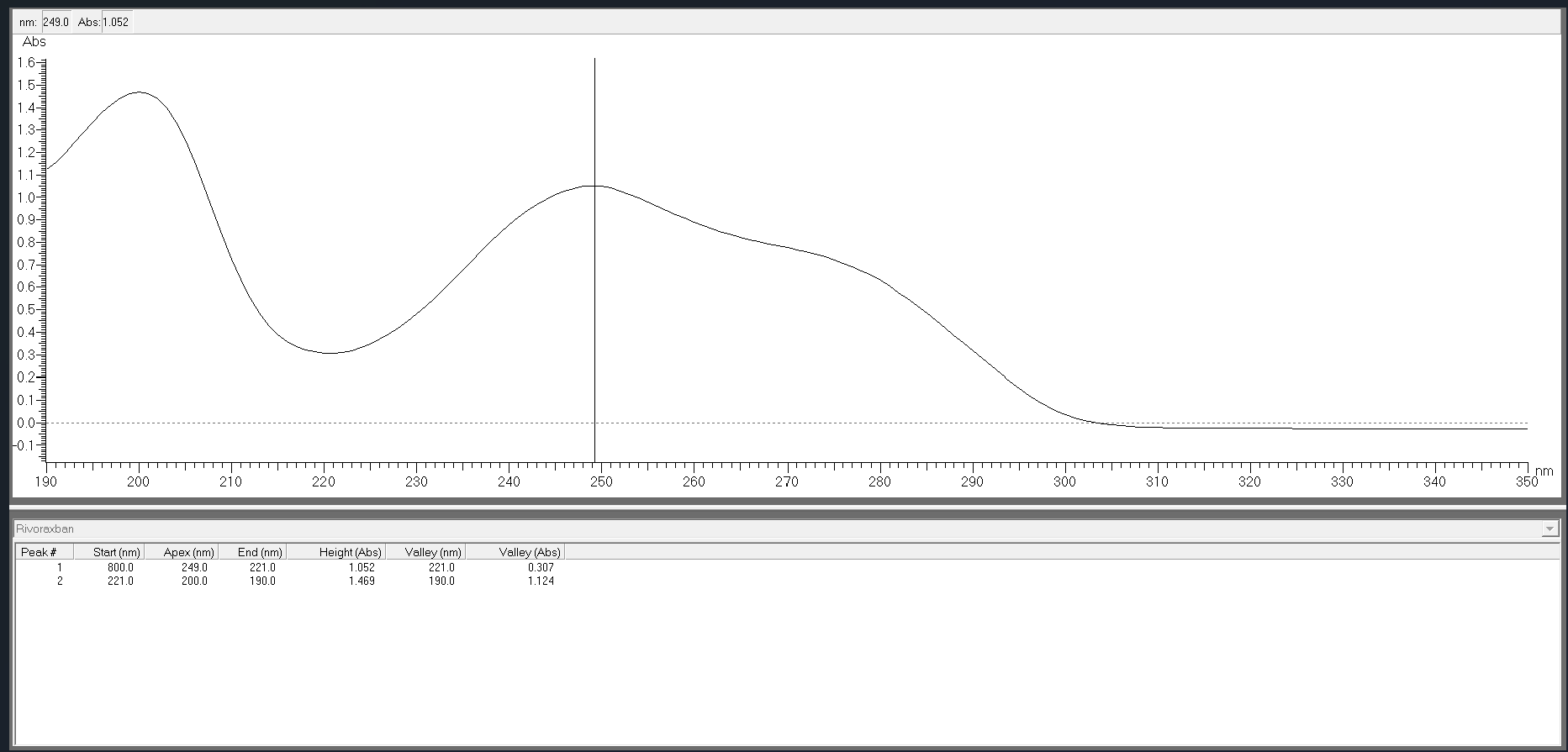


**Figure S1.** UV spectrum of Rivaroxaban using a Hitachi UV/VIS Double Beam Spectrophotometer, Model U-2900


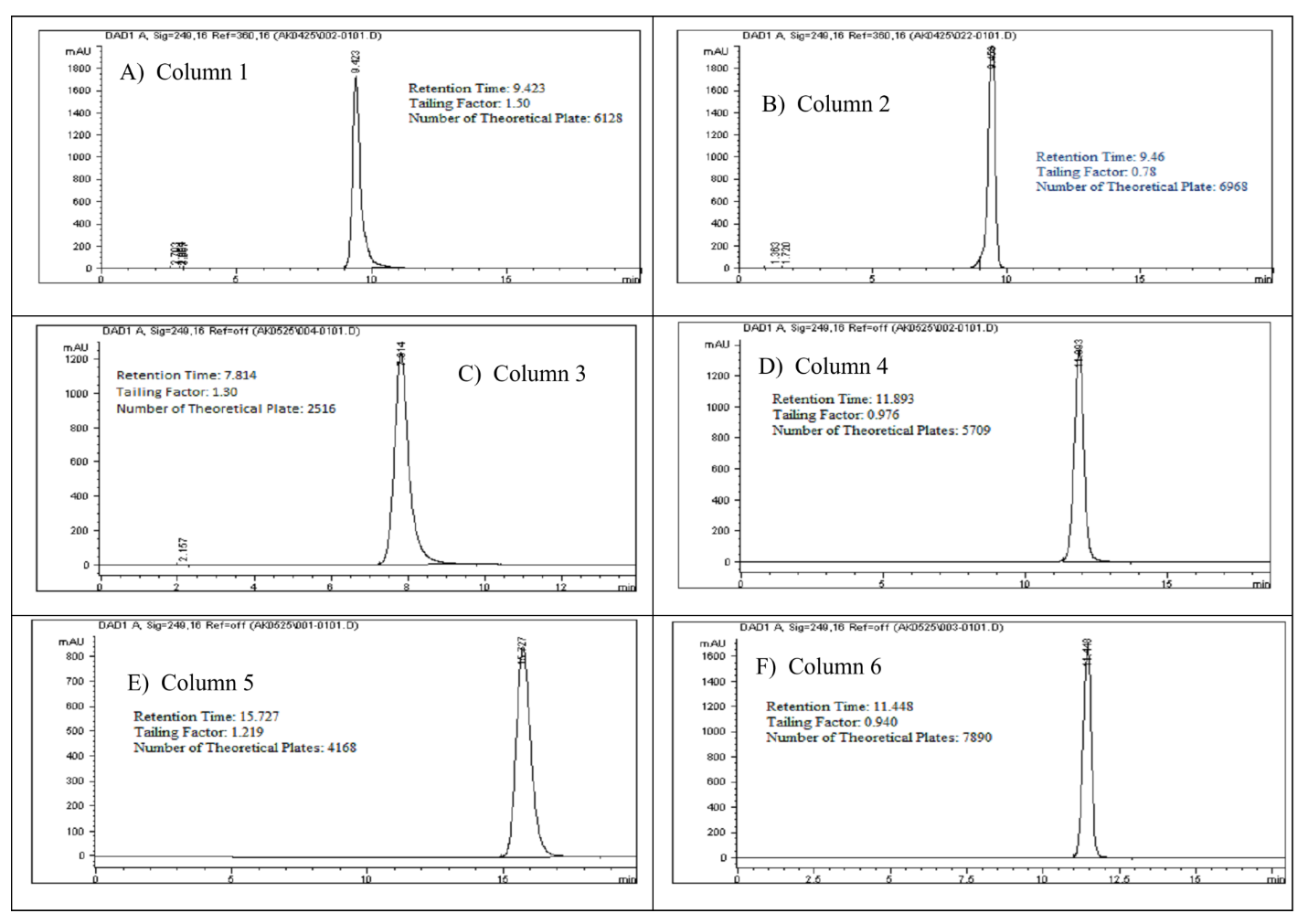


**Figure S2.** Chromatograms of columns 1 through 6 of 1000 ppm injection of Rivaroxaban

1. Column 1: Phenomenex C18 (4.6x150mm, 5µm)
2. Column 2: Water C_18_ (4.6x150 mm, 5µm)
3. Column 3: Agilent Zorbax Rx-C18 (4.6x250 mm, 5µm)
4. Column 4: Water XTERRA RP-18 (4.6x250 mm, 5µm)
5. Column 5: Phenomenex C18 (4.6x250 mm, 5µm)
6. Column 6: Thermo hypersil ODS C18 (4.6x250 mm, 5µm)

**Chromatographic conditions:** Isocratic elution, mobile phase 30:70 ACN/25 mM potassium phosphate buffer monobasic pH 2.9, flow rate 1.0 mL/min, detection wavelength at 249 nm, ambient temperature, 15 µL injection volume.

| 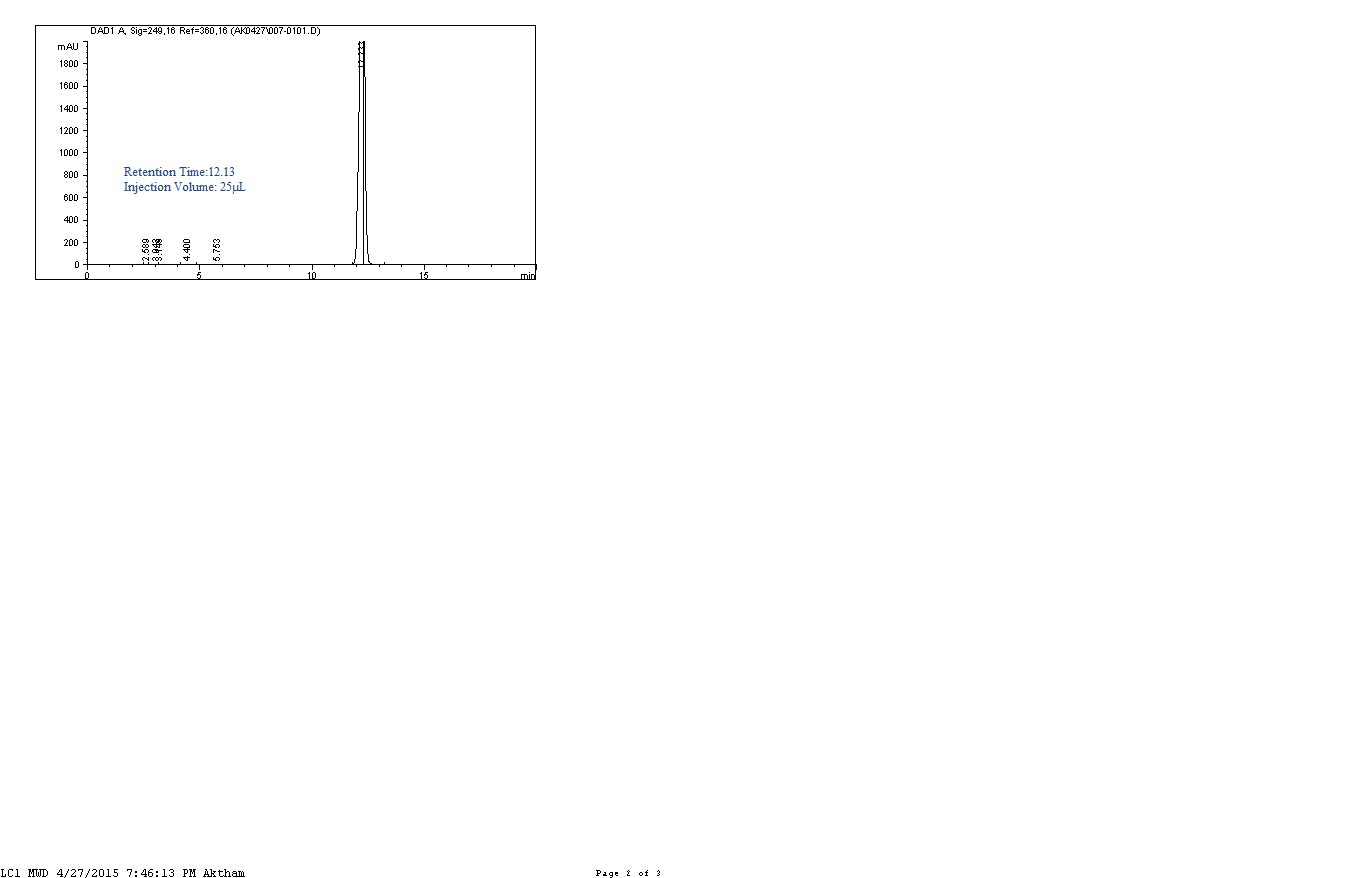  A) | 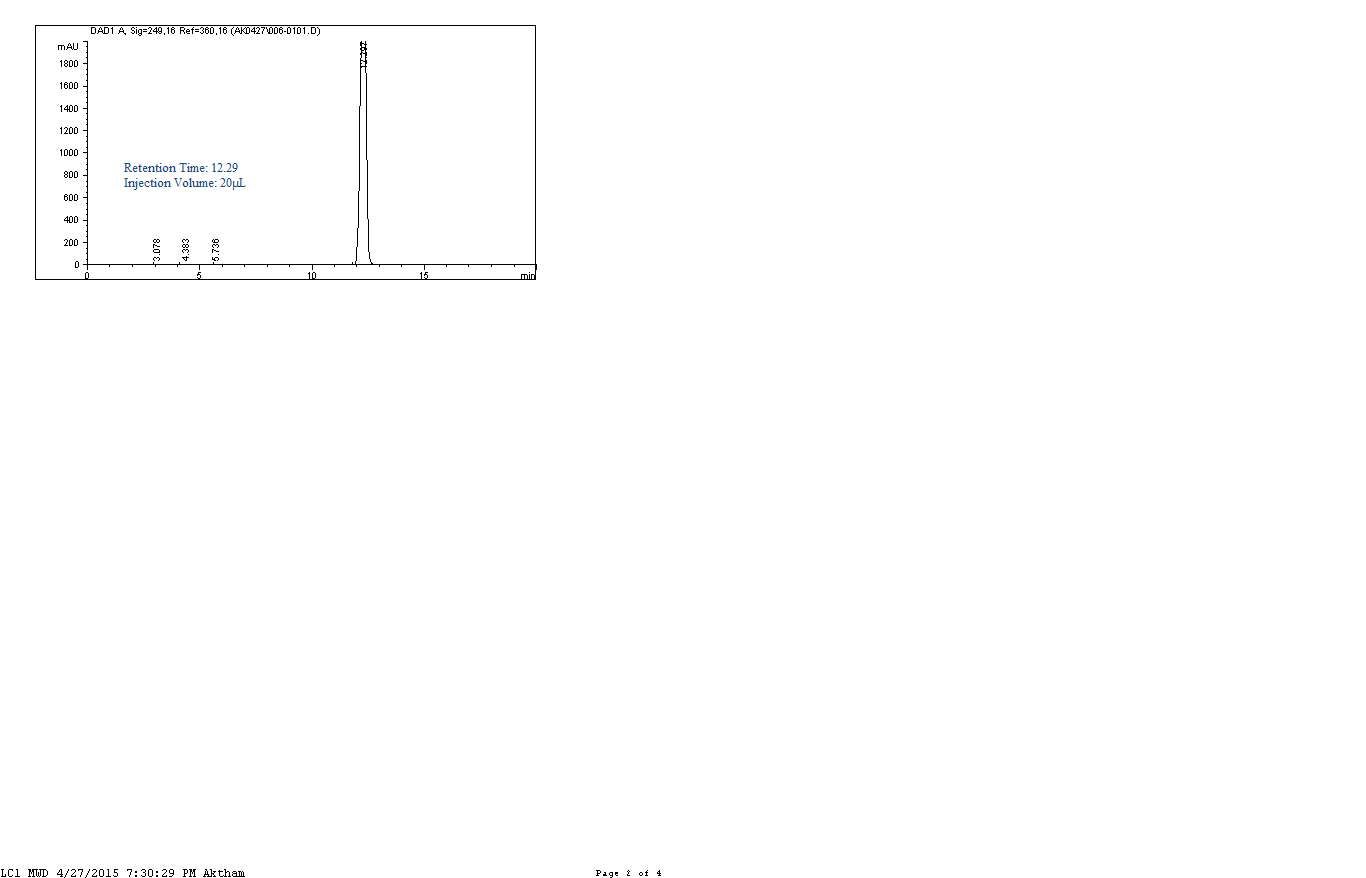  B) |
| --- | --- |
| 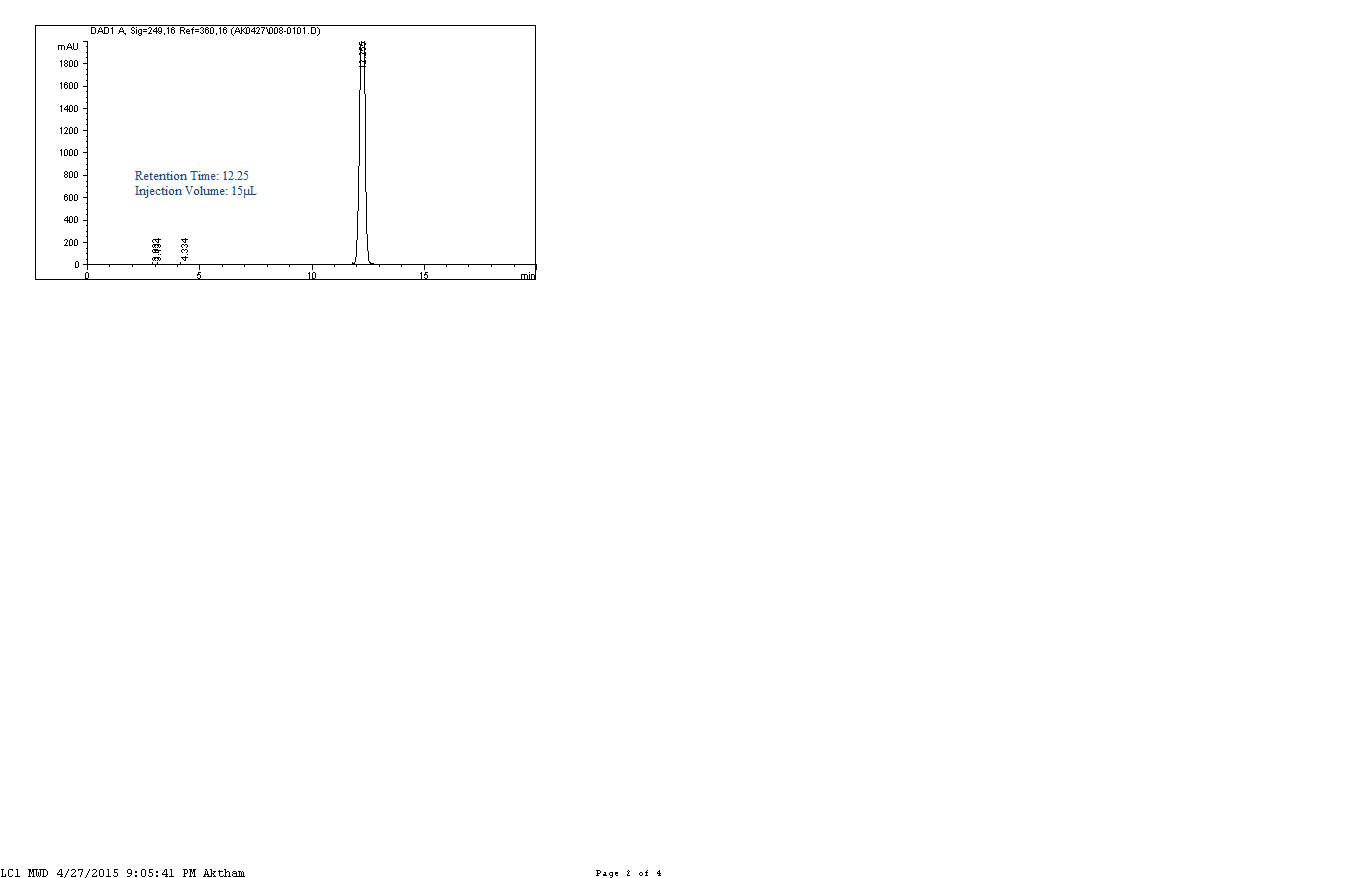  C) | 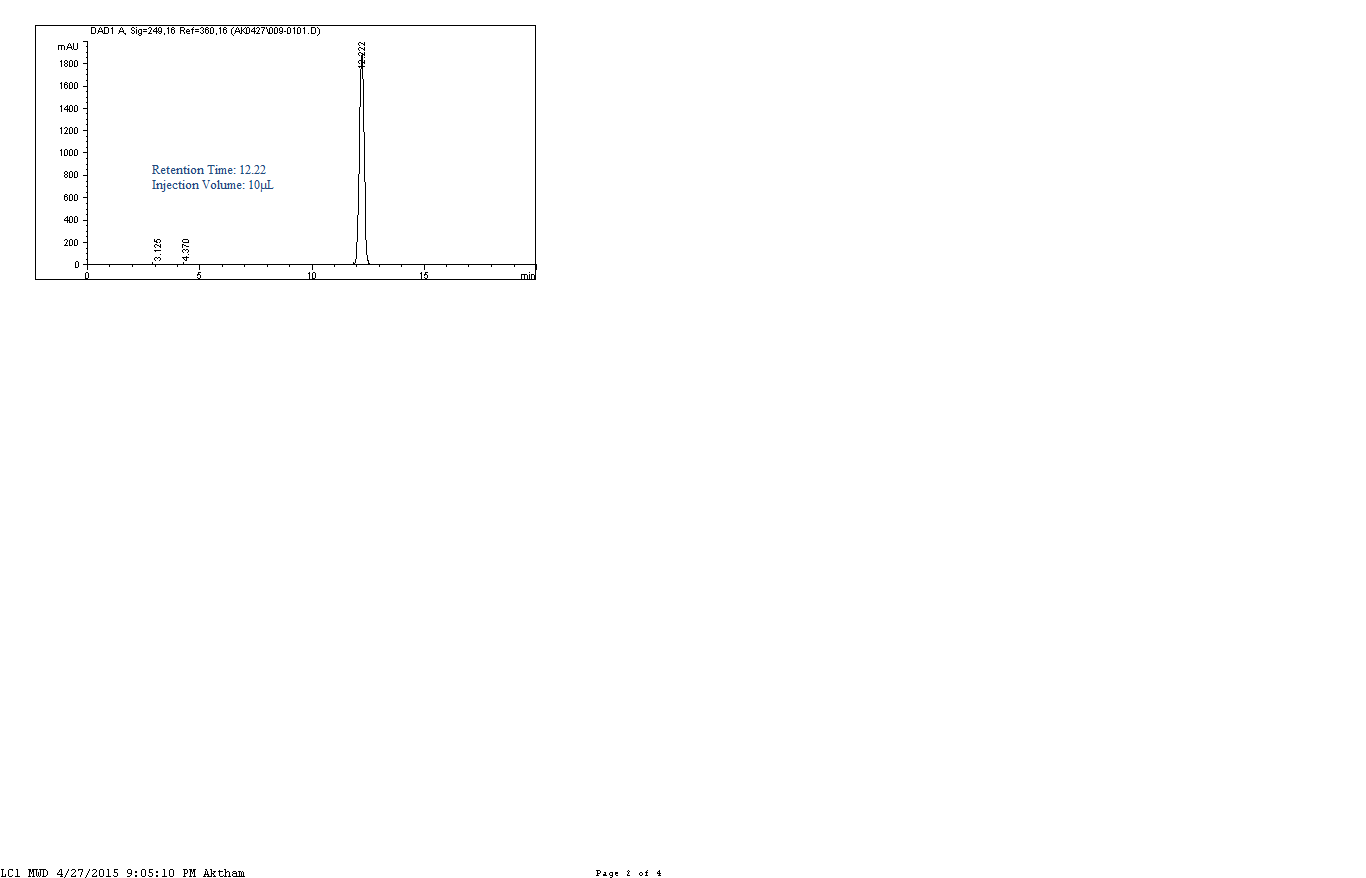  D) |

**Figure S3.** Chromatograms of Rivaroxaban with a different injection volume of Rivaroxaban

1. Injection volume: 25µL C) Injection volume: 15µL
2. Injection volume: 20µL D) Injection volume: 10µL

**Chromatographic conditions:** Isocratic elution, mobile phase 30:70 ACN/25 mM potassium phosphate buffer monobasic pH 2.9, flow rate 1.0 mL/min, detection wavelength at 249 nm, ambient temperature, thermo hypersil ODS C_18_ (4.6x250 mm, 5µm) column.

**
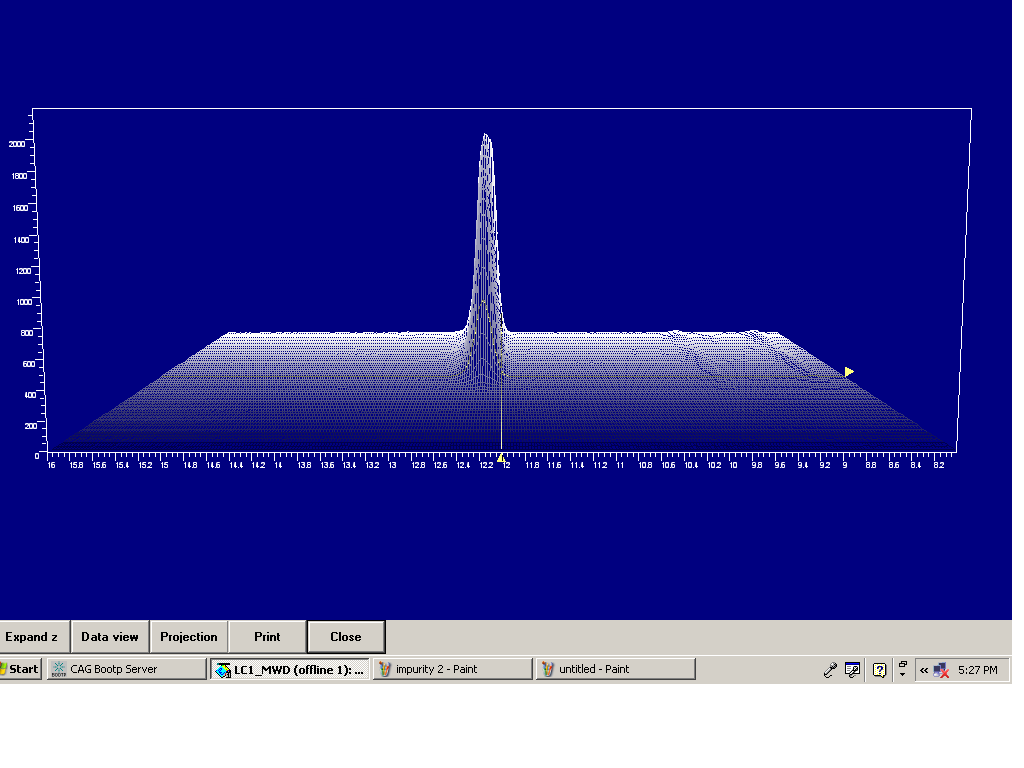
**

**Figure S4.** Three-dimension images of Rivaroxaban peak purity.

**Chromatographic conditions:** Isocratic elution, mobile phase 30:70 ACN/25 mM potassium phosphate buffer monobasic pH 2.9, flow rate 1.0 mL/min, detection wavelength at 249 nm, ambient temperature, 15 µL injection volume, thermo hypersil ODS C_18_ (4.6x250 mm, 5µm) column

- **The variations made to the buffer pH of mobile phase were (2.9** $\boldsymbol{\pm}$ **0.2).**

| **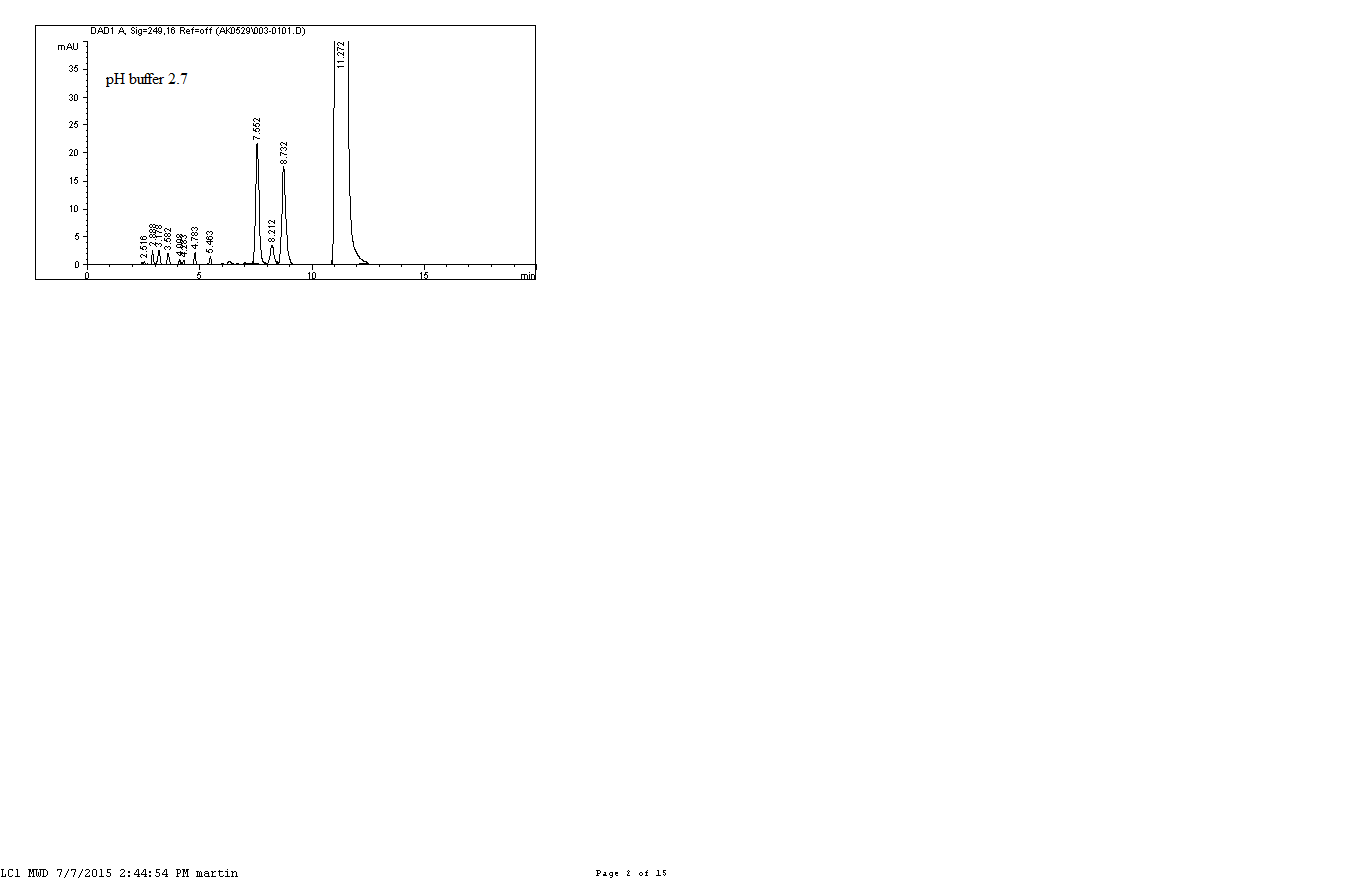** | **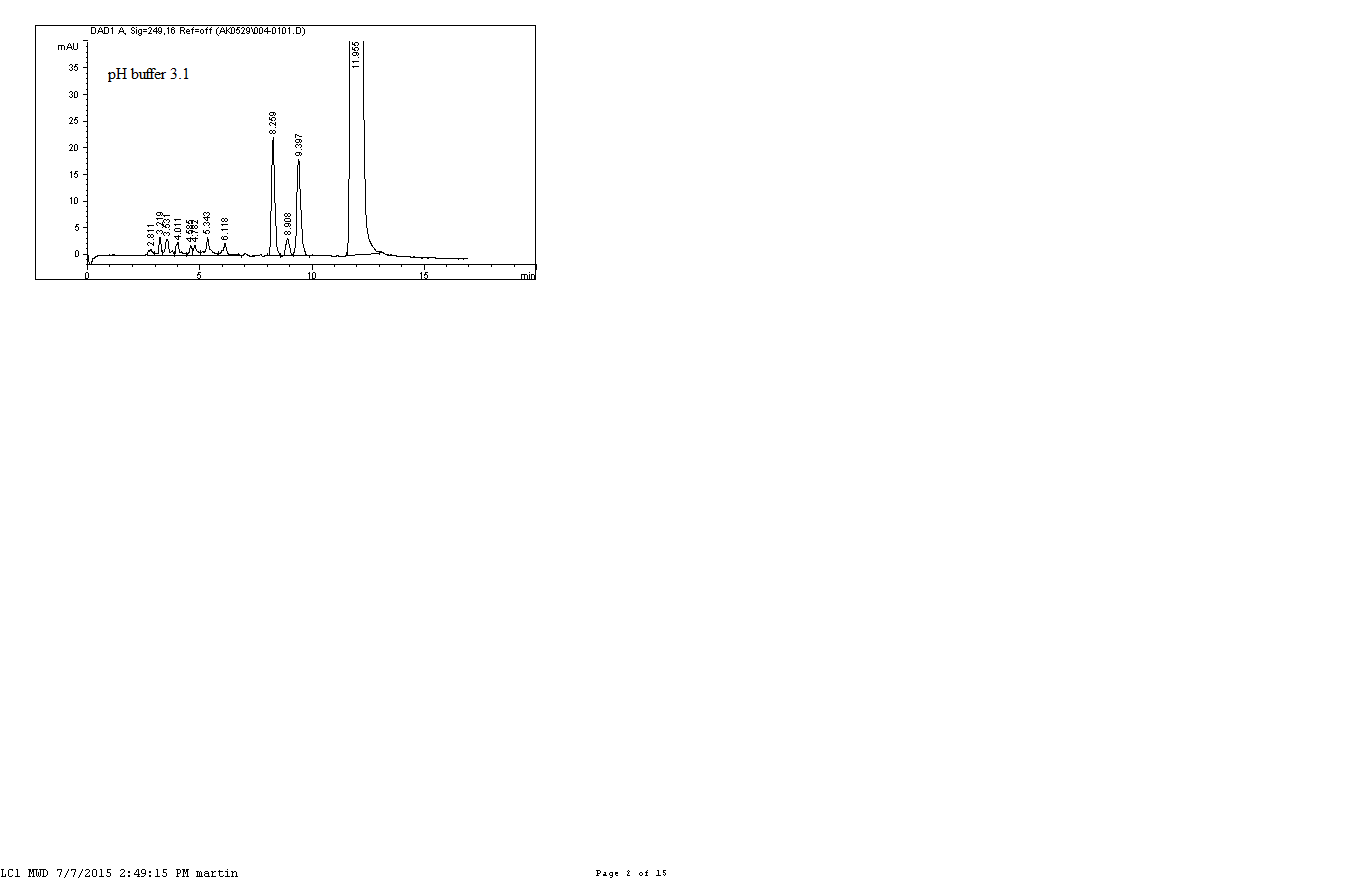** |
| --- | --- |
| **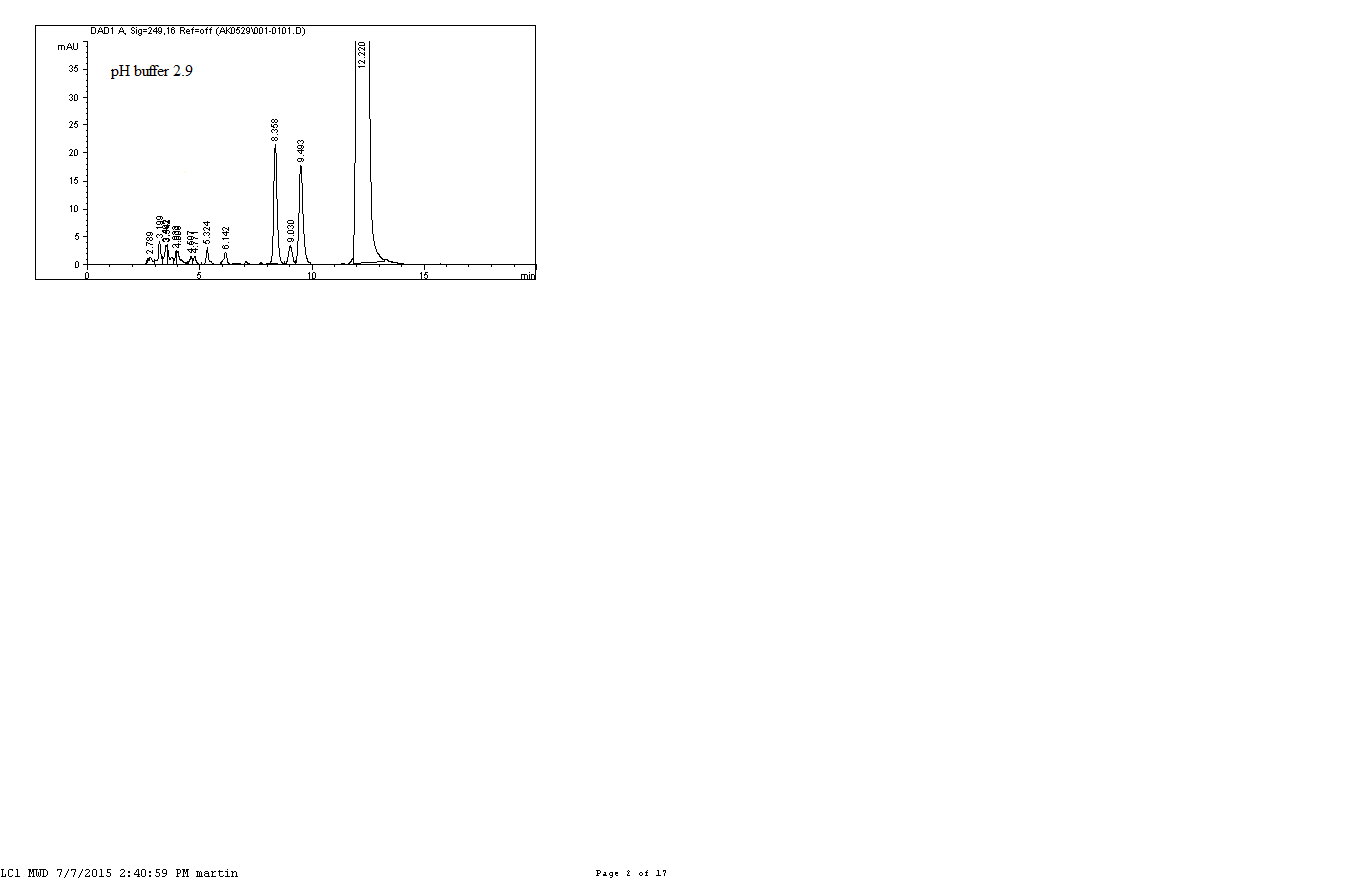** | |

**Figure S5:** Chromatograms of mixed degradation sample for buffer pH robustness study.

A) Buffer pH: 2.7 B) Buffer pH: 3.1

C) Buffer pH: 2.9 (Developed Method)

**Chromatographic conditions:** Isocratic elution, mobile phase 30:70 ACN/25 mM potassium phosphate buffer monobasic, flow rate 1.0 mL/min, detection wavelength at 249 nm, ambient temperature, 15 µL injection volume, thermo hypersil ODS C_18_ (4.6x250 mm, 5µm) column.

- **The variations made to the Flow Rate of mobile phase were (1.0** $\boldsymbol{\pm}$ **0.2 mL/min).**

| 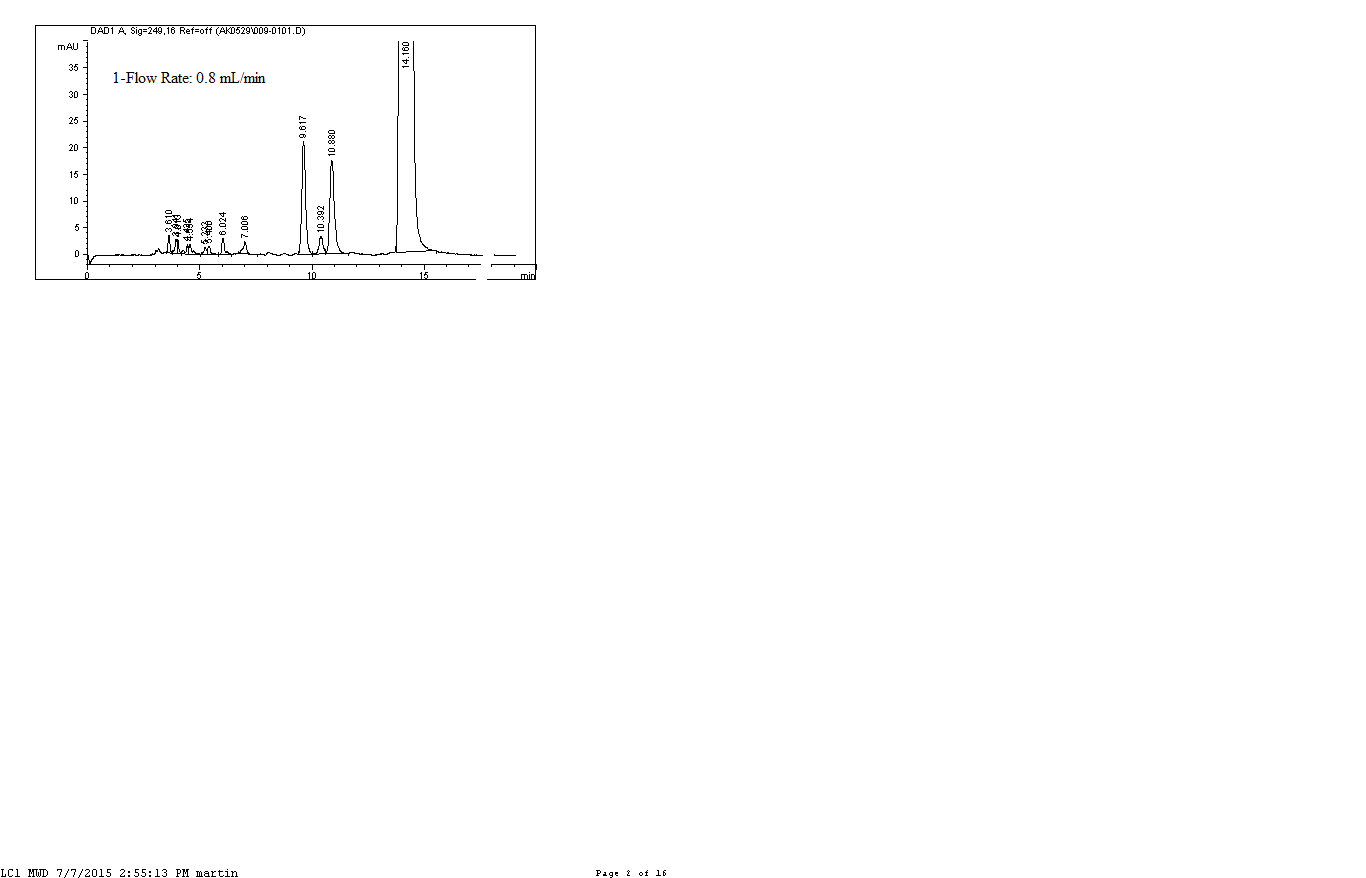 | 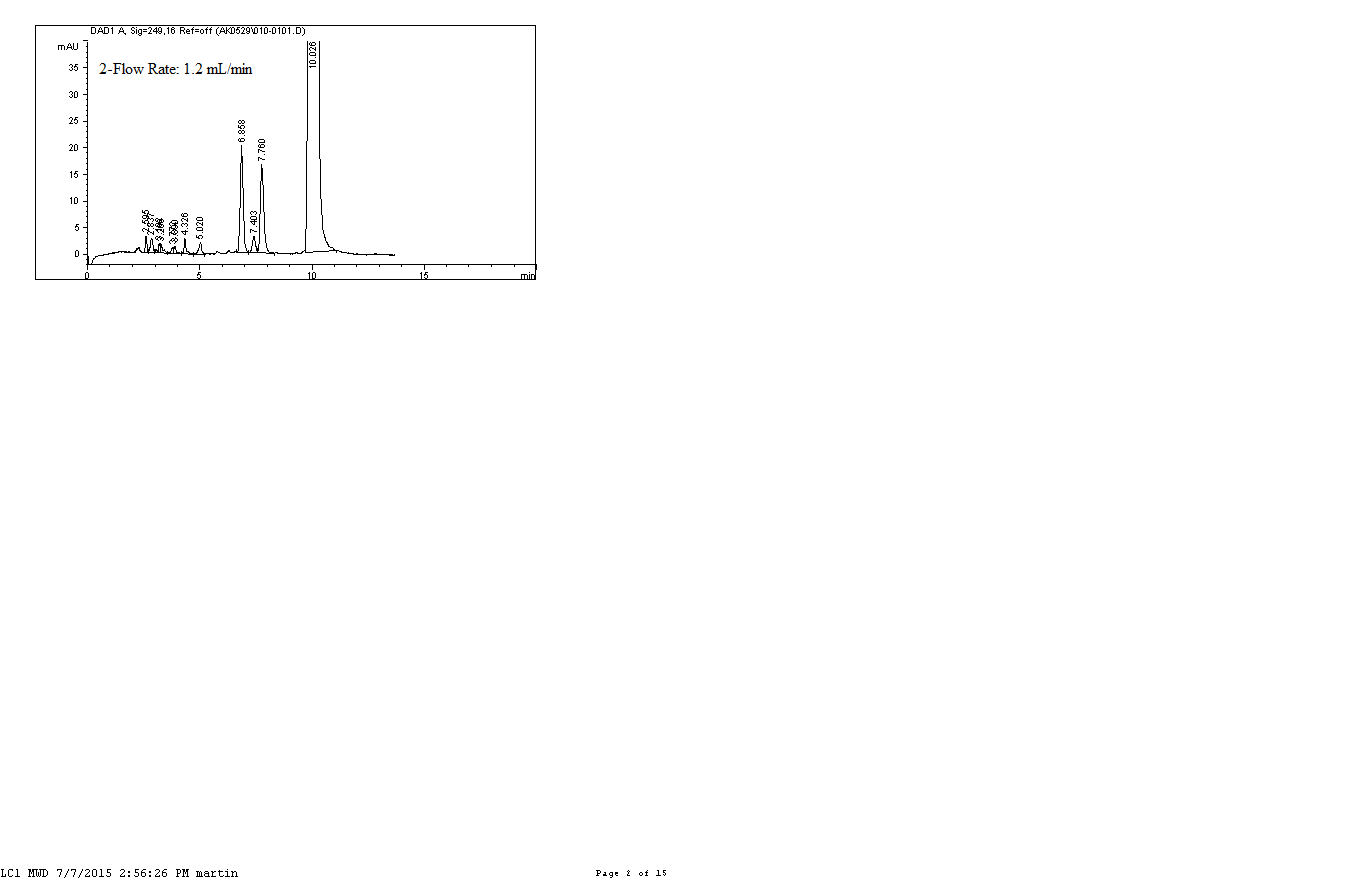 |
| --- | --- |
| 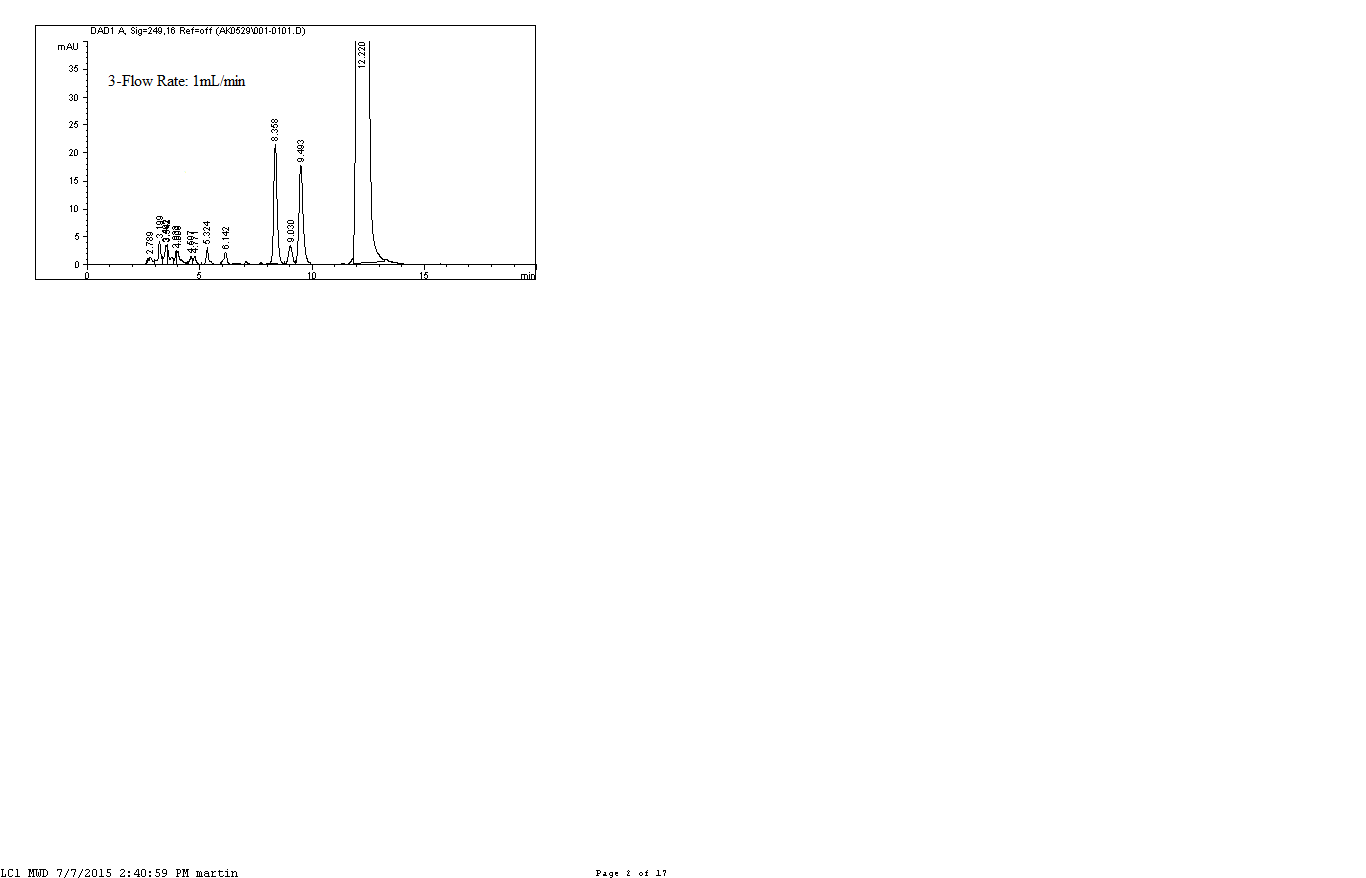 | |

**Figure S6.** Chromatograms of Method Robustness for different flow rate of mobile phase.

1. Flow rate: 0.80 mL/min 3) Flow rate: 1.0 mL/min (Developed Method)
2. Flow rate: 1.2 mL/min

**Chromatographic conditions:** Isocratic elution, mobile phase 30:70 ACN/25mM potassium phosphate buffer monobasic pH 2.9, detection wavelength at 249 nm, ambient temperature, 15 µL injection volume, thermo hypersil ODS C_18_ (4.6x250 mm, 5µm) column.

- **The variations made to the UV detection wavelength were (249** $\boldsymbol{\pm}$ **2 nm).**

| 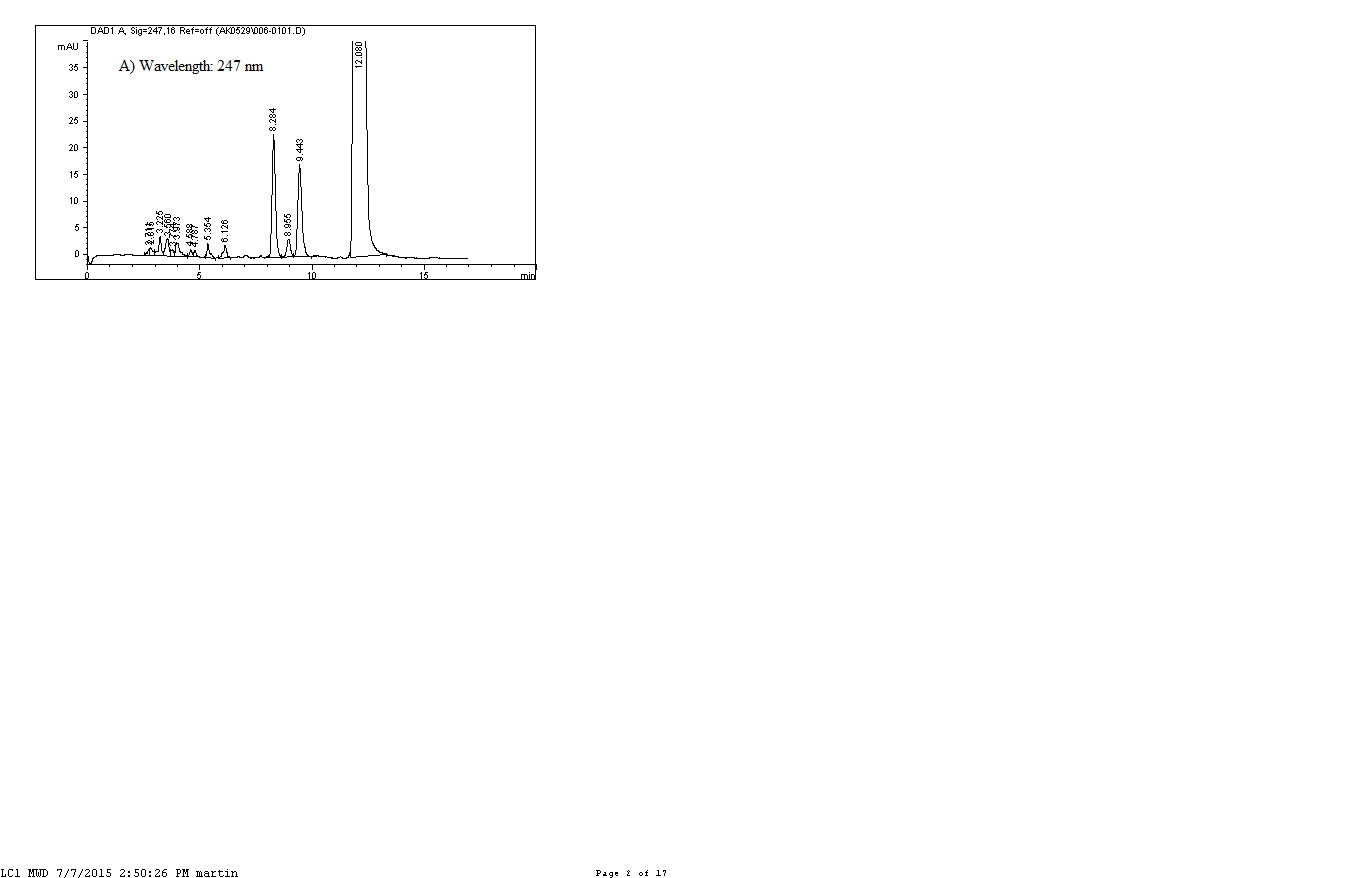 | 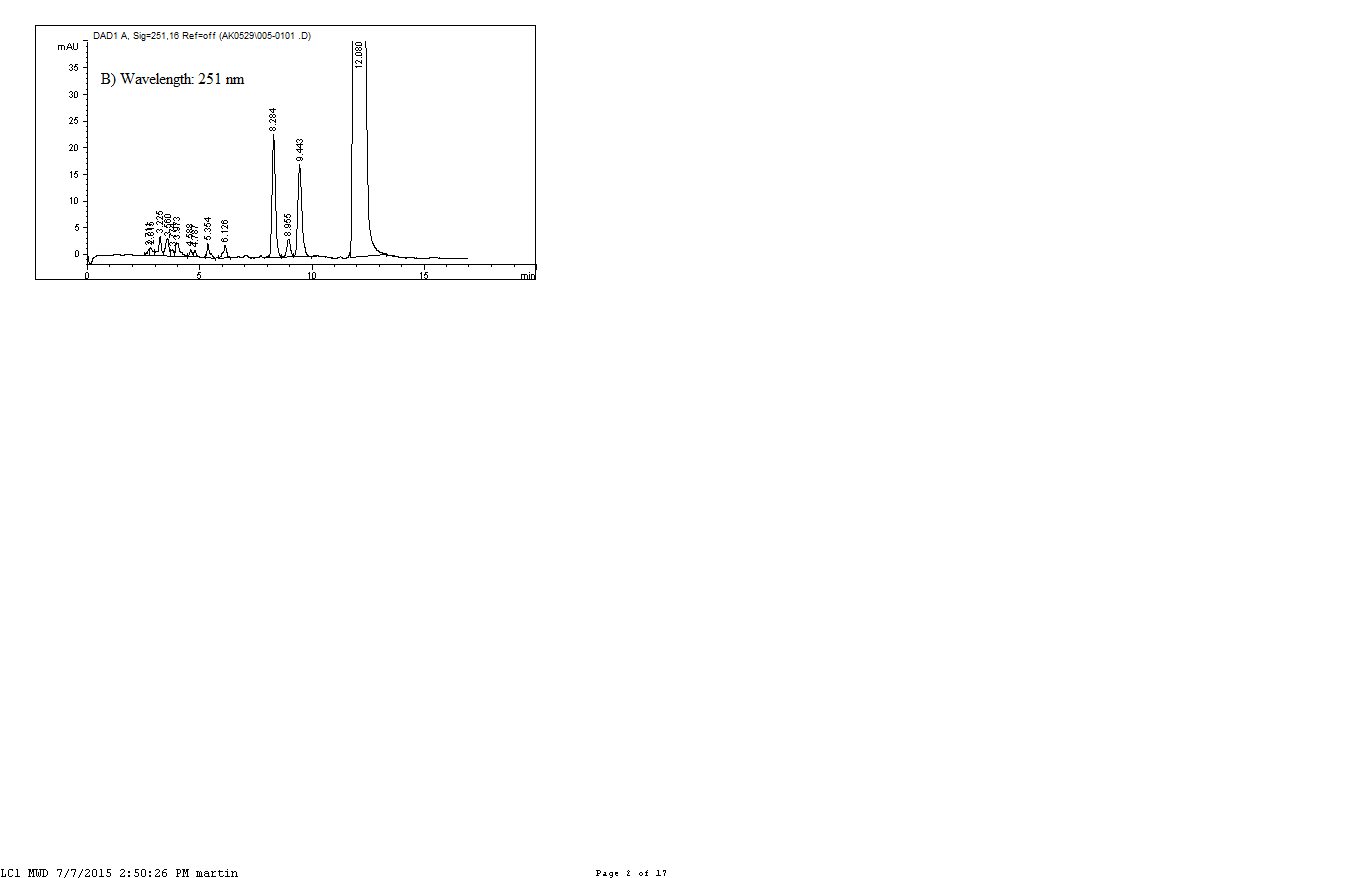 |
| --- | --- |
| 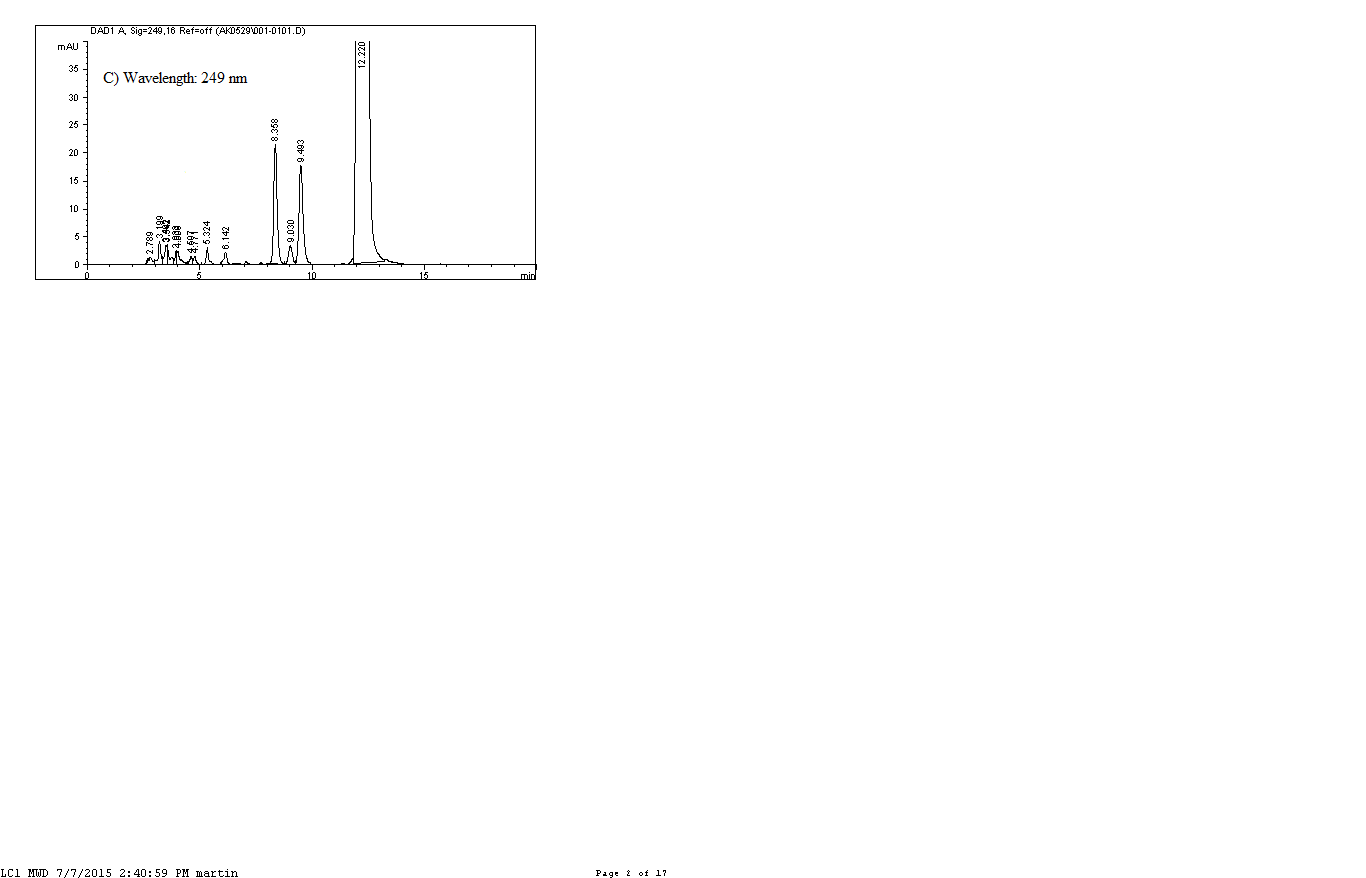 | |

**Figure S7:** Chromatograms of mixed degradation sample for UV wavelength robustness study

A) Wavelength: 247 nm C) Wavelength: 249 nm (Developed Method)

B) Wavelength: 251 nm

**Chromatographic conditions:** Isocratic elution, mobile phase 30:70 ACN/25 mM potassium phosphate buffer monobasic pH 2.9, flow rate 1.0 mL/min, ambient temperature, 15 µL injection volume, thermo hypersil ODS C_18_ (4.6x250 mm, 5µm) column.

- **The variations made to the Isocratic elution, ACN: Buffer were (30** $\boldsymbol{\pm}$ **5 B)**.

| 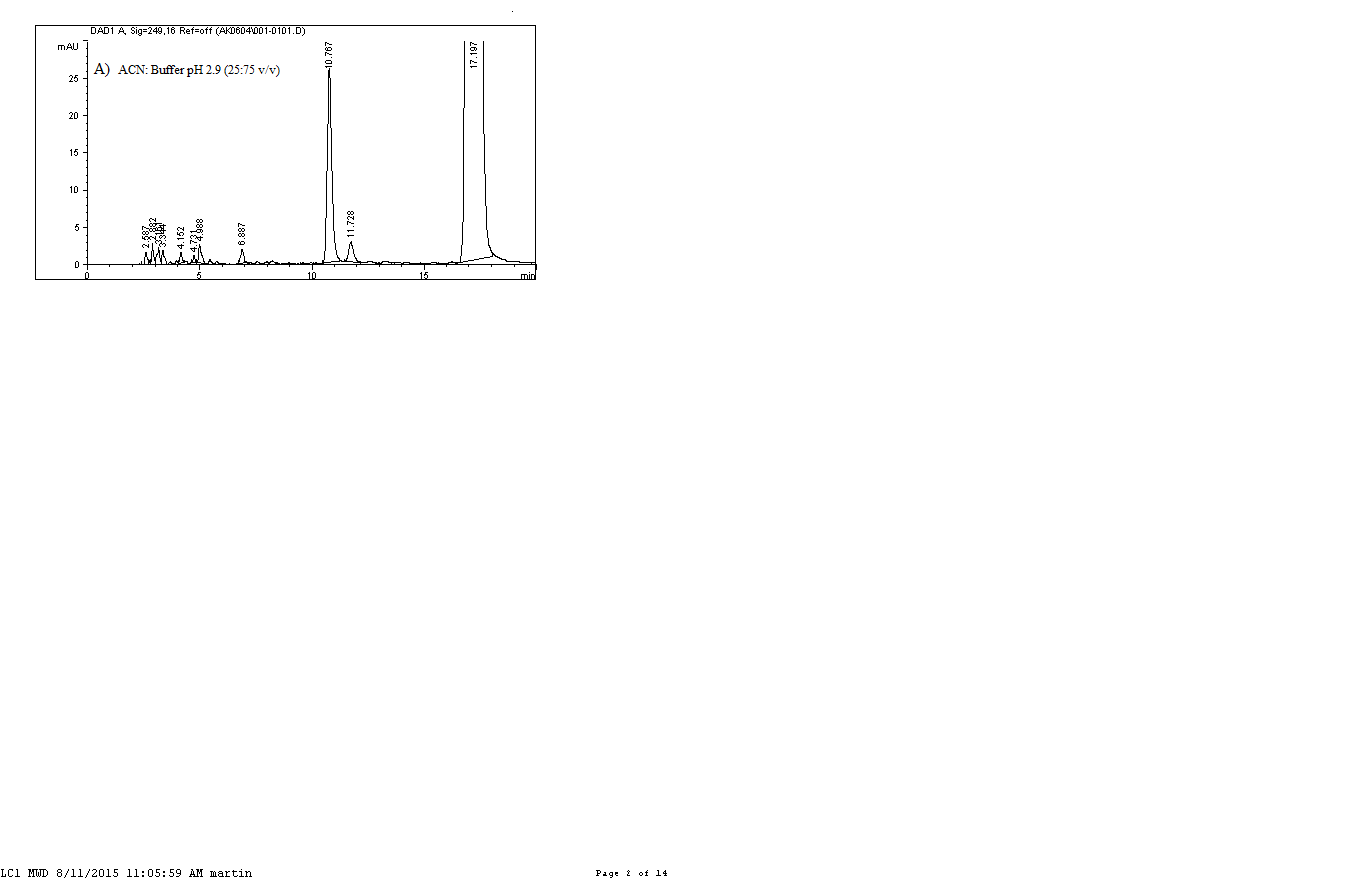 | 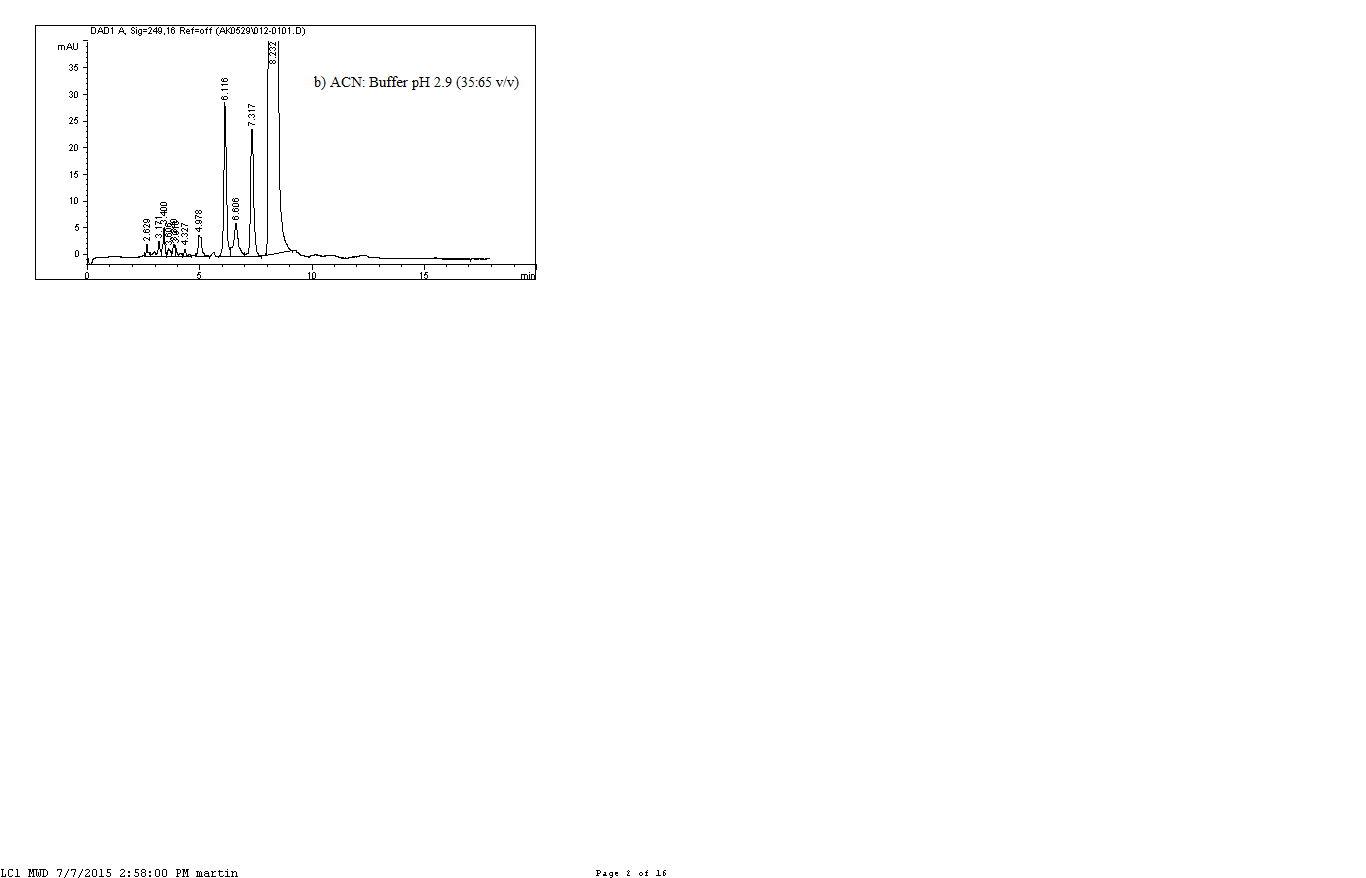 |
| --- | --- |
| 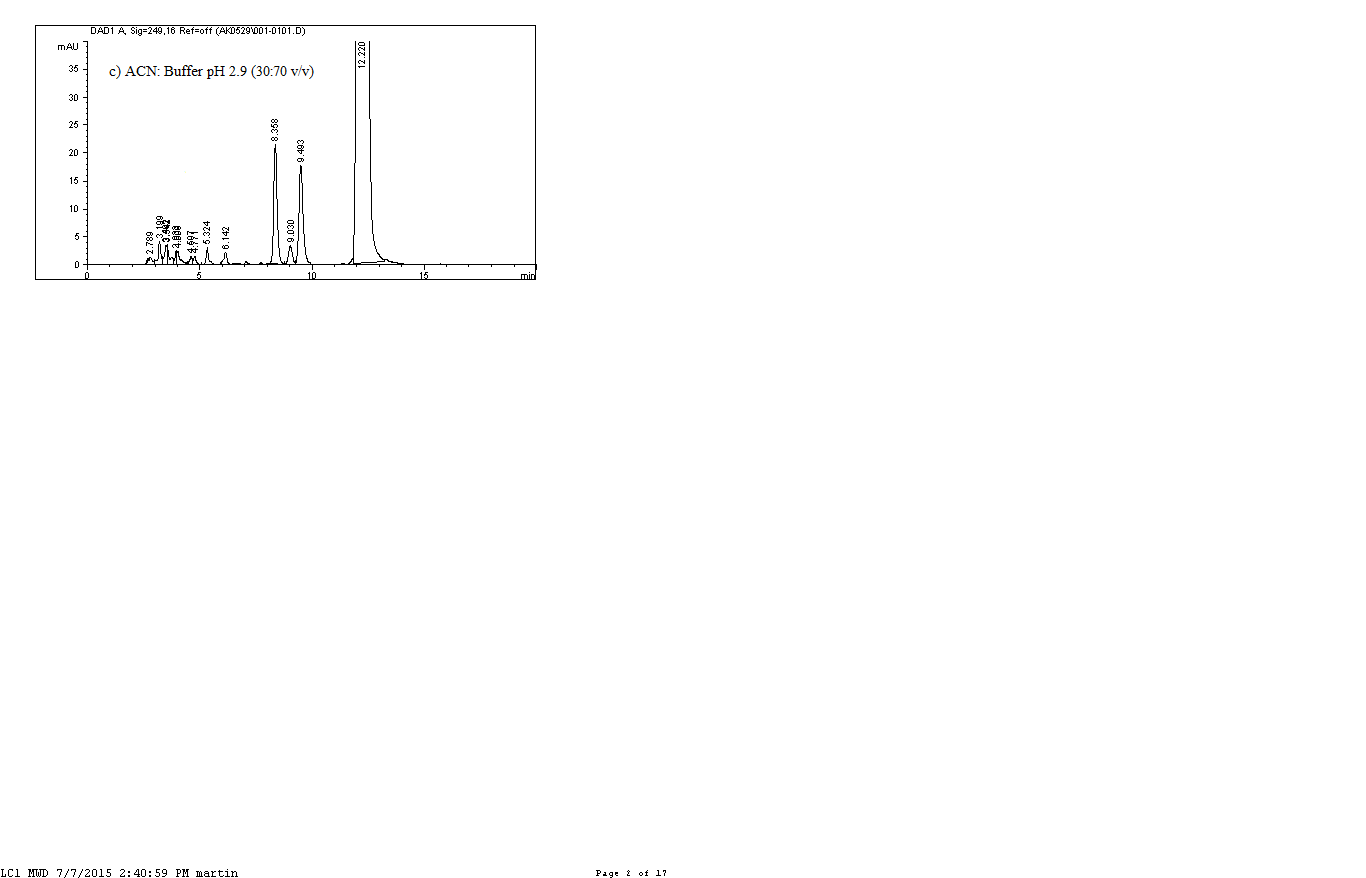 | |

**Figure S8:** Chromatograms of mixed degradation sample for solvent strength robustness study.

A) Solvent Strength: 25% ACN C) Solvent Strength: 30% ACN (Developed Method)

B) Solvent Strength: 35% ACN

**Chromatographic conditions:** Isocratic elution, mobile phase ACN/25 mM potassium phosphate buffer monobasic pH 2.9, flow rate 1.0 mL/min, detection wavelength at 249 nm, ambient temperature, 15 µL injection volume, thermo hypersil ODS C_18_ (4.6x250 mm, 5µm) column.

- **The variations made to the injection volume were (15** $\boldsymbol{\pm}$**2** $\boldsymbol{\mu}$**L)**

| 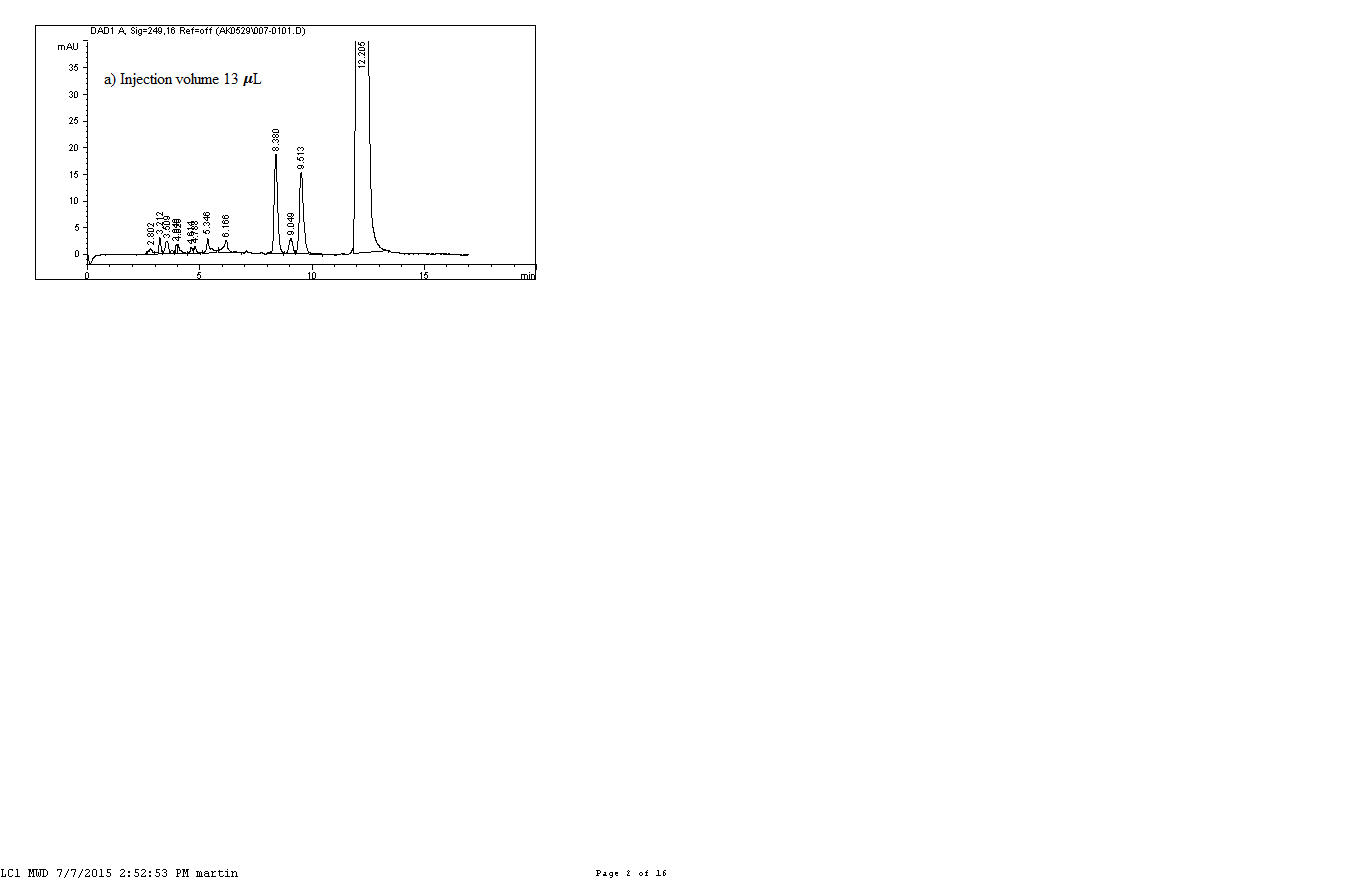 | 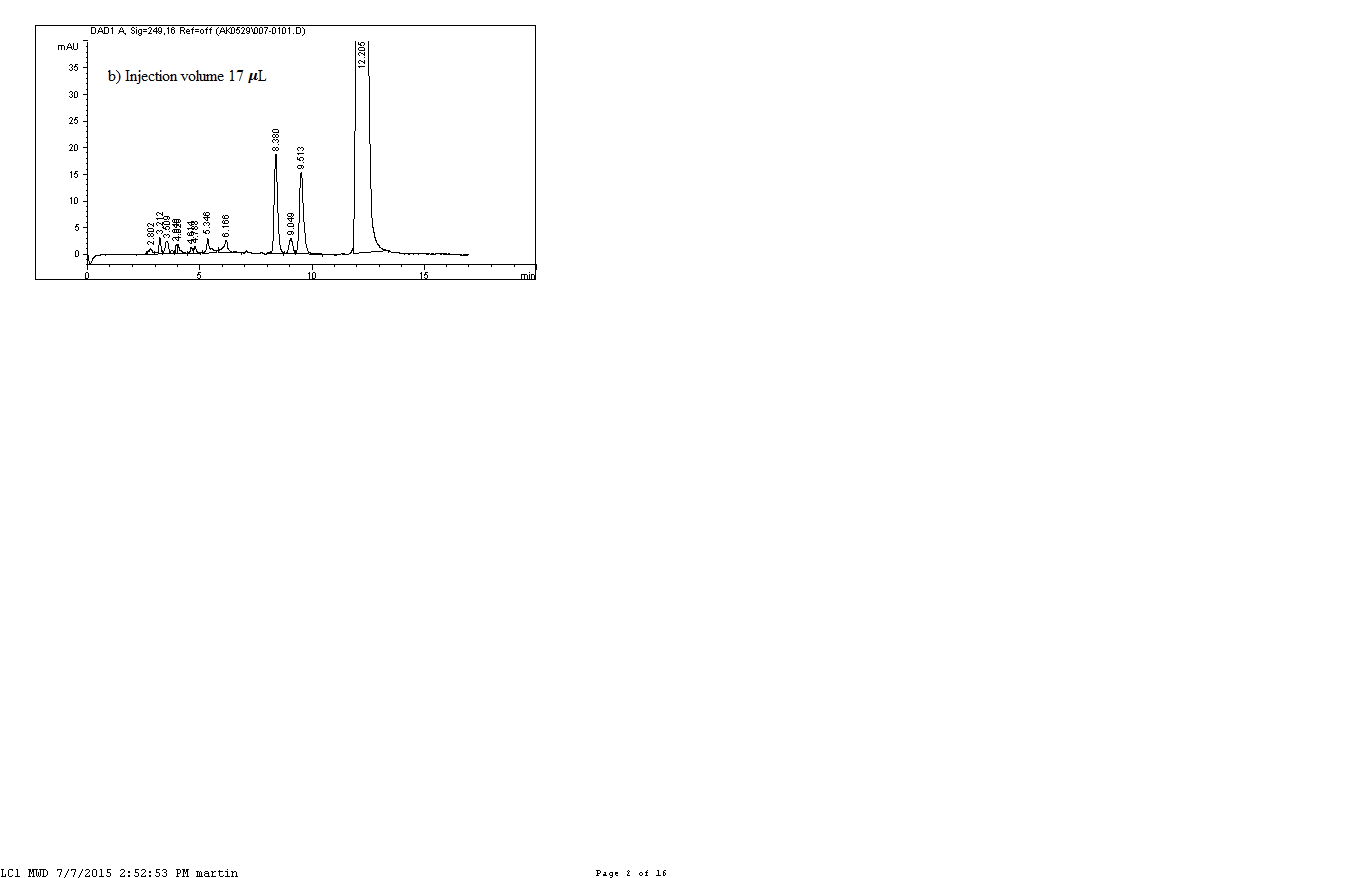 |
| --- | --- |
| 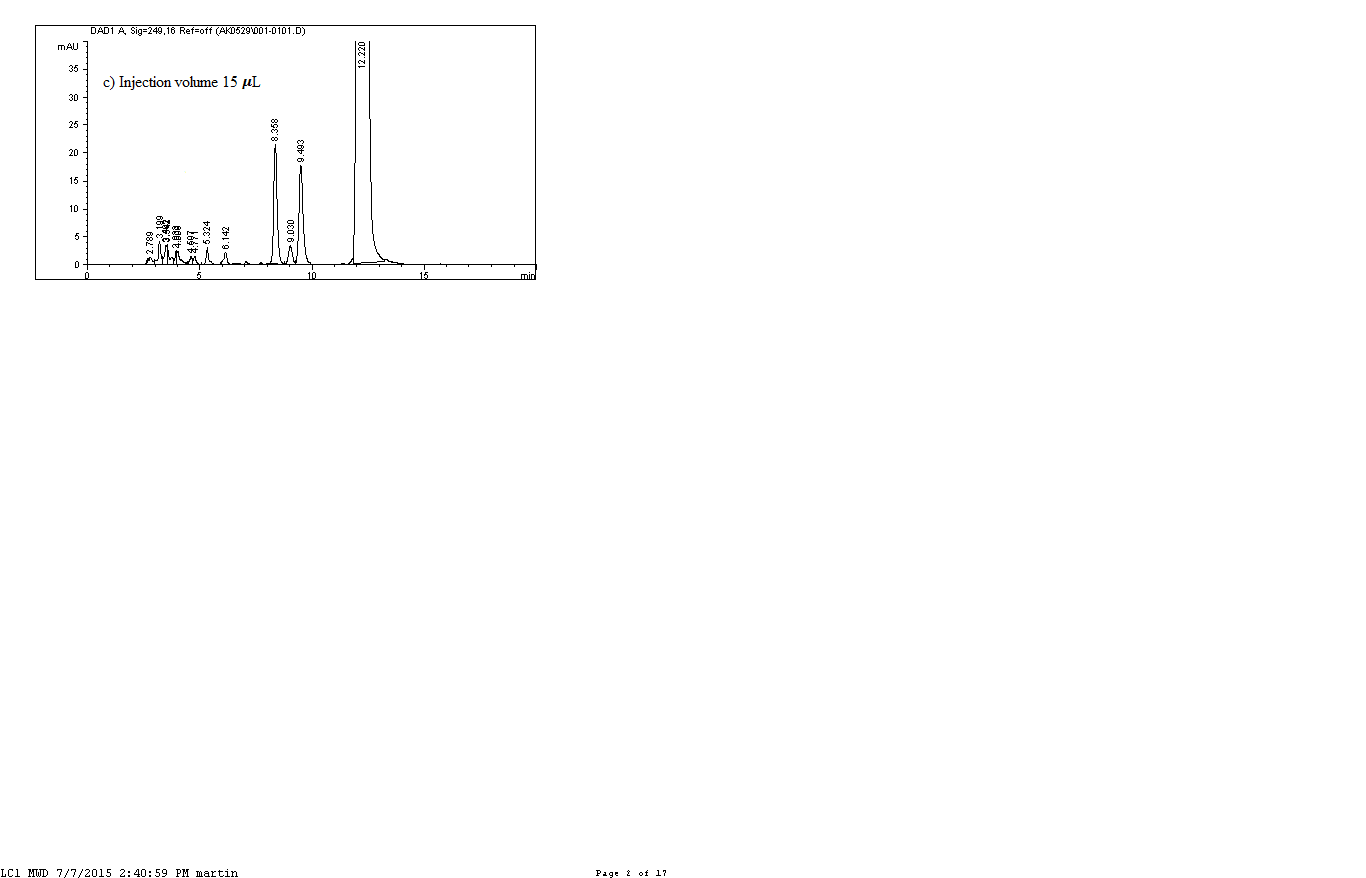 | |

**Figure S9.** Chromatograms of mixed degradation sample for injection volume robustness study.

1. Injection volume: 13µL c) Injection volume: 15µL (Developed Method)
2. Injection volume: 17µL

**Chromatographic conditions:** Isocratic elution, mobile phase 30:70 ACN/25 mM potassium phosphate buffer monobasic pH 2.9, flow rate 1.0 mL/min, detection wavelength at 249 nm, ambient temperature, thermo hypersil ODS C_18_ (4.6x250 mm, 5µm) column.

| **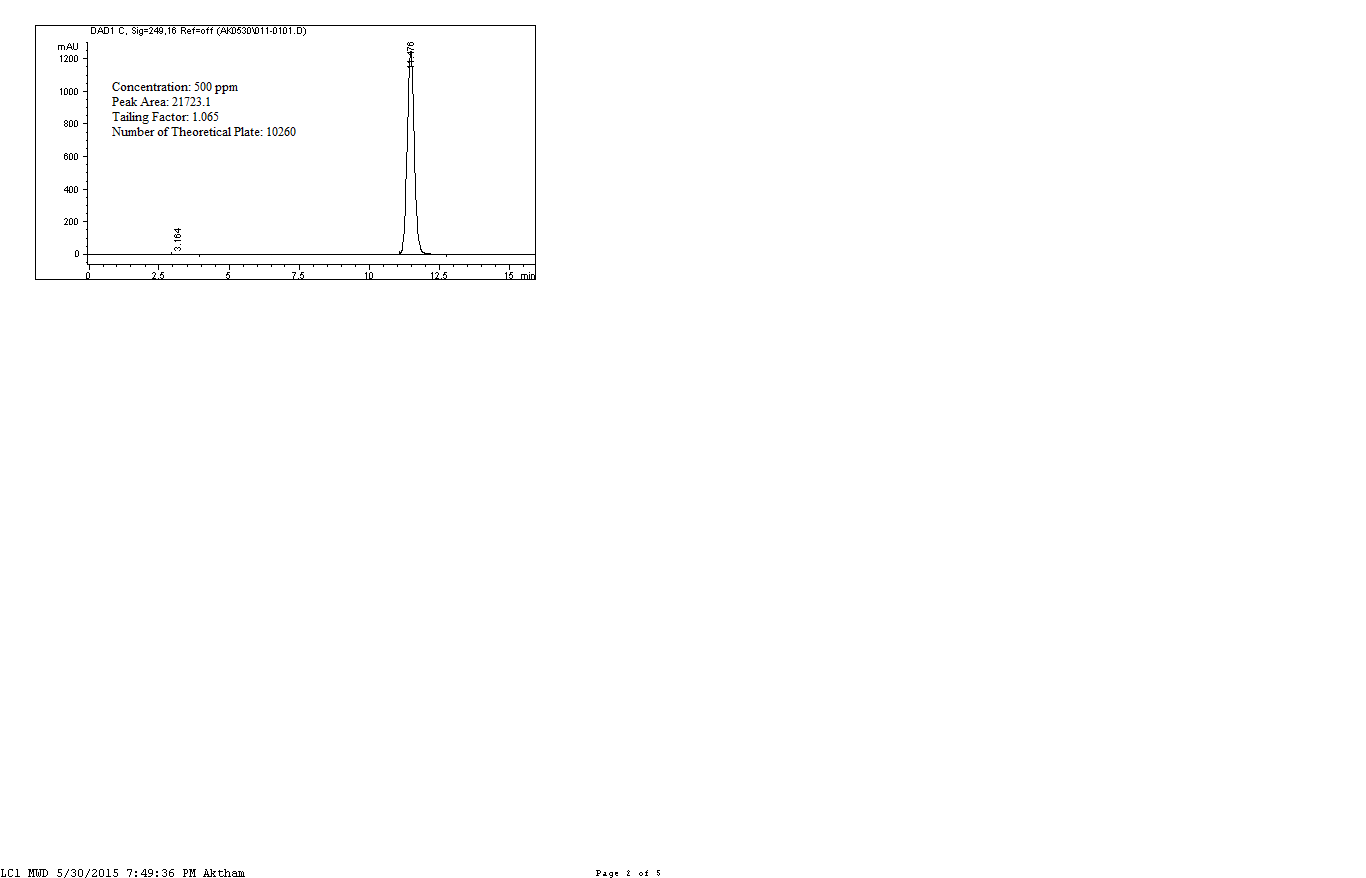** | **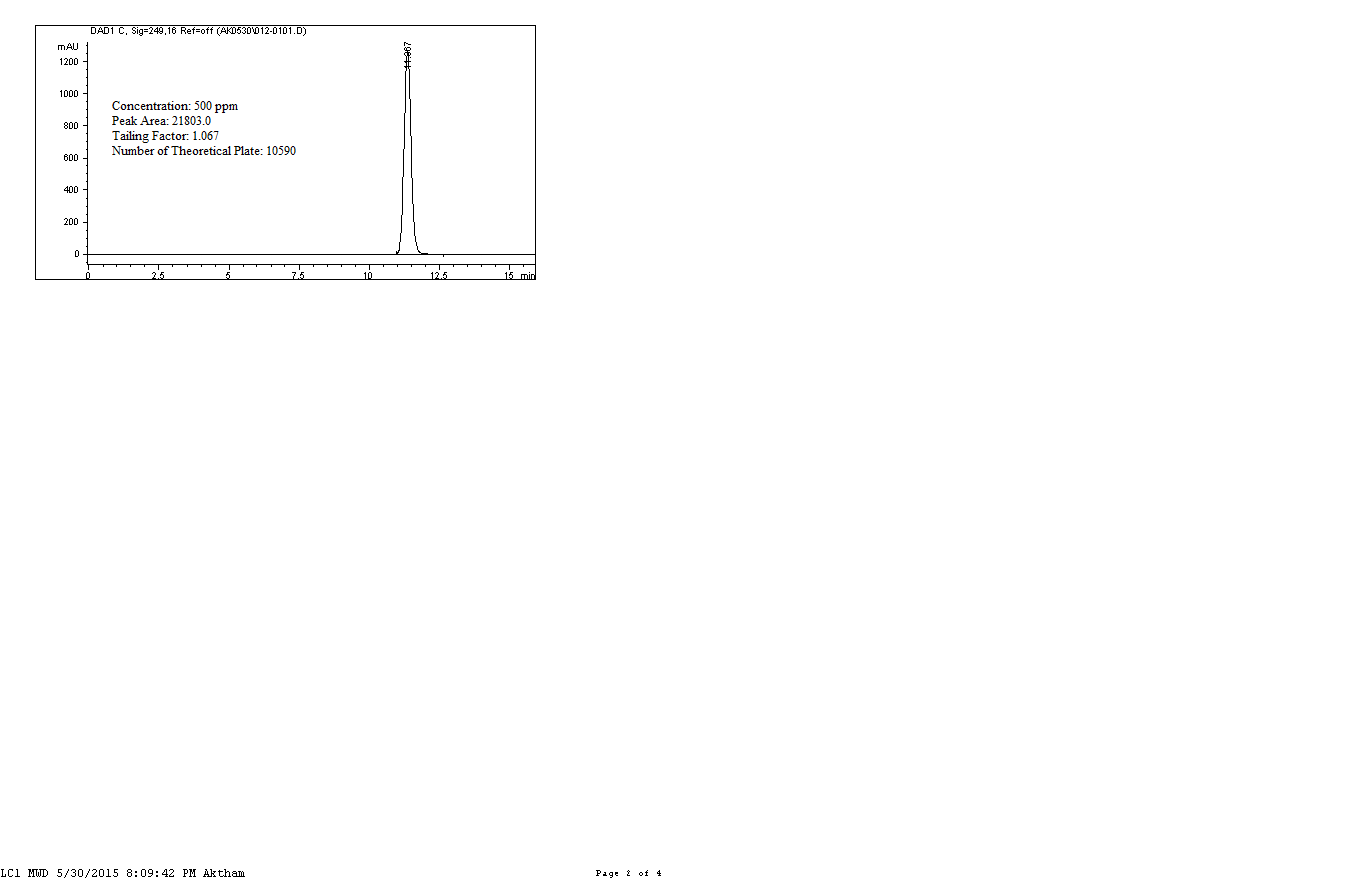** |
| --- | --- |
| **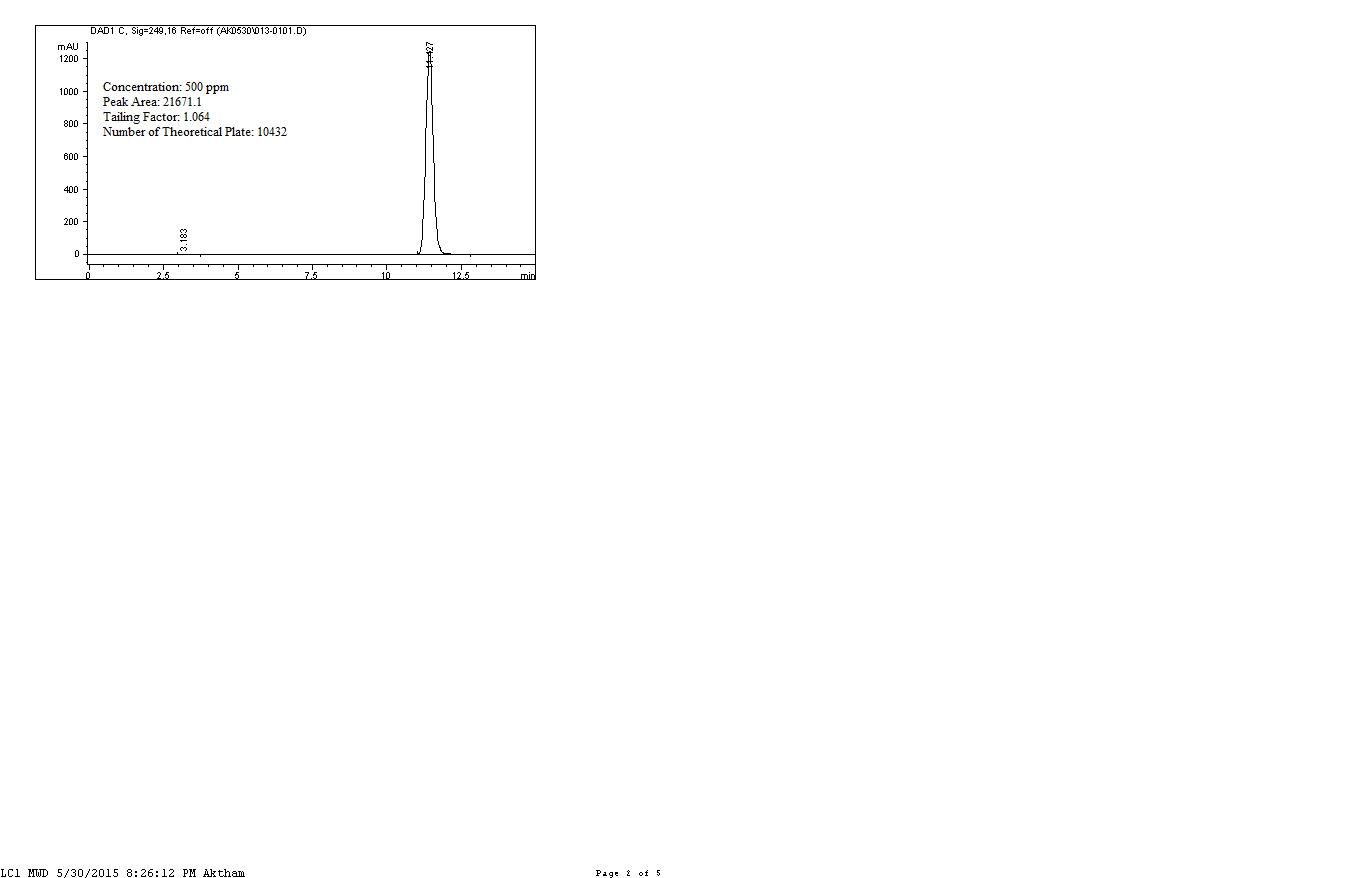** | |

**Figure S10.** Chromatograms for accuracy study for Rivaroxaban 500 ppm active ingredient

| **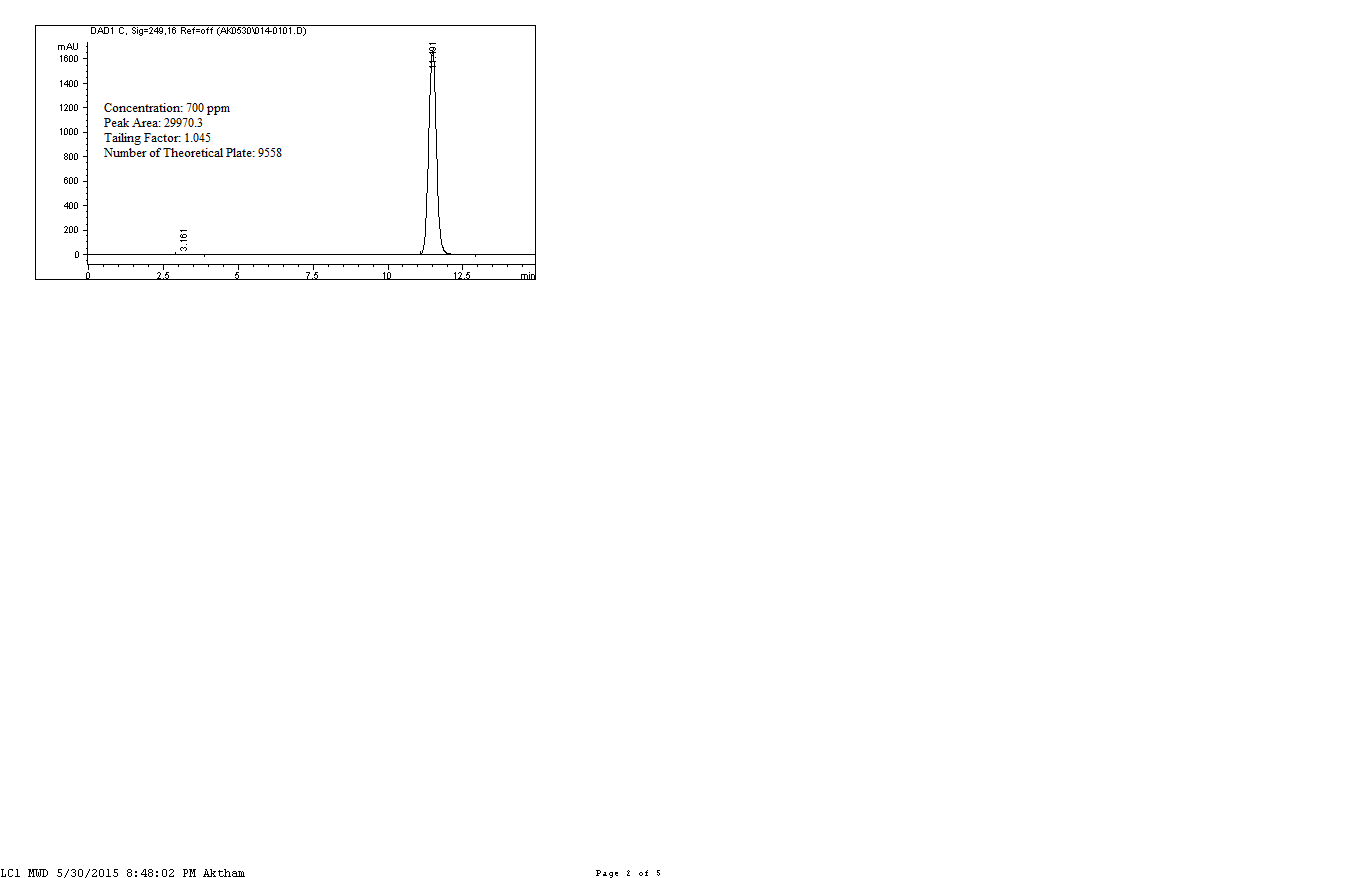** | **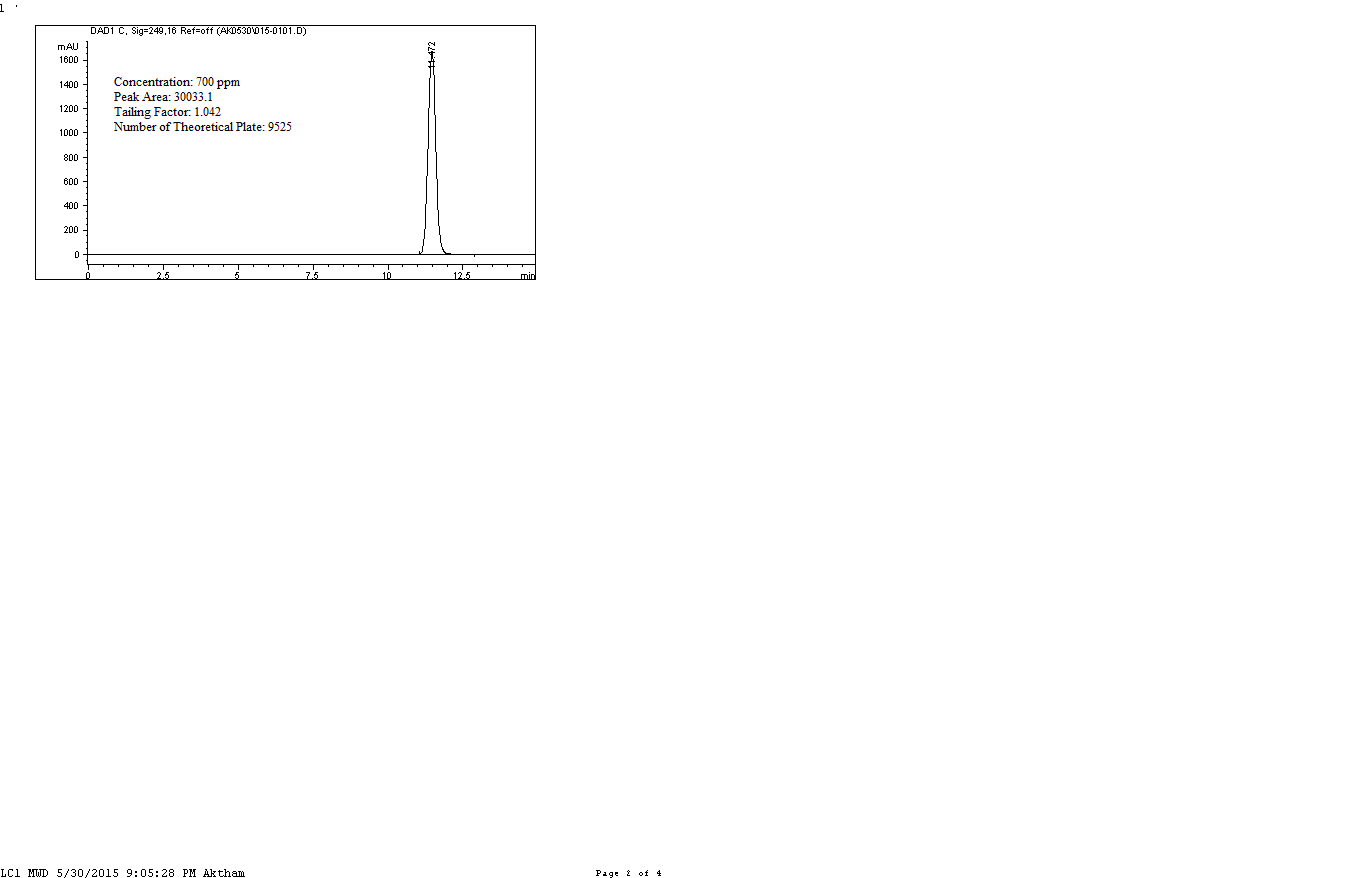** |
| --- | --- |
| **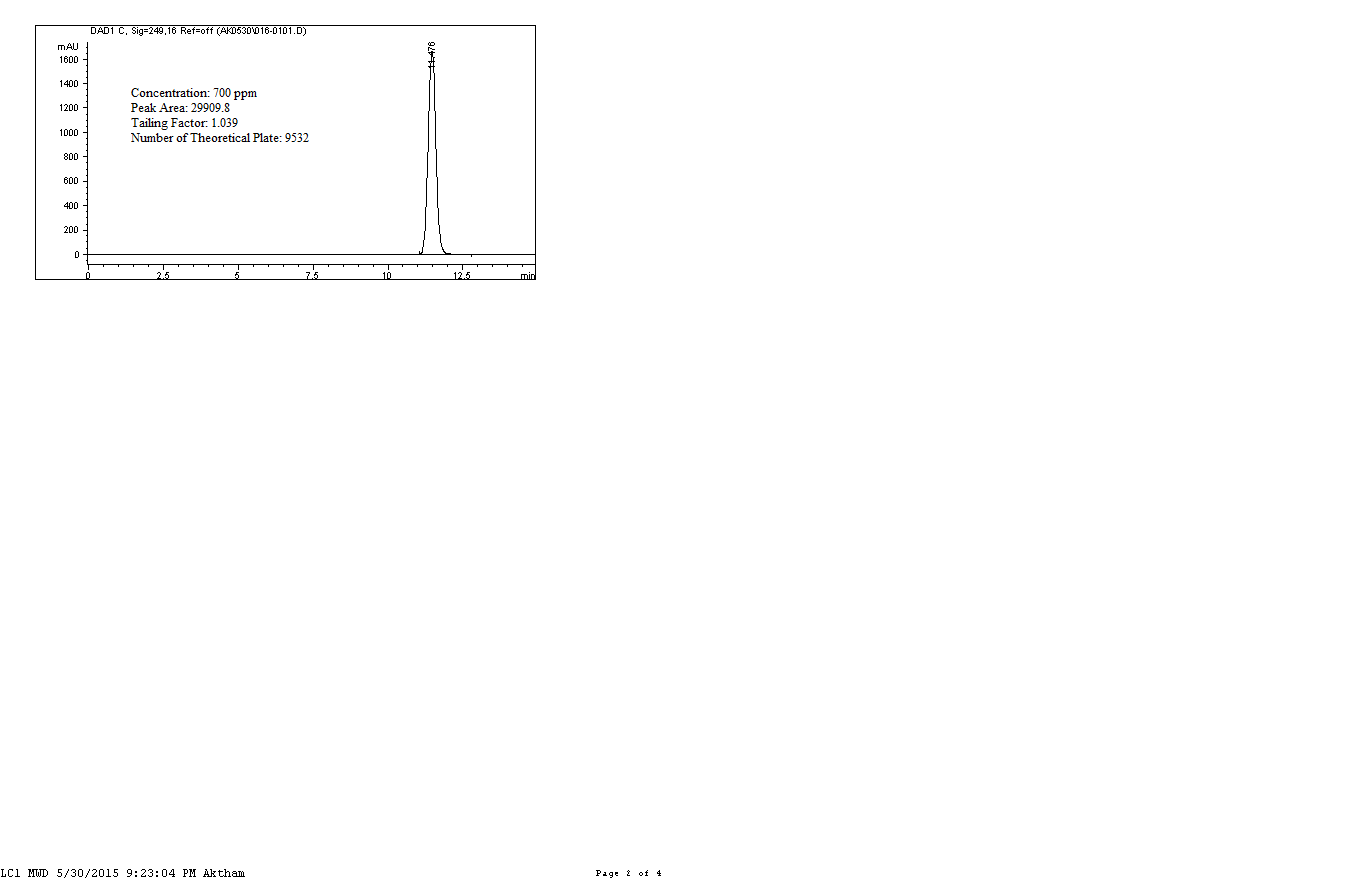** | |

**Figure S11.** Chromatograms for accuracy study for Rivaroxaban 700 ppm active ingredient

| **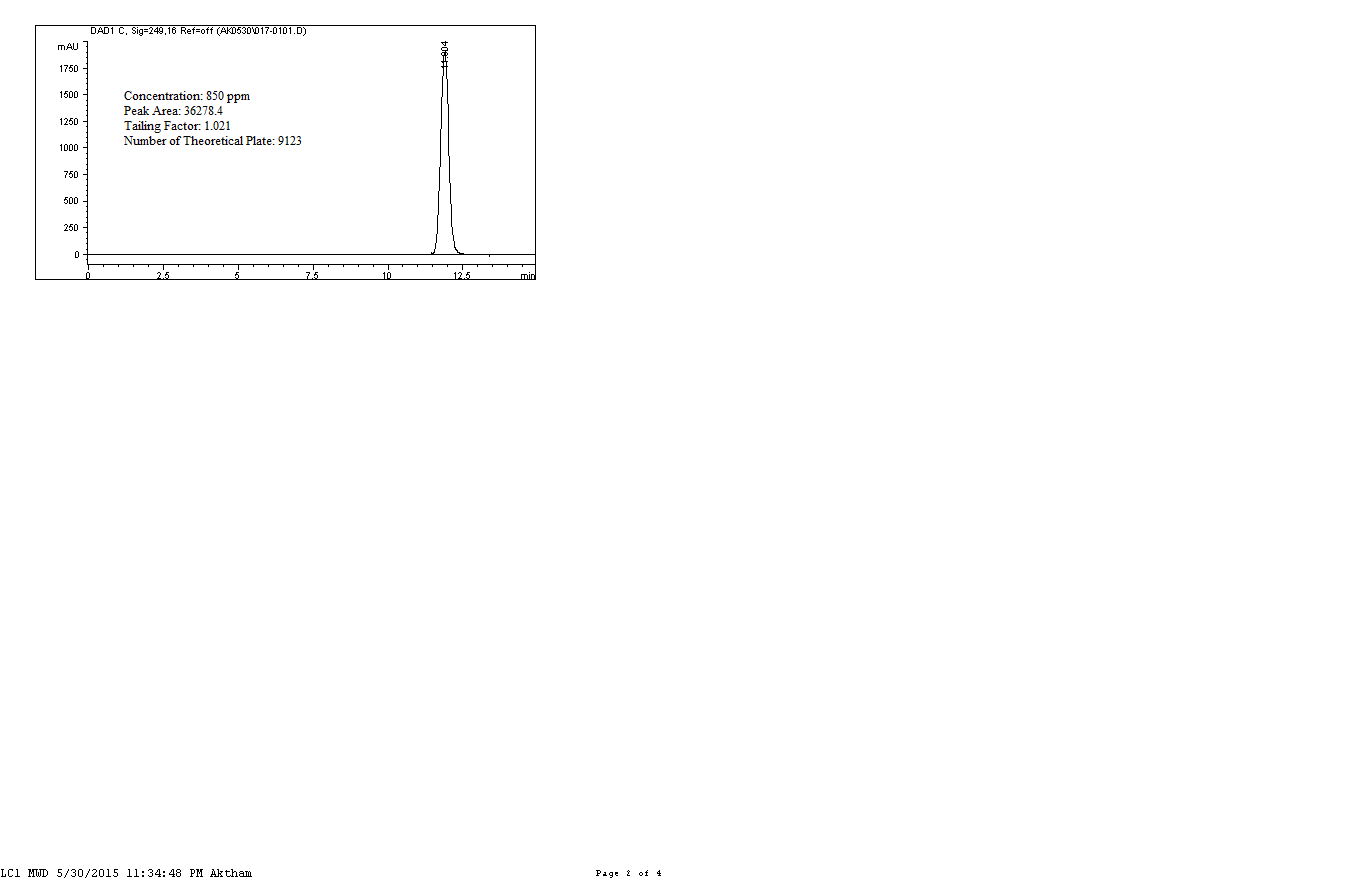** | **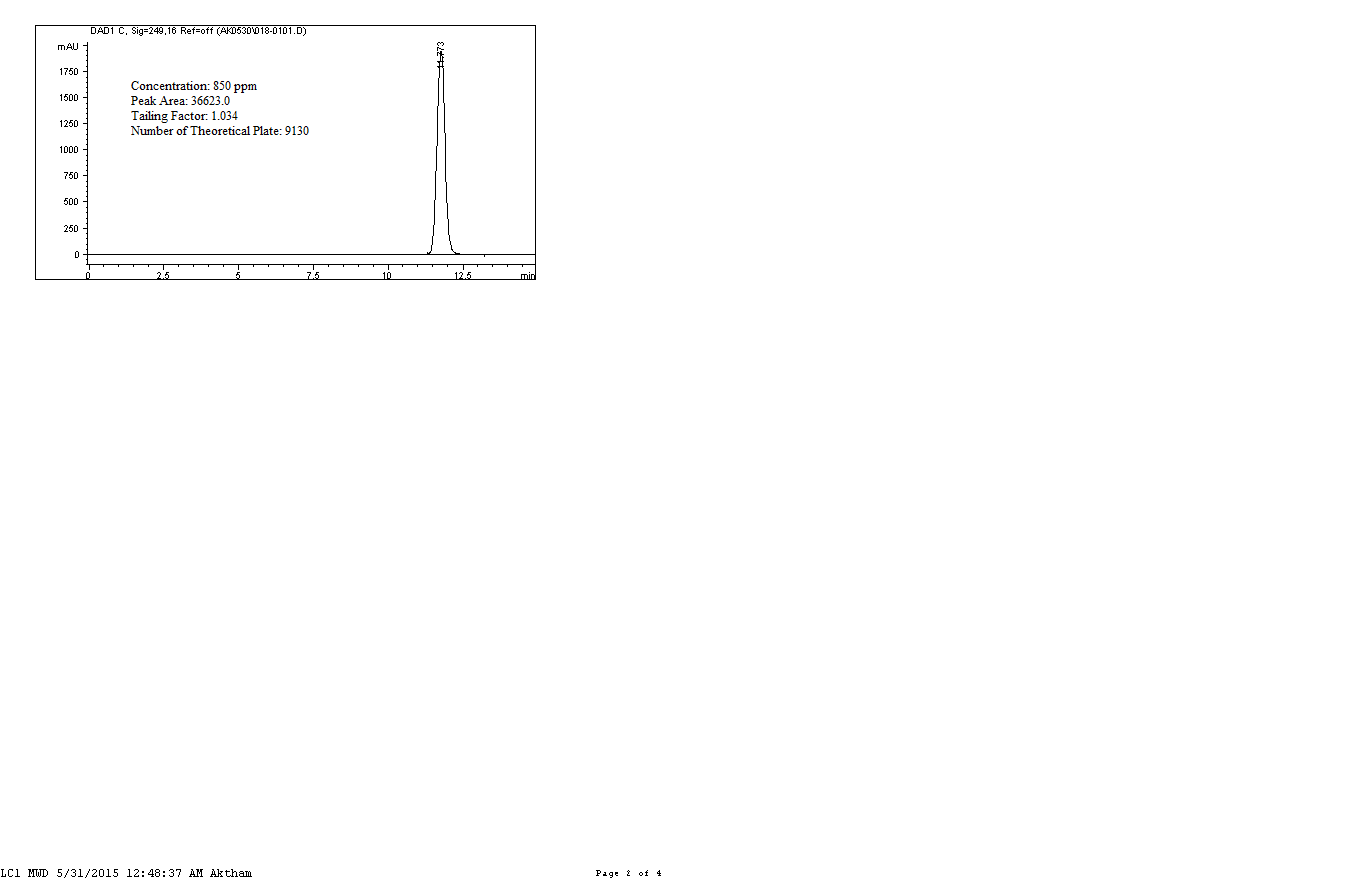** |
| --- | --- |
| **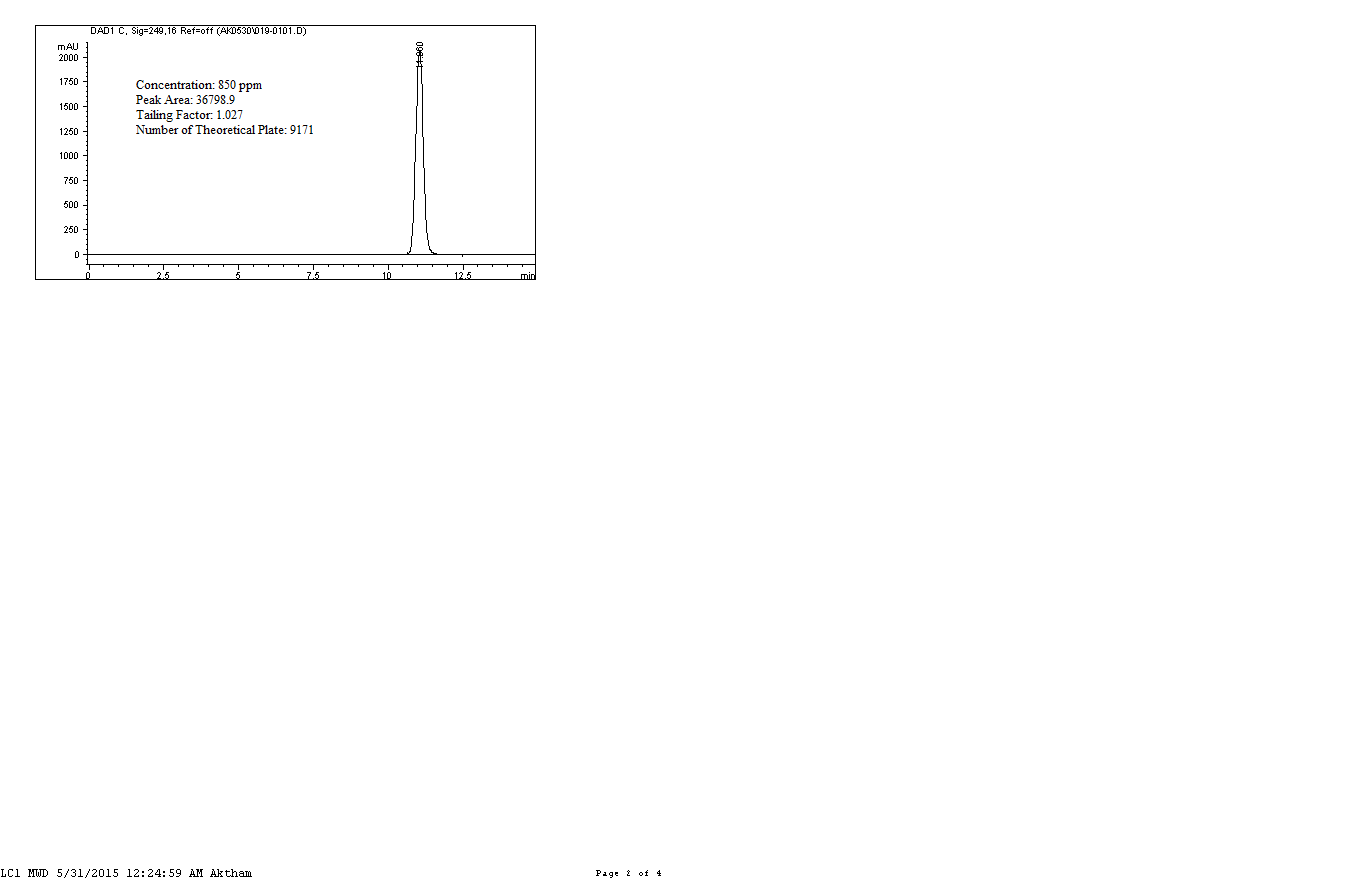** | |

**Figure S12.** Chromatograms for accuracy study for Rivaroxaban 850 ppm active ingredient

**Chromatographic conditions:** Isocratic elution, mobile phase 30:70 ACN/25 mM potassium phosphate buffer monobasic pH 2.9, flow rate 1.0 mL/min, detection wavelength at 249 nm, ambient temperature, 15 µL injection volume, thermo hypersil ODS C_18_ (4.6x250 mm, 5µm) column.

| **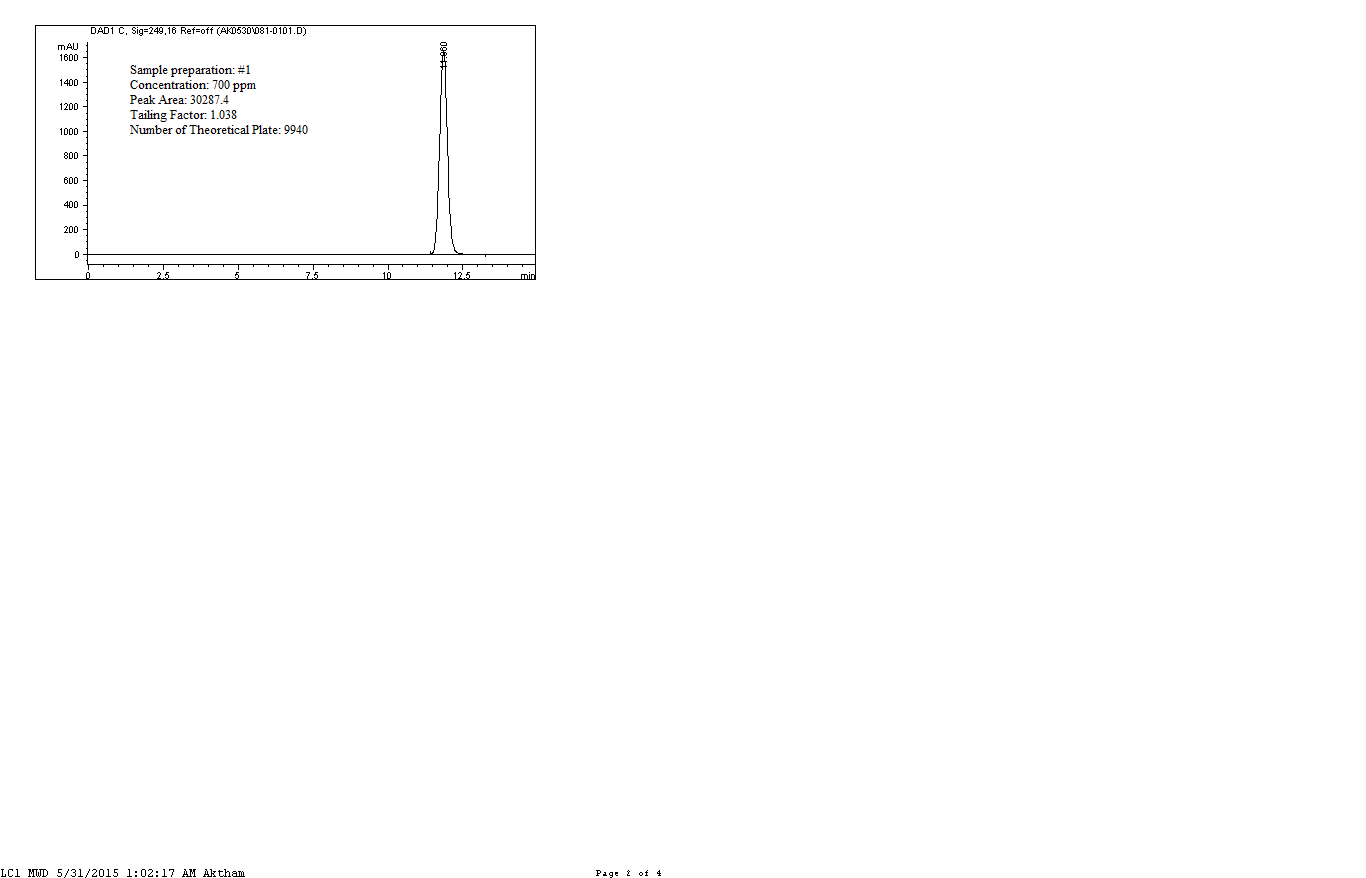** | **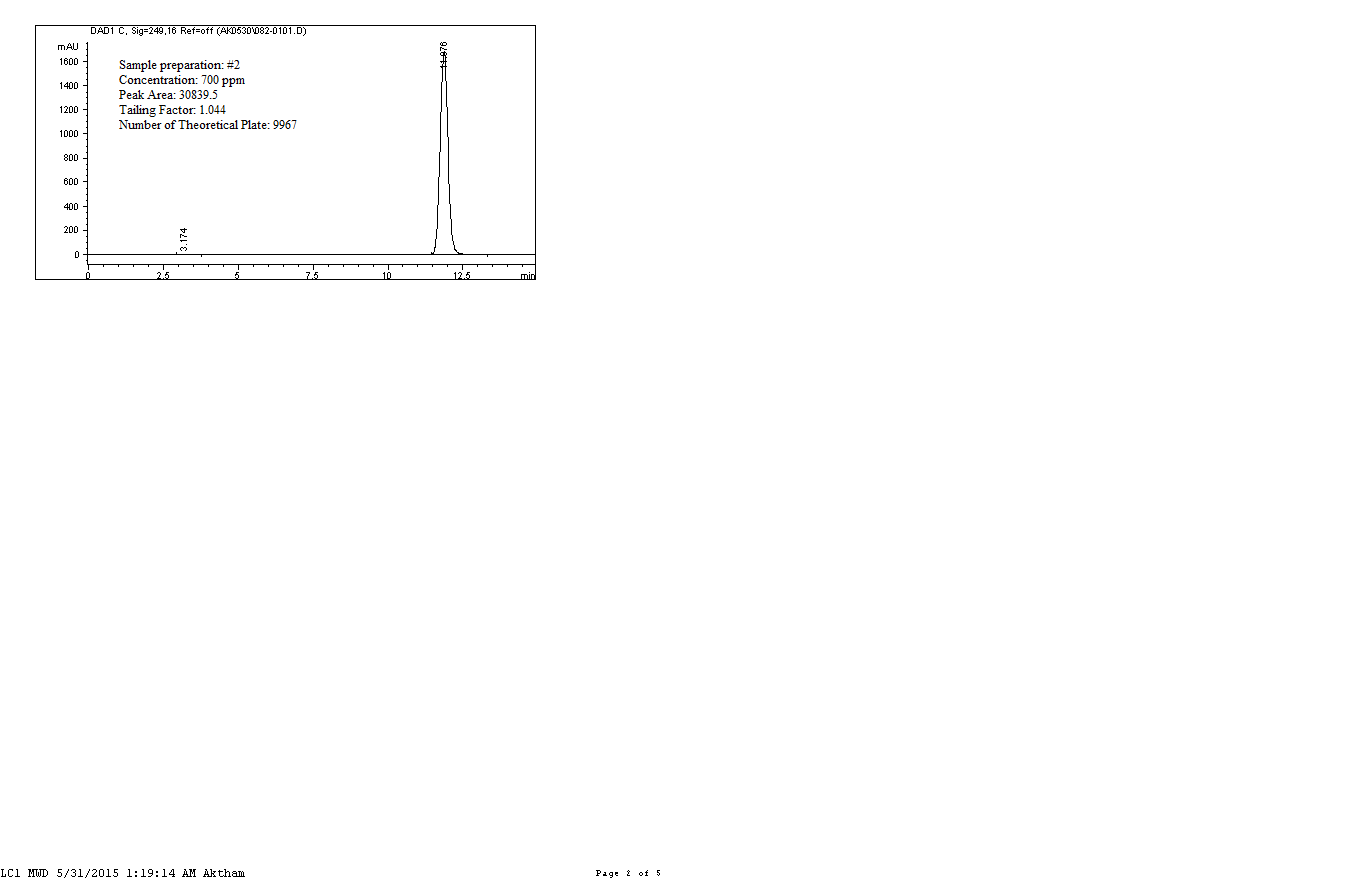** |
| --- | --- |
| **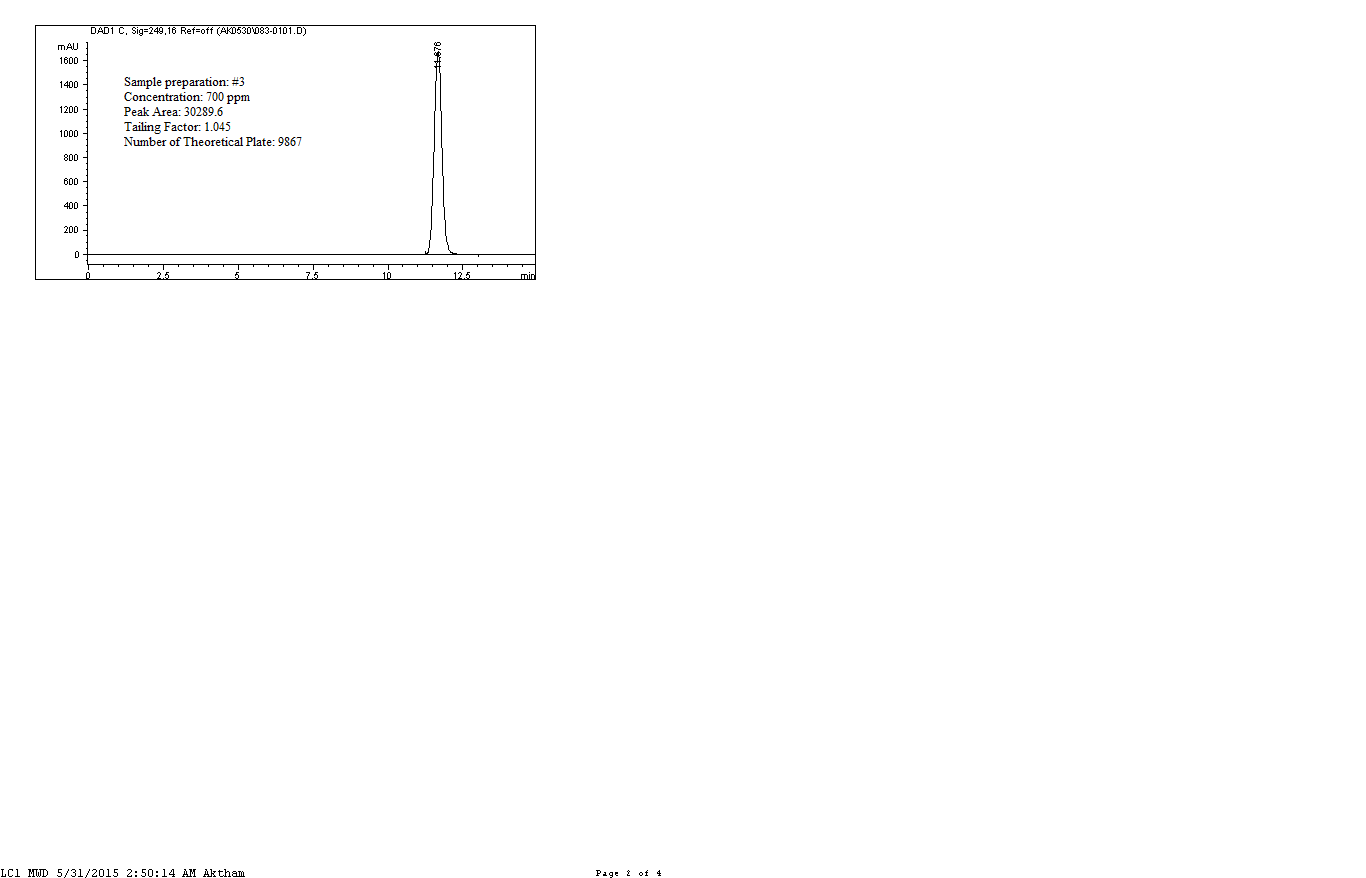** | **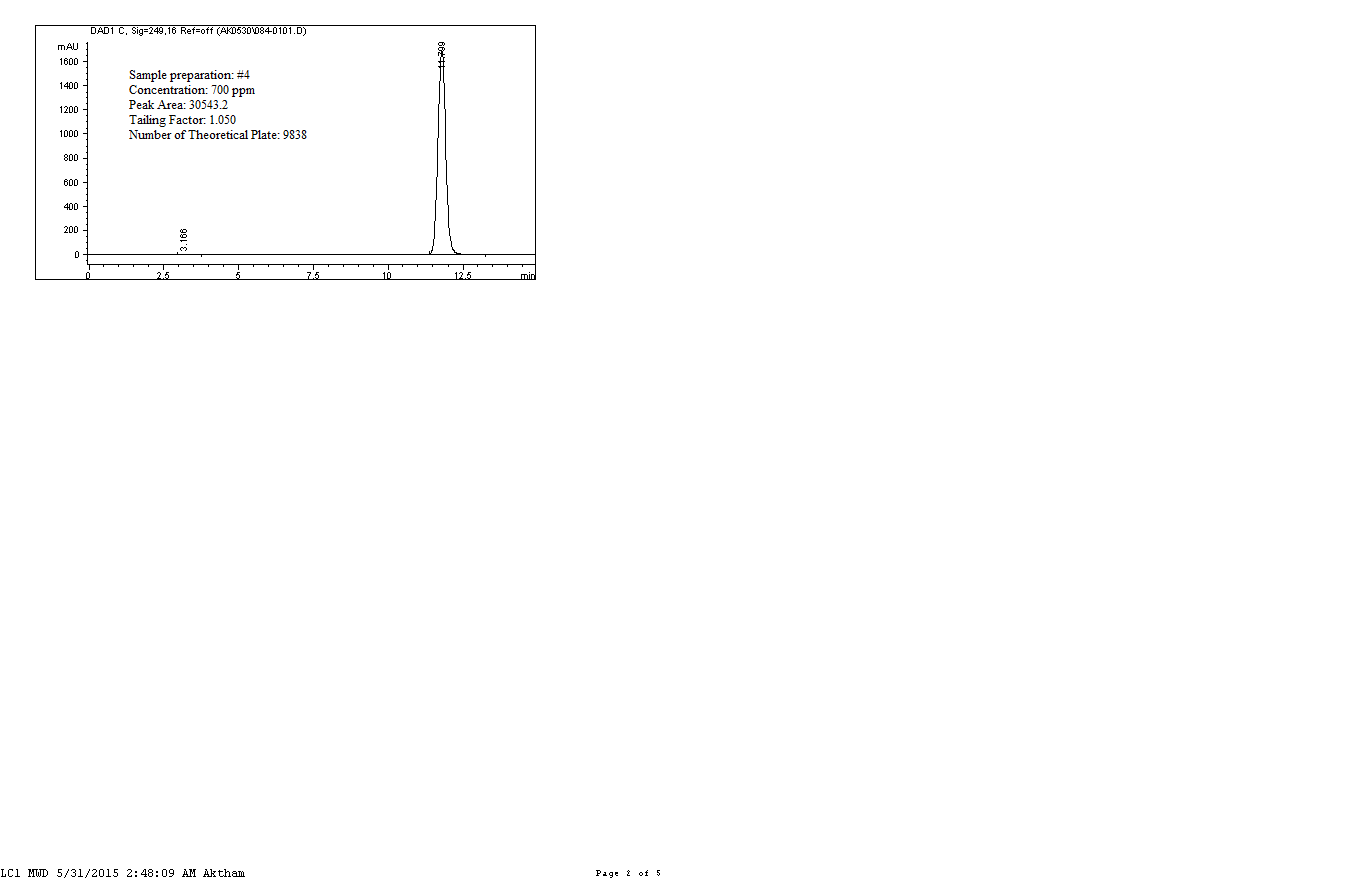** |
| **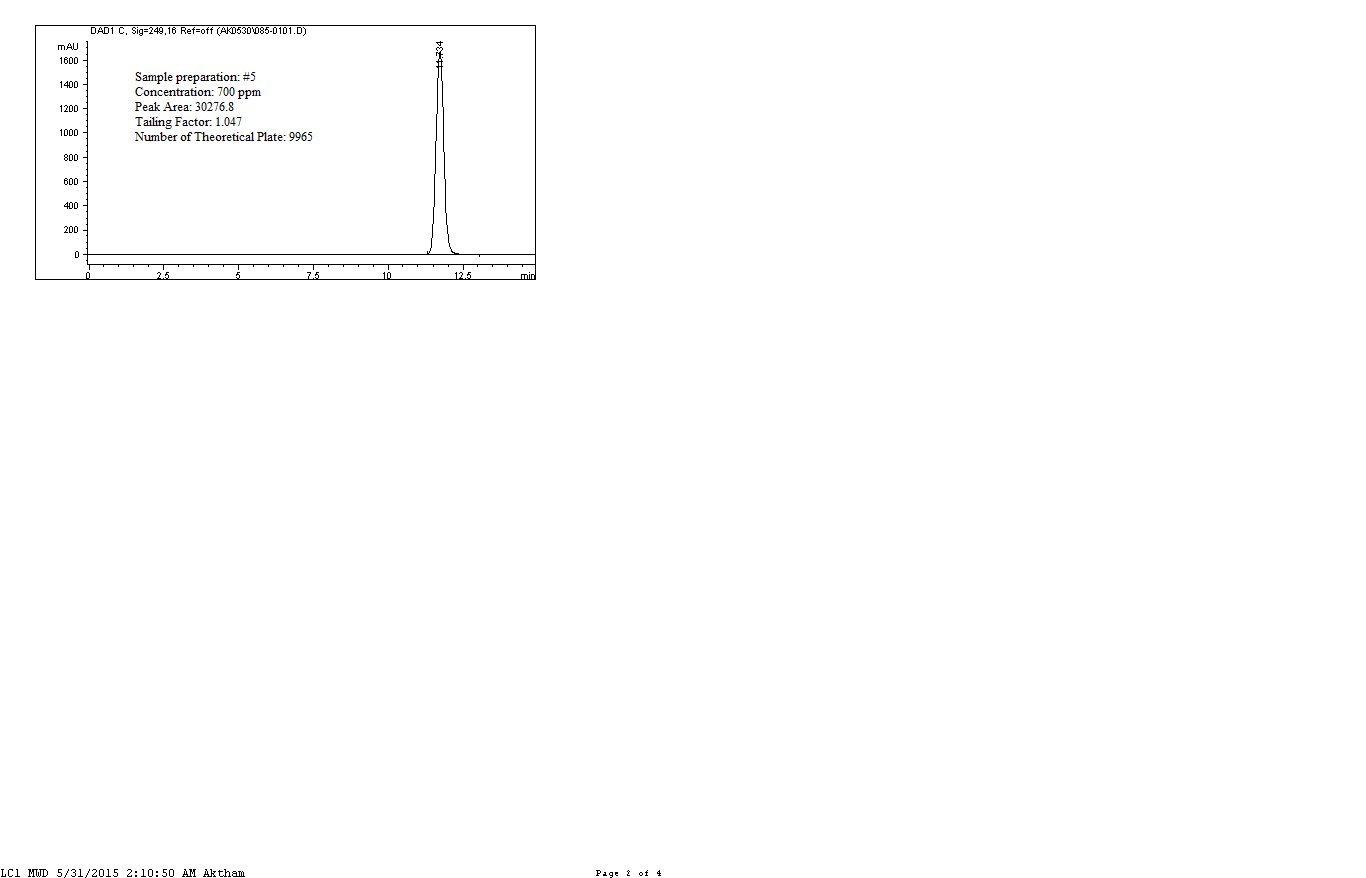** | **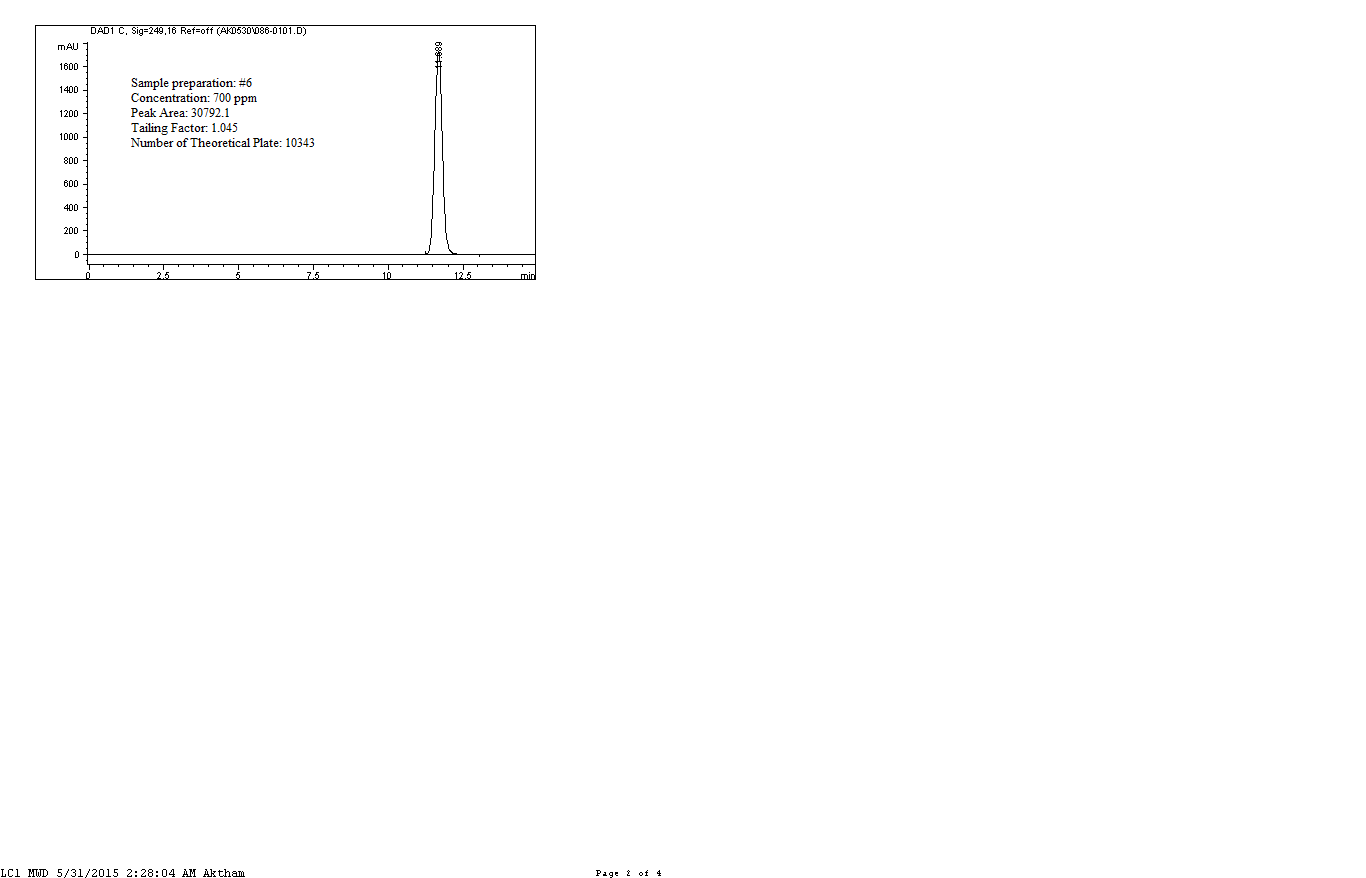** |

**Figure S13.** Chromatograms for method precision study for Rivaroxaban 700 ppm active ingredient

**Chromatographic conditions:** Isocratic elution, mobile phase 30:70 ACN/25 mM potassium phosphate buffer monobasic pH 2.9, flow rate 1.0 mL/min, detection wavelength at 249 nm, ambient temperature, 15 µL injection volume, thermo hypersil ODS C_18_ (4.6x250 mm, 5µm) column.

| 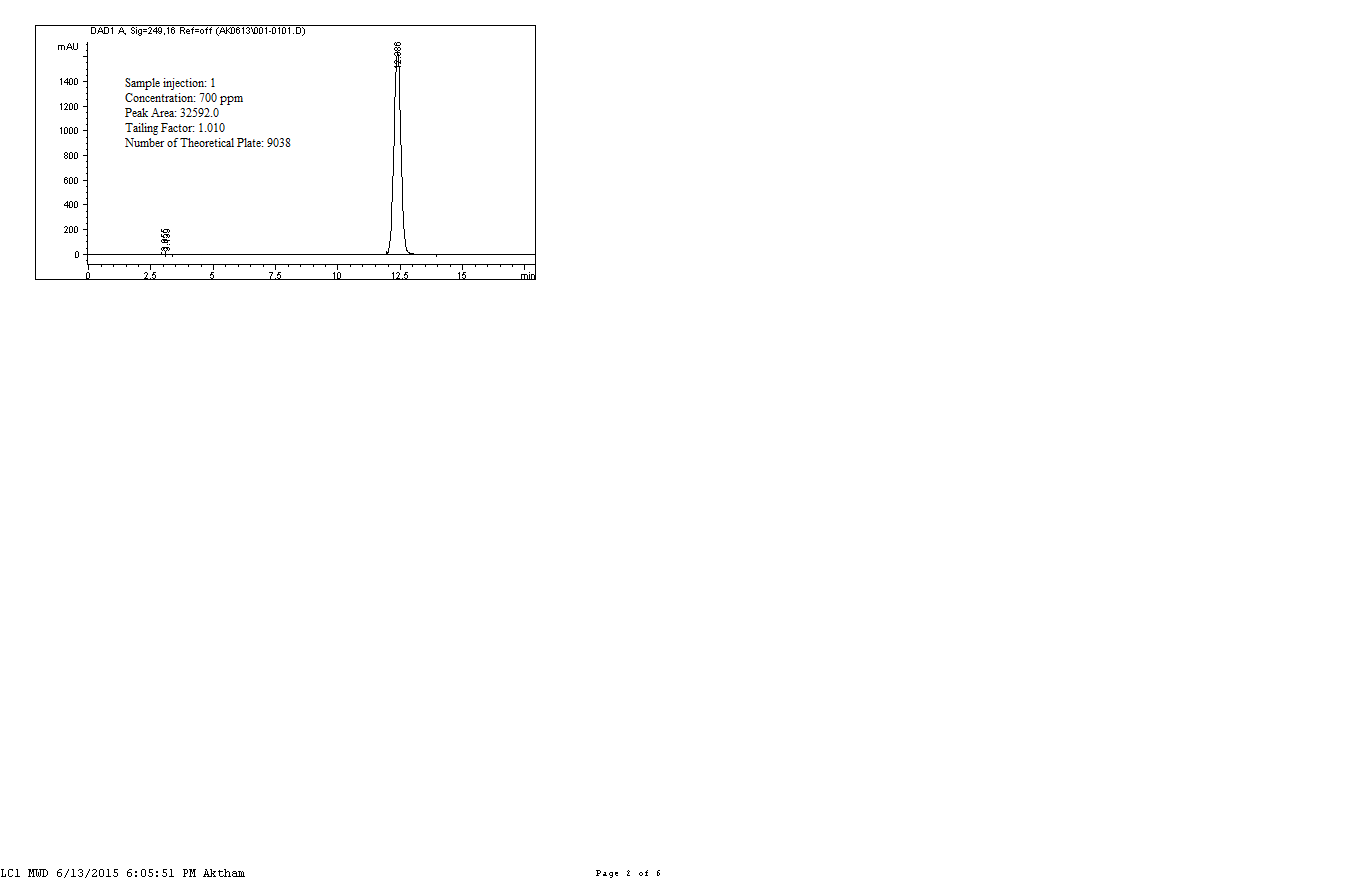 | 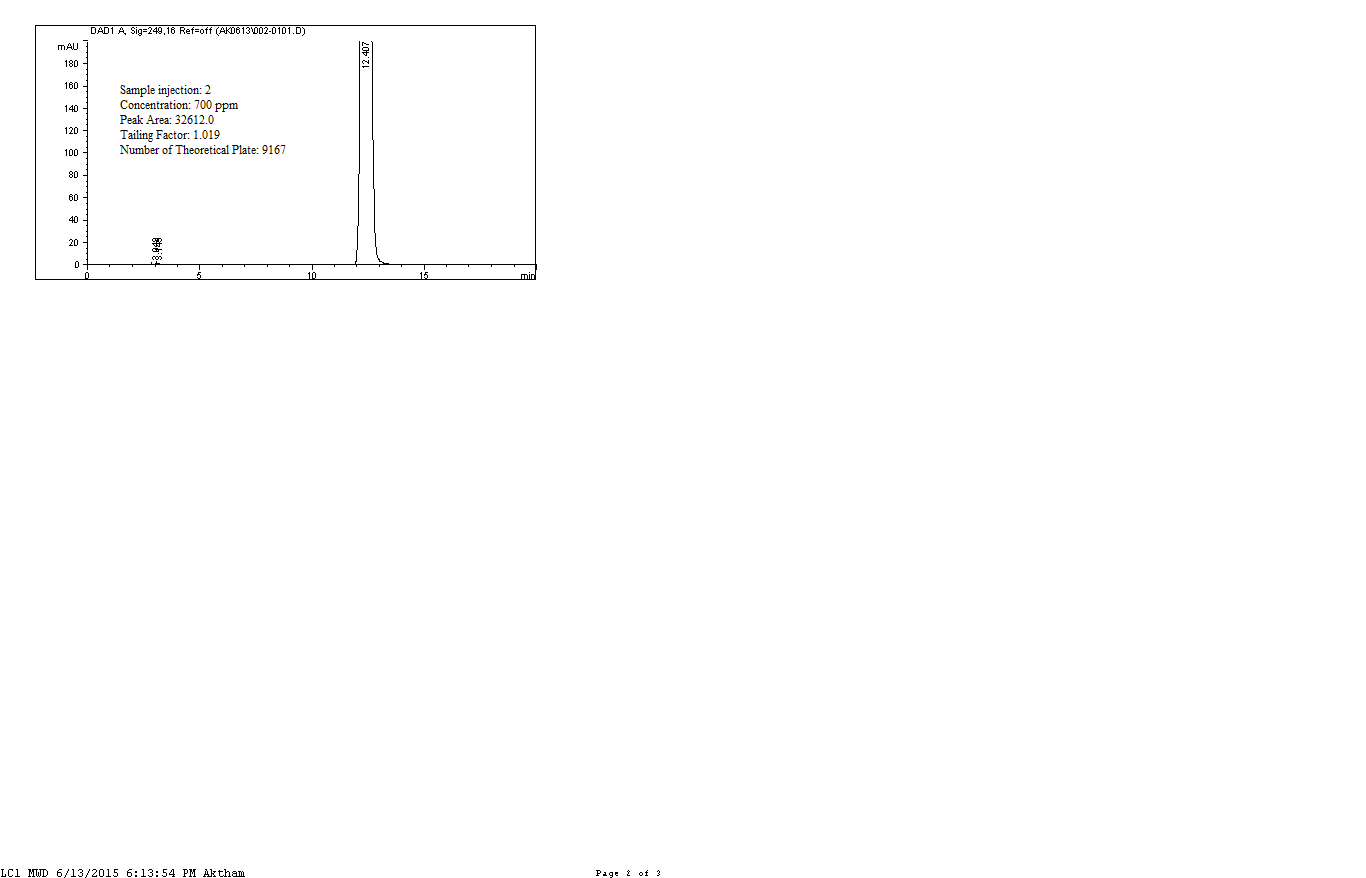 |
| --- | --- |
| 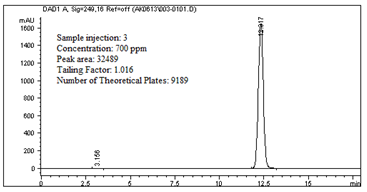 | 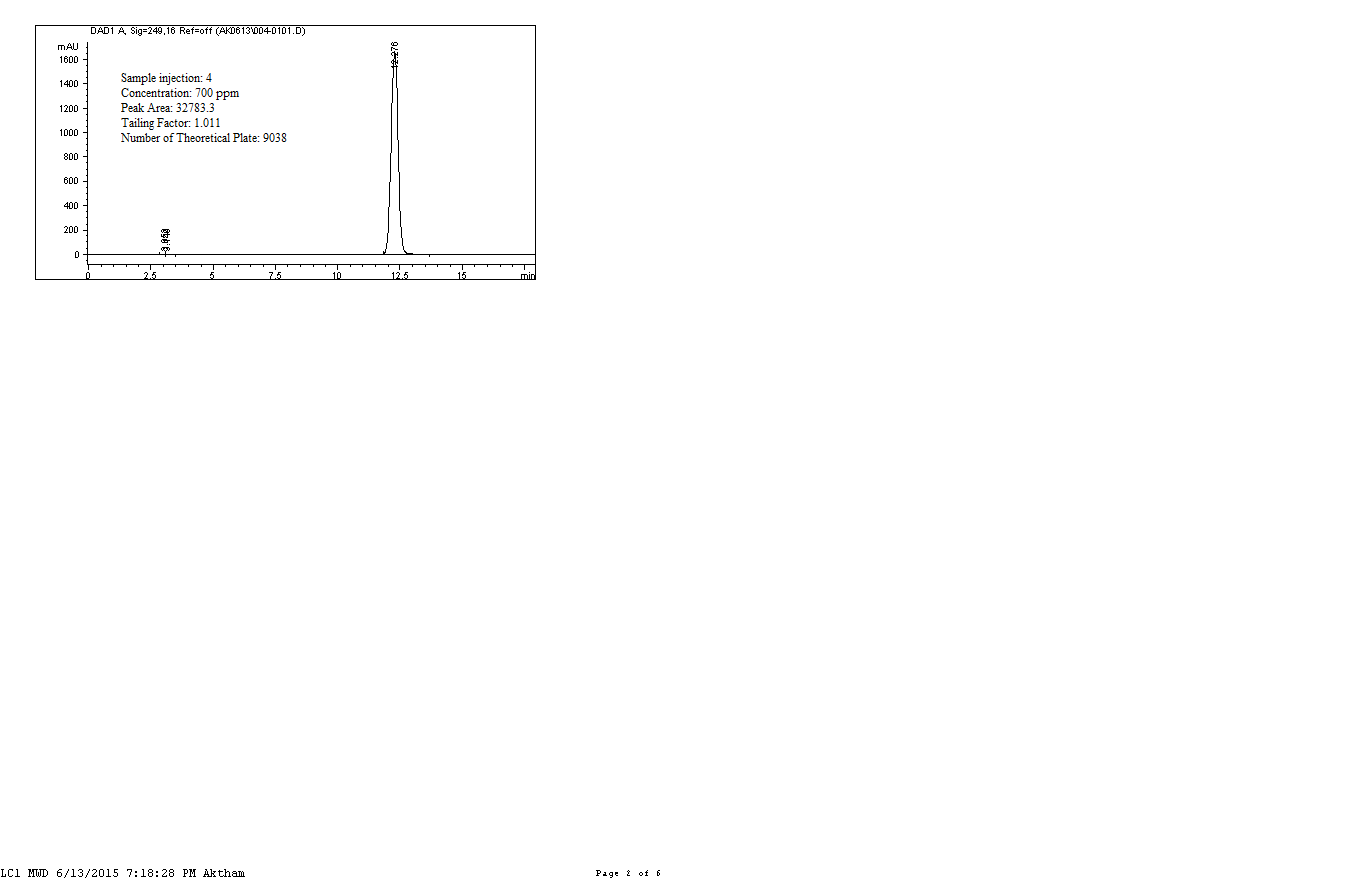 |
| 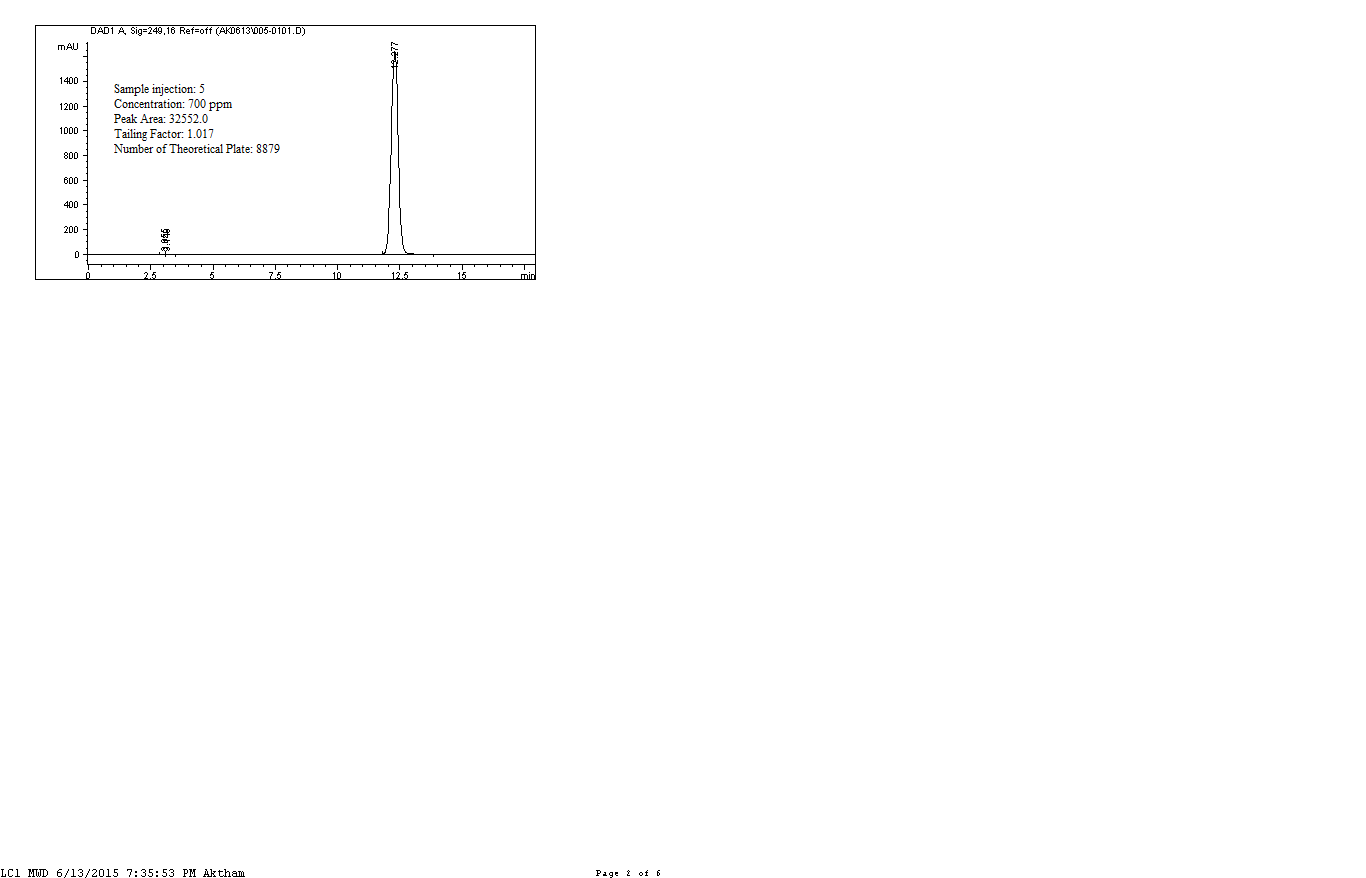 | 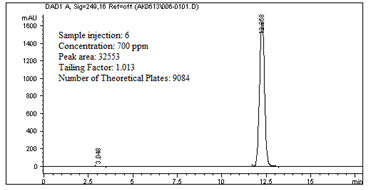 |

**Figure S14.** Chromatograms for injection precision for Rivaroxaban 700 ppm active ingredient

**Chromatographic conditions:** Isocratic elution, mobile phase 30:70 ACN/25 mM potassium phosphate buffer monobasic pH 2.9, flow rate 1.0 mL/min, detection wavelength at 249 nm, ambient temperature, 15 µL injection volume, thermo hypersil ODS C_18_ (4.6x250 mm, 5µm) column.

| **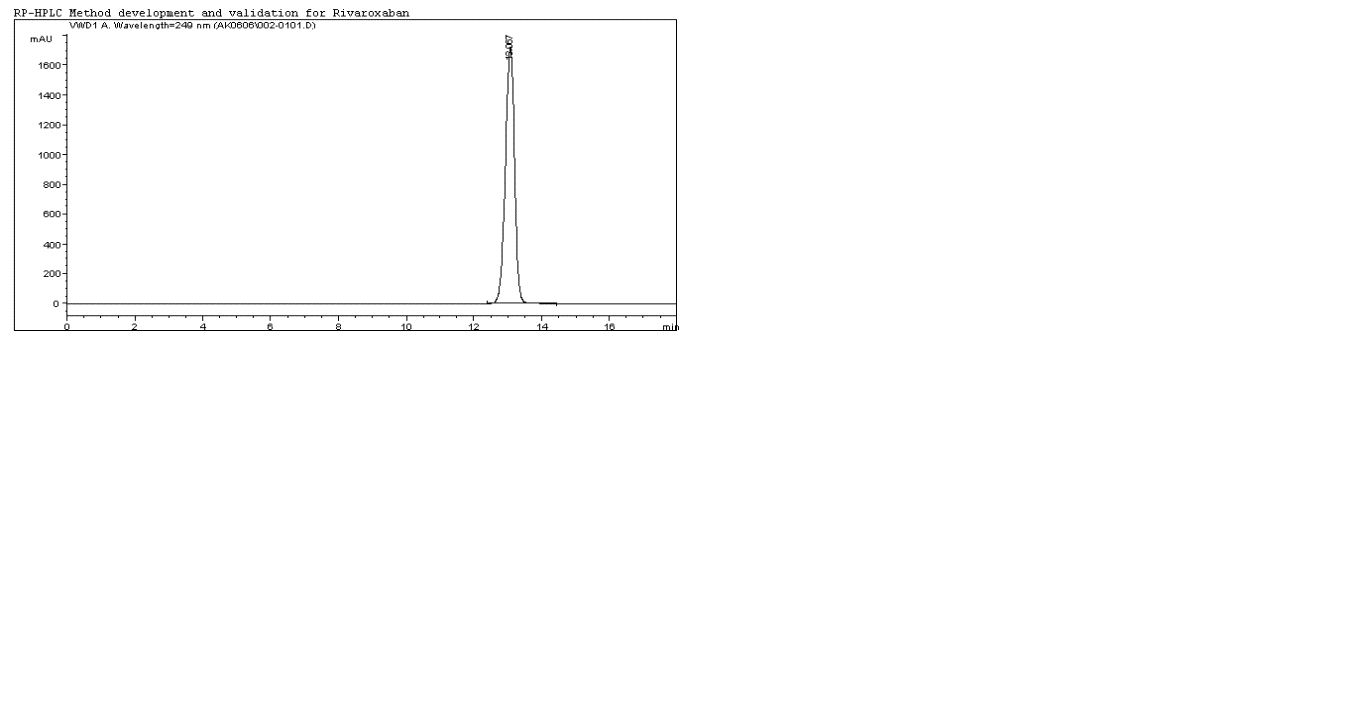** | **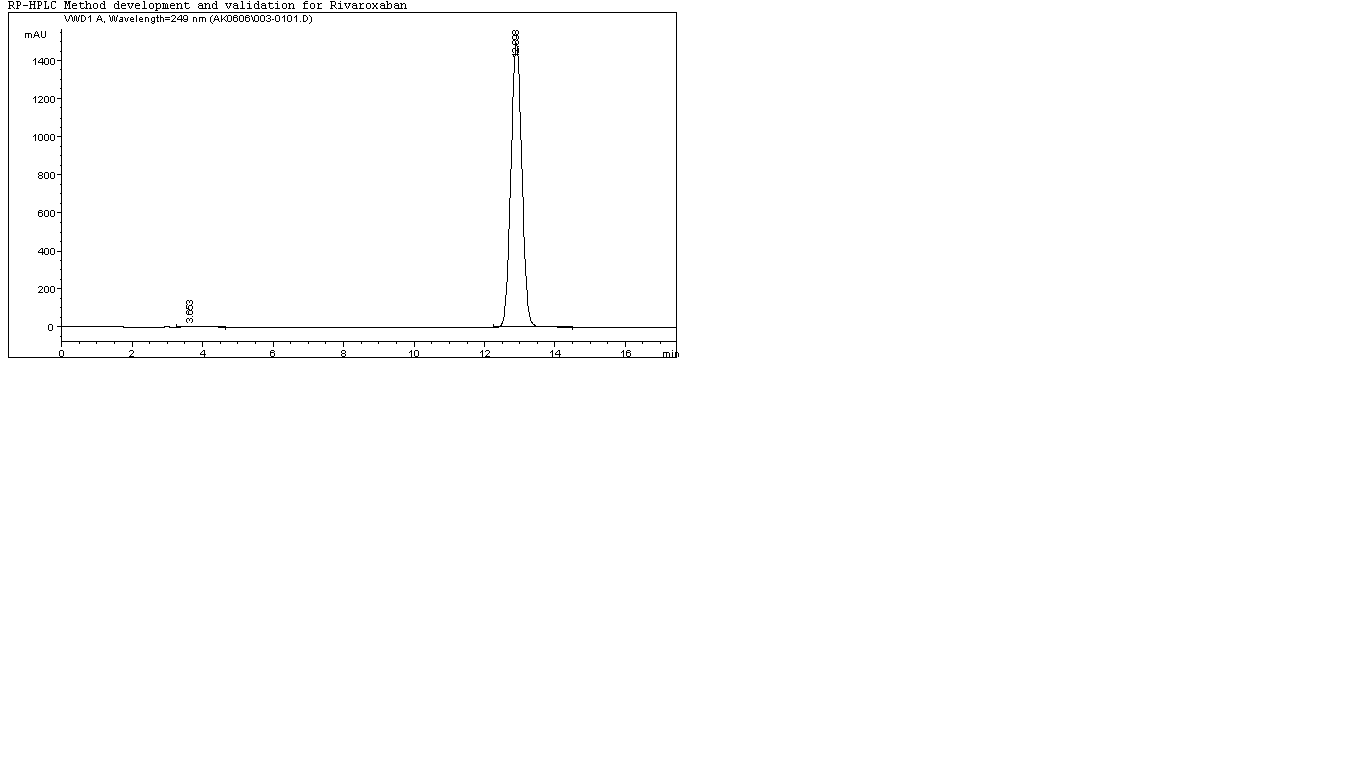** |
| --- | --- |
| **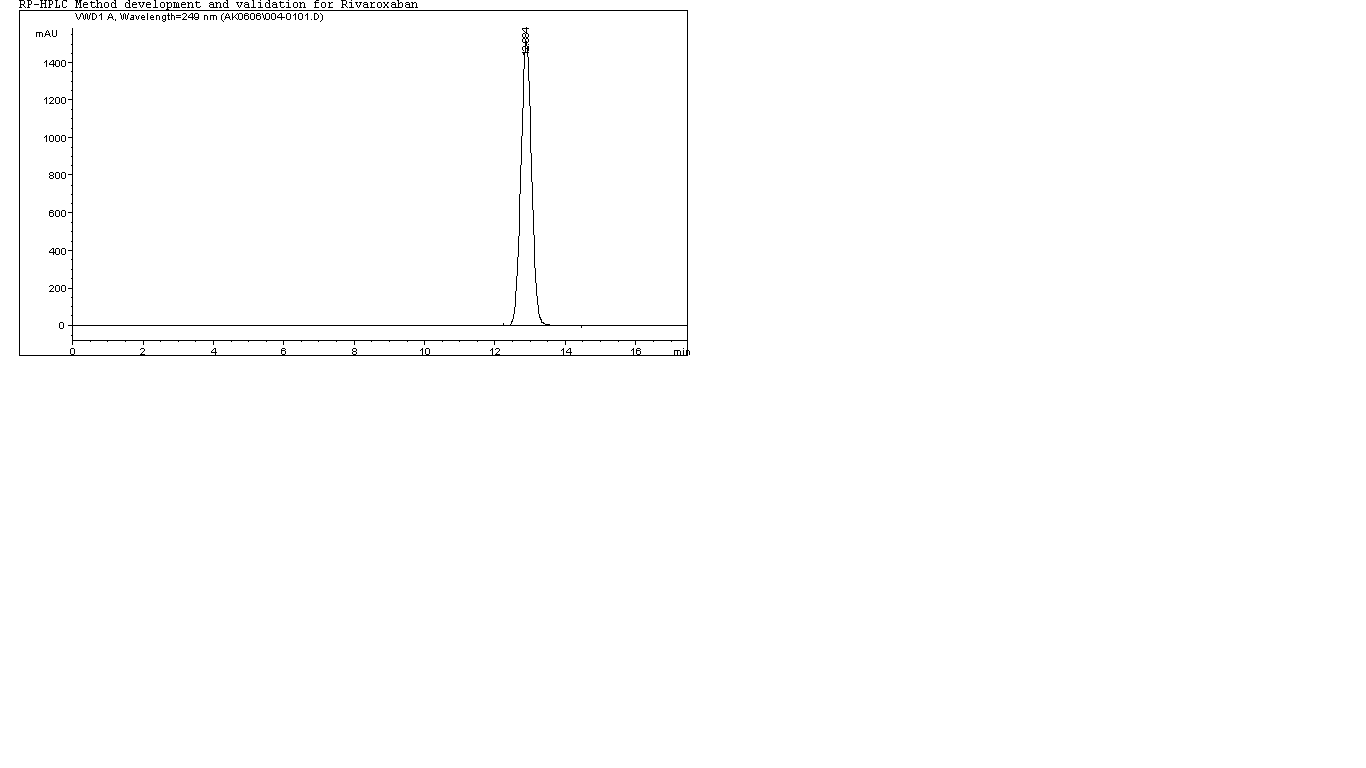** | **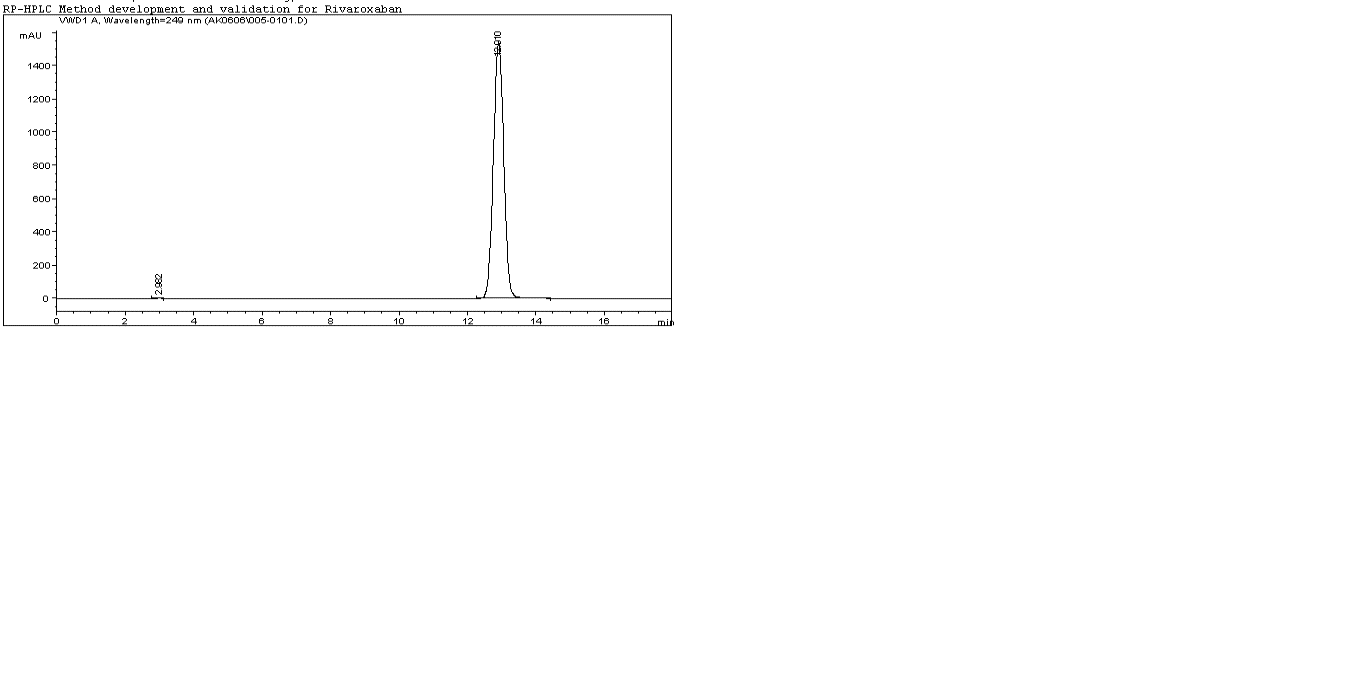** |
| **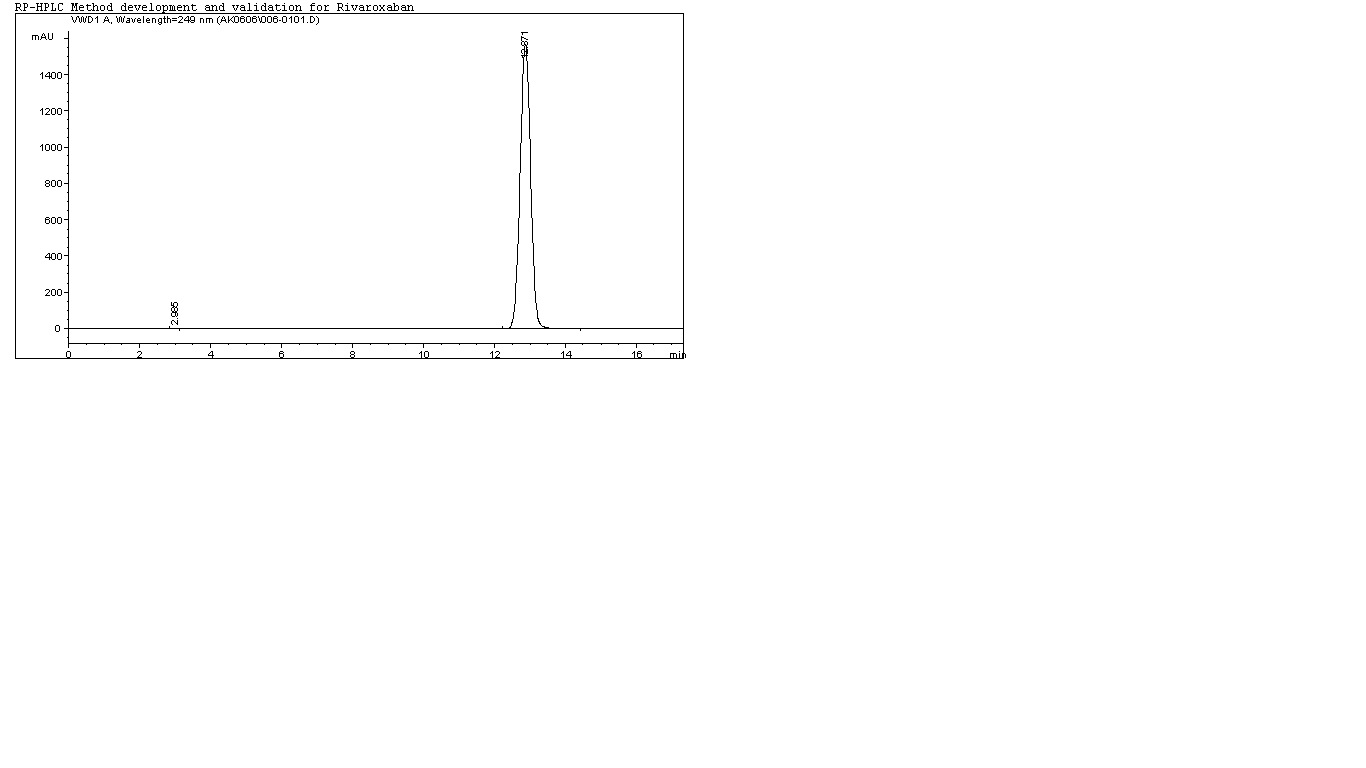** | **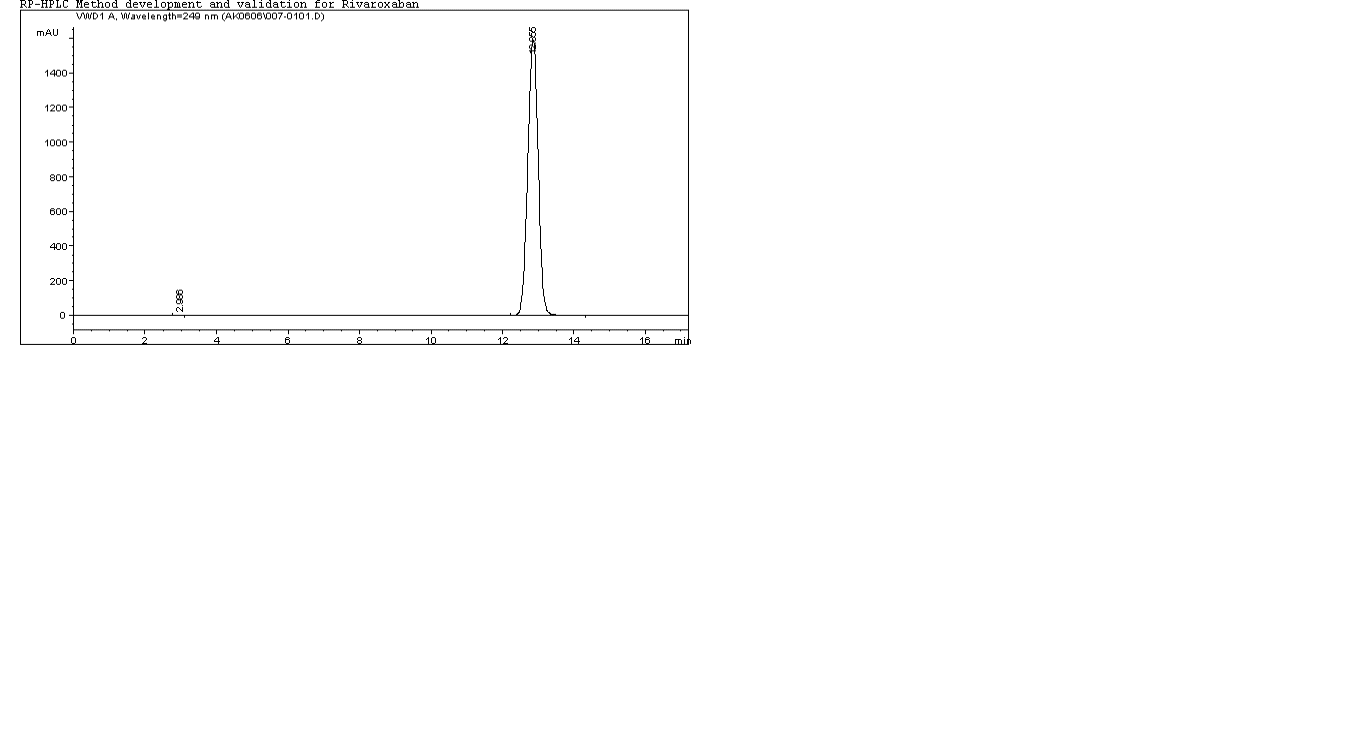** |

**Figure S15.** Chromatograms for Intermediate Precision Study for Rivaroxaban 700 ppm active ingredient

**Chromatographic conditions:** Isocratic elution, mobile phase 30:70 ACN/25 mM potassium phosphate buffer monobasic pH 2.9, flow rate 1.0 mL/min, detection wavelength at 249 nm, ambient temperature, 15 µL injection volume, thermo hypersil ODS C_18_ (4.6x250 mm, 5µm) column.

## Supplementary Tables

**Table S1.** Solubility study results of Rivaroxaban

| **Solvent** | **Solubility** | **Solvent** | **Solubility** |
| --- | --- | --- | --- |
| 100 % H2O | Not Soluble | 25:75 MeOH: H2O | Not Soluble |
| 100 % ACN | Not Soluble | 50:50 MeOH: H2O | Not Soluble |
| 100 % MeOH | Not Soluble | 75:25 MeOH: H2O | Not Soluble |
| 25:75 ACN: H_2_O | Not Soluble | 70:30 ACN: H2O | Soluble |
| 50:50 ACN: H_2_O | Partial Soluble | 75:25 ACN: H2O | Soluble |

**Table S2.** HPLC Columns Used for Method Development

| **Column #** | **Manufacturer** | **Length (mm)** | **Internal Diameter (mm)** | **Particle size (µm)** | **Serial Number** | **Part Number** |
| --- | --- | --- | --- | --- | --- | --- |
| 1 | Phenomenex C_18_ | 150 | 4.6 | 5 | 186837 | OOG-0391-EO |
| 2 | Water C_18_ | 150 | 4.6 | 5 | W21367N | 185001467 |
| 3 | Agilent Zorbax RX-C_18_ | 250 | 4.6 | 5 | USCU013381 | 880967-902 |
| 4 | WATER XTEERA RP-18 | 250 | 4.6 | 5 | W41751N | 186000496 |
| 5 | Phenomenex C_18_ | 250 | 4.6 | 5 | 169615 | 006-4041-E0 |
| 6 | Thermo ODS Hypersil C_18_ | 250 | 4.6 | 5 | 0153571S | 30105-254630 |

**Table S3.** Summary of pH Selection

| pH | Retention Time | Tailing Factor | Number of Theoretical Plates |
| --- | --- | --- | --- |
| 2.9 | 11.45 | 0.94 | 7890 |
| 5 | 11.46 | 0.94 | 7565 |
| 7 | 11.18 | 0.96 | 7695 |

**Table S4.** Summary of Solvent Strength Optimization

| Buffer/ ACN | Retention Time (minute) | Tailing Factor | Number of Theoretical Plates |
| --- | --- | --- | --- |
| 50 / 50 | 4.55 | 2.02 | 4243 |
| 60 / 40 | 8.87 | 1.52 | 4639 |
| 70 / 30 | 12.14 | 0.93 | 9272 |

**Table S5.** Solution Stability Study Results for Rivaroxaban 700 ppm Over a Period of 72 hours

| Concentration | Injected Time (hours) | Number of theoretical plates | Peak Area | % Change |
| --- | --- | --- | --- | --- |
| 700 | Immediately | 10672 | 29894.4 | ---- |
| 700 | 24 | 9333 | 29887.2 | - 0.024 |
| 700 | 48 | 9376 | 29827.0 | - 0.225 |
| 700 | 72 | 9820 | 29806.2 | - 0.295 |

**Table S6.** Method Precision Results for Rivaroxaban Active Ingredients

| Preparation Sample | Concentration (ppm) | Peak Area | Tailing factor | Number of Theoretical plate |
| --- | --- | --- | --- | --- |
| 1 | 700 | 30287.4 | 1.038 | 9940 |
| 2 | 700 | 30839.5 | 1.044 | 9967 |
| 3 | 700 | 30289.6 | 1.045 | 9867 |
| 4 | 700 | 30543.2 | 1.050 | 9838 |
| 5 | 700 | 30276.8 | 1.047 | 9967 |
| 6 | 700 | 30792.1 | 1.045 | 10343 |
| Average | | 30504.77 |  | |
| Standard Deviation | | 261.380 |  |  |
| %RSD | | 0.86 |  |  |

**Table S7:** Injection Precision Results for Rivaroxaban Active Ingredients.

| Sample injection | Concentration (ppm) | Peak Area | Tailing factor | Number of theoretical plate |
| --- | --- | --- | --- | --- |
| 1 | 700 | 32592 | 1.010 | 9038 |
| 2 | 700 | 32612 | 1.014 | 9167 |
| 3 | 700 | 32489 | 1.016 | 9198 |
| 4 | 700 | 32783 | 1.011 | 9276 |
| 5 | 700 | 32552 | 1.017 | 8879 |
| 6 | 700 | 32553 | 1.013 | 9084 |
| Average | | 32596.83 |  | |
| Standard Deviation | | 100.4697 |  |  |
| %RSD | | 0.31 |  |  |

**Table S8.** Intermediate Precision Results for Rivaroxaban Active Ingredients.

| Sample # | Concentration (ppm) | Peak Area | Retention Time (min) | Tailing Factor | Number of Theoretical Plates |
| --- | --- | --- | --- | --- | --- |
| 1 | 700 | 32203.0 | 13.06 | 1.13 | 11536 |
| 2 | 700 | 32399.4 | 12.89 | 0.97 | 8218 |
| 3 | 700 | 32280.2 | 12.88 | 0.98 | 8698 |
| 4 | 700 | 32351.3 | 12.91 | 1.00 | 8732 |
| 5 | 700 | 32285.5 | 12.87 | 1.01 | 9221 |
| **6** | 700 | 32197.1 | 12.85 | 1.03 | 9488 |
| Average | | 32286.08 | 12.91 |  | |
| Standard Deviation | | 79.94 | 0.076 |  |  |
| %RSD | | 0.25 | 0.60 |  |  |
